# Elevated WTAP promotes hyperinflammation by increasing m^6^A modification in inflammatory disease models

**DOI:** 10.1172/JCI177932

**Published:** 2024-05-16

**Authors:** Yong Ge, Rong Chen, Tao Ling, Biaodi Liu, Jingrong Huang, Youxiang Cheng, Yi Lin, Hongxuan Chen, Xiongmei Xie, Guomeng Xia, Guanzheng Luo, Shaochun Yuan, Anlong Xu

**Affiliations:** 1 Guangdong Province Key Laboratory of Pharmaceutical Functional Genes, MOE Key Laboratory of Gene Function and Regulation, State Key Laboratory of Biocontrol, School of Life Sciences, Sun Yat-Sen University, Guangzhou, China.; 2 Southern Marine Science and Engineering Guangdong Laboratory (Zhuhai), Zhuhai, China.; 3 Laboratory for Marine Biology and Biotechnology, Qingdao Marine Science and Technology Center, Qingdao, China.; 4 School of Life Sciences, Beijing University of Chinese Medicine, Beijing, China.

**Keywords:** Immunology, Inflammation, Innate immunity, Macrophages, RNA processing

## Abstract

Emerging evidence has linked the dysregulation of *N*^6^-methyladenosine (m^6^A) modification to inflammation and inflammatory diseases, but the underlying mechanism still needs investigation. Here, we found that high levels of m^6^A modification in a variety of hyperinflammatory states are p65-dependent because Wilms tumor 1–associated protein (WTAP), a key component of the “writer” complex, is transcriptionally regulated by p65, and its overexpression can lead to increased levels of m^6^A modification. Mechanistically, upregulated WTAP is more prone to phase separation to facilitate the aggregation of the writer complex to nuclear speckles and the deposition of m^6^A marks on transcriptionally active inflammatory transcripts, thereby accelerating the proinflammatory response. Further, a myeloid deficiency in WTAP attenuates the severity of LPS-induced sepsis and DSS-induced IBD. Thus, the proinflammatory effect of WTAP is a general risk-increasing mechanism, and interrupting the assembly of the m^6^A writer complex to reduce the global m^6^A levels by targeting the phase separation of WTAP may be a potential and promising therapeutic strategy for alleviating hyperinflammation.

## Abstract 2

High m^6^A modification induced by elevated<strong> </strong>WTAP is a proinflammatory risk factor for hyperinflammation in inflammatory diseases.

## Graphical abstract

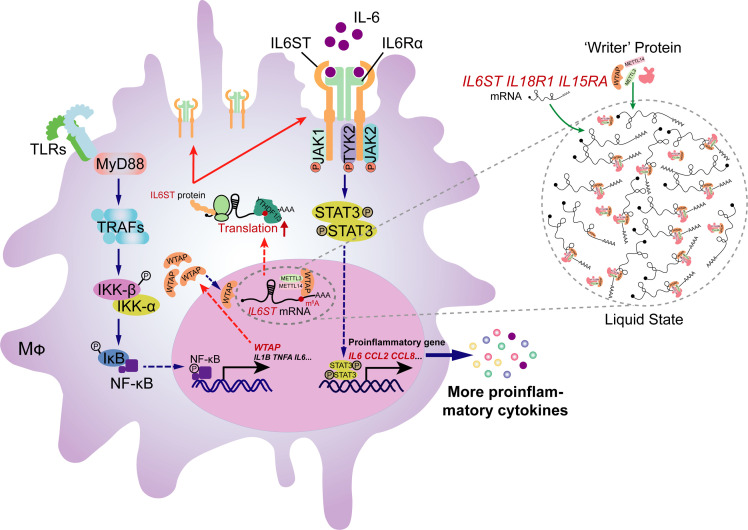

## Introduction

Inflammation is usually a physiological healing response that is triggered by noxious stimuli and conditions such as infection and tissue injury (1). Moderate inflammation is essential for pathogen clearance, tissue repair, and regeneration. However, dysregulated inflammation disrupts immune homeostasis, which may lead to the development of a variety of inflammatory diseases, such as chronic inflammation, metabolic disorders, autoimmune diseases (ADs), and cancer (2). The activation or induction of proinflammatory regulators and effectors must be precisely controlled at multiple levels, including the transcriptional (3), posttranscriptional (4),and posttranslational (5) levels to maintain immune homeostasis and prevent harmful outcomes, and these processes are thus potential targets for the treatment of inflammatory diseases (6, 7). Therapeutic strategies for inflammatory diseases have been established by developing antagonists or inhibitors against principal inflammatory effectors (NF-κB, STAT3, and JAK) and multifunctional proinflammatory cytokines (IL-6, TNF-α, IL-17A and IL-23) (8, 9). However, due to pleiotropy, the therapeutic effects of targeting specific cytokines, such as IL-6 or IL-17A, vary greatly among different inflammatory diseases (10). Likewise, small-molecule inhibitors targeting the NF-κB and STAT3 signaling pathways lead to significant side effects that alter the homeostasis of the immune system and the nonimmune cells (11, 12). Therefore, in-depth research into how inflammation is precisely regulated remains particularly important for the development of new treatments for excessive inflammatory responses.

In the past 2 decades, studies on immune regulation have focused mainly on posttranslational modifications of proteins, such as phosphorylation and ubiquitination (5). Recently, emerging evidence has strongly indicated that immune signaling can trigger dynamic alterations to the epitranscriptome, which orchestrates the regulation of the immune response (13, 14). Among those dynamic epitranscriptomic alterations, *N*^6^-methyladenosine (m^6^A) modification is the most abundant and reversible RNA modification and can influence premRNA splicing, stability, translation, location, and transport (15). The m^6^A modification is mediated by a methyltransferase complex called “writer” (METTL3, METTL14, WTAP, VIRMA, ZC3H13, and RBM15) and removed by demethylase, namely “eraser” (FTO and ALKBH5) (16). Among them, Wilms tumor 1–associated protein (WTAP) may function as a regulatory subunit that binds to METTL3/14 and is required for substrate recruitment and METTL3/14 localization (17). In addition to playing roles in distinct biological processes such as embryonic development, hematopoiesis, and cancer, the roles of m^6^A modification in inflammatory regulation have recently attracted intense attention (18, 19). Principal inflammatory signaling pathways, such as NF-κB, JAK-STAT, and MAPK pathways, are extensively regulated by m^6^A modification, while m^6^A-related proteins have different or even opposite regulatory effects on inflammatory responses by targeting different genes or depending on specific cell and disease statuses (19, 20). For example, depleting METTL3 decreases the expression of TRAF6 by entrapping the transcripts in the nucleus, which inhibits the activation of the NF-κB and MAPK signaling pathways (21). In contrast, another study revealed that high METTL3 expression attenuates the inflammatory response in macrophages (22). Further, knocking down the demethylase FTO leads to an increased STAT3 phosphorylation and proinflammatory cytokine secretion (23), whereas knocking down ALKBH5 inhibits the production of inflammatory cytokines (24). In addition to m^6^A writers and erasers, m^6^A readers also play different roles in regulating different inflammatory events. For example, a previous study showed that YTHDF2 accelerates the decay of m^6^A-modified transcripts encoding NF-κB–negative regulators to regulate NF-κB signaling in intratumoral Treg cells (25). However, another study indicated that YTHDF2 is an inflammatory suppressor that downregulates the expression of m^6^A-modified transcripts in inflammatory response and thereby protects hematopoietic stem cells from excessive proinflammatory signals (26). Thus, although notable evidence clearly indicates a role for the m^6^A modification in modulating inflammatory responses, its effects appear to depend greatly on the disease status or specific target genes. Recently, due to the finding of aberrant RNA methylation in inflammatory diseases (19) and cancers (27), small-molecule inhibitors, which target specific writers or erasers to reprogram the m^6^A epitranscriptome, have attracted considerable attention and have been proven to be feasible (28). Hence, further identification of the intrinsic principles underlying the mechanism through which m^6^A regulates inflammation and inflammatory diseases are extremely important before a therapeutic approach can be developed to target the m^6^A mark.

By revealing the basic principle of m^6^A modification in regulating inflammation, we report a function of the m^6^A writer protein WTAP in controlling inflammatory responses and associated diseases. We found that WTAP is an NF-κB p65-stimulated gene and that its expression is markedly upregulated in response to a variety of inflammatory stimuli and in many inflammatory diseases. Mechanistically, after an increase in its concentration, WTAP spontaneously undergoes phase separation, which facilitates the aggregation of the writer complex to nuclear speckles and the deposition of m^6^A on transcriptionally active proinflammatory genes. Hence, WTAP enhances the protein synthesis of many m^6^A-modified proinflammatory transcripts, including IL6ST, IL18R1 and IL15RA, which accelerates inflammatory responses and exacerbates the severity of many inflammatory diseases. Thus, our study here identified WTAP as a risk factor for inflammatory responses and thereby provides mechanistic insights into WTAP as a potential therapeutic target for preventing excessive inflammation.

## Results

### Hyperinflammation is accompanied by elevated WTAP levels in many inflammatory diseases.

To reveal how m^6^A modification plays an essential role in inflammation and inflammatory diseases, we analyzed data obtained from the Gene Expression Omnibus (GEO) data sets (GSE13887/137268/69063/97779/166388/208303, Supplemental Table 1; supplemental material available online with this article; https://doi.org/10.1172/JCI177932DS1), and found that among m^6^A-related proteins, WTAP expression is commonly upregulated in patients with systemic lupus erythematosus (SLE), asthma, sepsis, rheumatoid arthritis (RA), psoriasis, or Crohn’s disease (Supplemental Figure 1, A–F). We then performed statistical analyses of previously published microarray data sets (GSE19315/198326/2411/2638/227851/189847, Supplemental Table 1) to verify this phenomenon and further revealed that only the abundance of *WTAP* mRNA among the members of the writer complex was substantially increased after LPS stimulation of THP-1 cells (Supplemental Figure 1G) and human macrophages (Supplemental Figure 1H). Similar results were obtained from the lung tissue of mice with LPS-induced lung injury (Supplemental Figure 1I). In addition, we found that the *WTAP* mRNA abundance was also increased in TNF-α–stimulated human microvascular endothelial cells (HMECs) (Supplemental Figure 1J), *Mycobacterium tuberculosis* (H37Rv)–infected THP-1 cells (Supplemental Figure 1K) and *Salmonella typhimurium* (SL1344)–infected macrophages (Supplemental Figure 1L). These observations suggested that the overexpression of WTAP is a common phenomenon in hyperinflammatory states.

**Figure 1:**
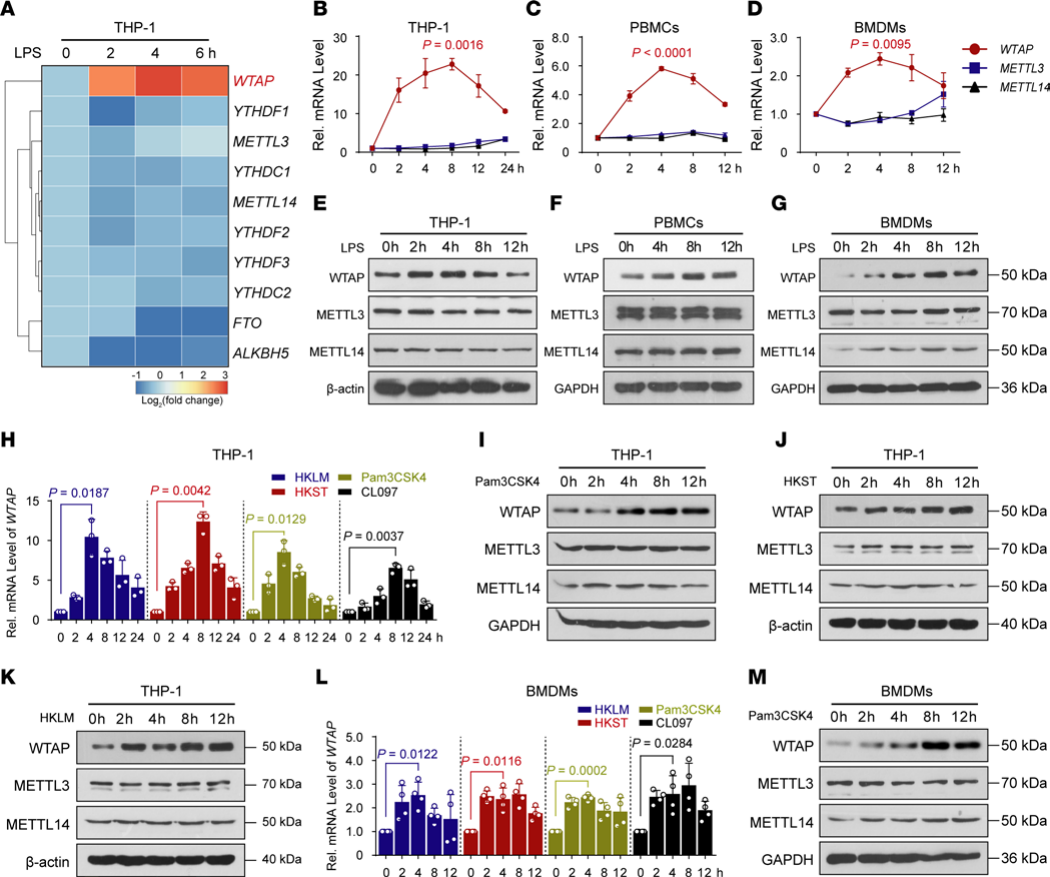
Multiple inflammatory stimuli markedly increase the expression of WTAP in macrophages. (**A**) Heatmap showing a change in the mRNA abundance of m^6^A-related genes in THP-1 cells stimulated with lipopolysaccharide (LPS) at the indicated time points. (**B–D**) qRT-PCR showing the mRNA abundance of the members of writer complex in THP-1 cells (**B**), PBMCs (**C**) and BMDMs (**D**) stimulated with LPS at the indicated time points. (**E–G**) Immunoblots showing the protein abundance of the METTL3/METTL14/WTAP heterotrimer in THP-1 cells (**E**), PBMCs (**F**), and BMDMs (**G**) stimulated with LPS at the indicated time points. (**H)** qRT-PCR showing the mRNA abundance of WTAP in THP-1 cells stimulated with CL097, Pam3CSK4, heat-killed *Salmonella typhimurium* (HKST), or heat-killed *Listeria monocytogenes* (HKLM) at the indicated time points. (**I–K**) Immunoblots showing the protein abundance of the METTL3/METTL14/WTAP heterotrimer in THP-1 cells stimulated with Pam3CSK4 (**I**), HKST (**J**) or HKLM (**K**) at the indicated time points. (**L**) qRT-PCR showing the mRNA abundance of WTAP in BMDMs stimulated with CL097, Pam3CSK4, HKST, or HKLM at the indicated time points. (**M**) Immunoblots showing the protein abundance of the METTL3/METTL14/WTAP heterotrimer in BMDMs stimulated with Pam3CSK4 at the indicated time points. Data are presented as the mean ± SD in (**B**–**D**, **H**, and **L**), with individual measurements overlaid as dots, statistical analysis was performed using 2-tailed Student’s *t* test. Data are representative of 3 independent biological experiments in (**E**–**G**, **I**–**K**, and **M**).

To further verify the above-mentioned observations, THP-1 cells were treated with the Toll-like receptor 4 (TLR4) agonist LPS, an ideal reagent for activating the inflammatory signaling cascade in vivo (29). RNA-Seq analysis was then performed, and the results confirmed that only the abundance of *WTAP* mRNA was substantially upregulated in the LPS-stimulated THP-1 cells among the members of the writer complex (Figure 1A). Further qRT–PCR (Figure 1, B–D) or immunoblotting (Figure 1, E–G) analyses of THP-1 cells, peripheral blood mononuclear cells (PBMCs), and mouse bone marrow–derived macrophages (BMDMs) confirmed that the WTAP expression was observably upregulated upon LPS stimulation. To determine whether the upregulated expression of WTAP is ubiquitous upon specific inflammatory stress, we treated THP-1 cells and BMDMs with different TLR agonists or heat-killed bacteria, such as CL097 (a TLR7/8 ligand), Pam3CSK4 (a TLR1/2 ligand), heat-killed *Salmonella typhimurium* (HKST), and heat-killed *Listeria monocytogenes* (HKLM), and found that the WTAP mRNA and protein abundances were both substantially increased (Figure 1, H–M). Thus, high WTAP expression is positively correlated with hyperinflammatory states, implying a potential role for WTAP in the regulation of inflammation.

### The upregulation of WTAP in hyperinflammation is controlled by NF-κB p65.

**Figure 2:**
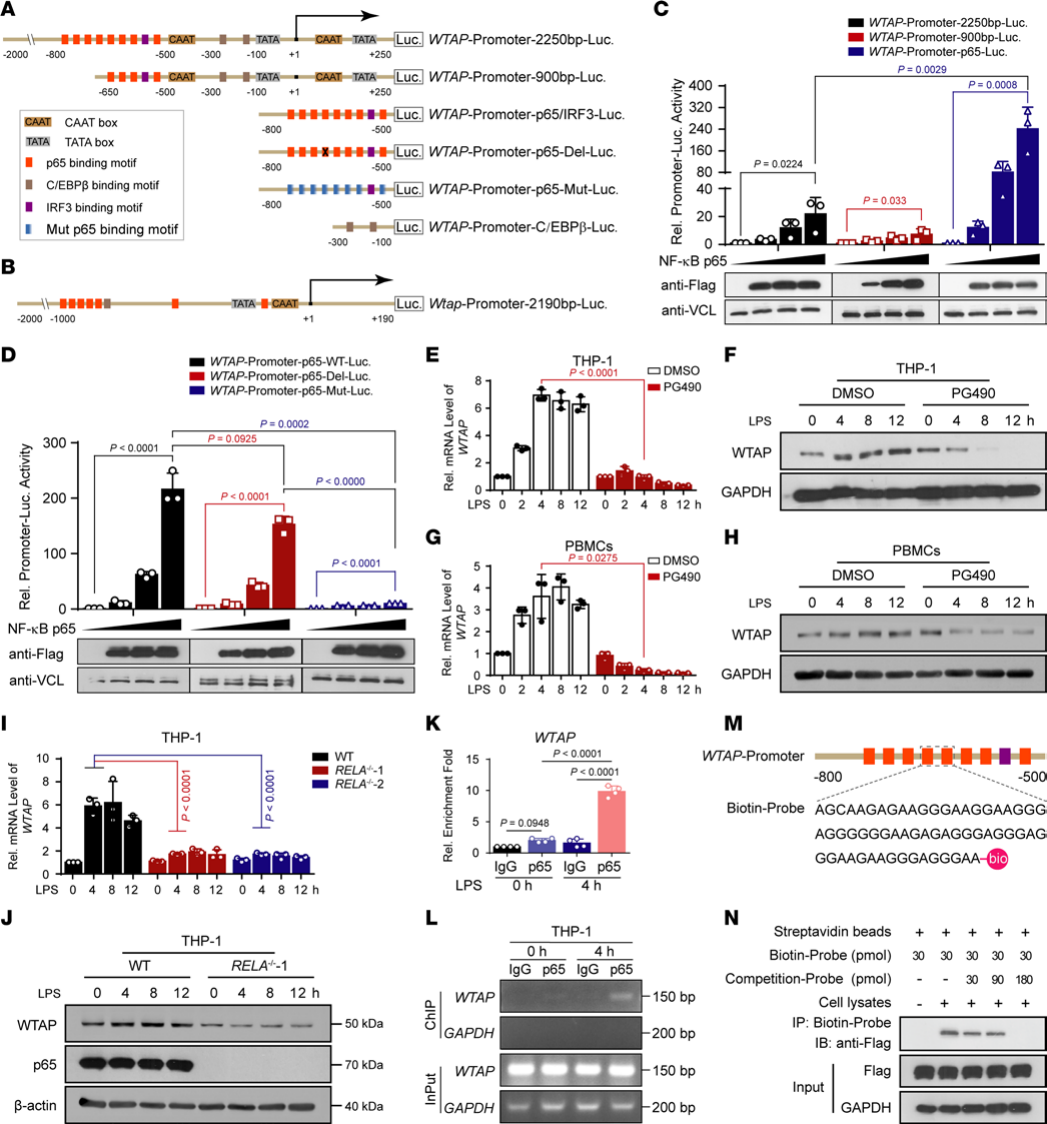
The upregulation of WTAP in hyperinflammation is controlled by NF-κB p65. (**A**) Schematic of the full-length, mutant, and truncated human *WTAP* promoter. (**B**) Schematic of the mouse *Wtap* promoter. (**C** and **D**) Luciferase activity analyses in 293T cells transfected with a luciferase reporter for the *WTAP* promoter with WT (**C**), mutant, or truncated (**D**) p65-binding motif, together with increasing doses of Flag-tagged NF-κB p65. (**E–H**) qRT-PCR or immunoblots showing the expression of WTAP in THP-1 cells (**E** and **F**) or PBMCs (**G** and **H**) pretreated with PG490, followed by stimulation with LPS at the indicated time points. (**I** and **J**) qRT-PCR (**I**) or immunoblots (**J**) showing the expression of WTAP in WT and *RELA*^–/–^ THP-1 cells stimulated with LPS at different time points. (**K** and **L**) ChIP-qPCR (**K**) and semi-quantitative RT–PCR (**L**) assays analyzing p65 occupancy on the WTAP promoter in THP-1 cells before and after LPS treatment. (**M**) Schematic showing the biotin-labeled *WTAP* promoter probes. (**N**) DNA pull-down assays showing the effect of NF-κB p65 binding with *WTAP* promoter probes. Data are presented as the mean ± SD in **C**–**E**, **G**, **I**, and **K**, with individual measurements overlaid as dots. Statistical analysis was performed using 2-tailed Student’s *t* test in **E** and **G** or 1-way ANOVA multiple comparisons in **C**, **D**, **I**, and **K**. Data are representative of 3 independent biological experiments in (**C**, **D**, **F**, **H**, **J**, **L**, and **N**).

Protein abundance can be regulated at the transcriptional, translational, or posttranslational level. To reveal the specific mechanism underlying the upregulation of WTAP at both the mRNA and protein levels upon inflammatory stimulation, bioinformatics analyses using the Promoter 2.0 prediction server (http://www.cbs.dtu.dk/services/promoter/), CpG plot (http://www.ebi.ac.uk/Tools/seqstats/emboss_cpgplot/), and JASPAR (http://jaspar.genereg.net/) were performed and identified the region between −800 and +250 in the genomic sequence of human *WTAP* containing the TATA box, CAAT box, and GC box, which are the characteristics of promoters. Further analyses of transcription factor binding sites revealed that the *WTAP* promoter contains NF-κB p65, C/EBP-β, IRF3, STAT3, and HIF1-α binding motifs (Supplemental Table 2). Studies have reported that both STAT3 and HIF1-α can transcriptionally upregulate the expression of WTAP in some cancer cells (30, 31), but we found that LPS-induced upregulation of WTAP was not affected by SC144 treatment, which inhibited the activation of STAT3 signaling by binding to IL6ST (32) (Supplemental Figure 2, A and B). Similarly, CoCl_2_-induced HIF1-α accumulation upregulated WTAP expression (Supplemental Figure 2C), but inflammatory stimuli did not cause HIF1-α accumulation (Supplemental Figure 2D). These data indicated that STAT3 and HIF1-α have little effect on the transcriptional upregulation of WTAP under inflammatory stress. Therefore, we next explored the transcriptional activation of WTAP by NF-κB p65, C/EBP-β and IRF3, and the p65 binding motifs exhibited the highest prevalence and were distributed in a relatively concentrated and overlapping regions (Figure 2A). Similar results were obtained from an analysis of the mouse *Wtap* promoter region (Figure 2B). As shown in Figure 2, A and B, we constructed a series of reporter plasmids containing the *WTAP* promoter with WT, mutated, or deleted NF-κB p65, C/EBP-β, and IRF3 binding motifs based on the pGL3 basic construct. These reporter plasmids were then cotransfected with increasing amounts of NF-κB p65-, C/EBP-β- or IRF3-expressing plasmids into 293T cells. The results revealed that NF-κB p65 and C/EBP-β, but not IRF3, increased the expression of the respective *WTAP* promoter reporters in a dose-dependent manner (Figure 2C and Supplemental Figure 2, E and F). However, deletion or mutation of the core p65 binding sites in the *WTAP* promoter inhibited the expression of the reporter gene (Figure 2D). Similar results were obtained using reporter plasmids containing the mouse *Wtap* promoter (Supplemental Figure 2G). Further, the treatment of THP-1 cells or PBMCs with the p65 inhibitor PG490 (triptolide) inhibited the induction of WTAP expression by LPS (Figure 2, E–H). Consistently, using SN50, inhibitor of NF-κB p50 translocation (33), we obtained the same results (Supplemental Figure 2, H and I). Next, we generated *RELA*^–/–^ (encoding p65) THP-1 cells using the CRISPR-mediated genome editing approach (Supplemental Figure 2J) and found that the upregulation of WTAP was inhibited in *RELA*^–/–^ THP-1 cells after stimulation with LPS, Pam3CSK4, HKST, or HKLM (Figure 2, I and J and Supplemental Figure 2, K–N). Using primers targeting p65 binding sites in the *WTAP* promoter for ChIP–qPCR, we found a marked enrichment of p65 binding sequence immunoprecipitated by the p65 antibody compared with that of the negative control IgG after LPS treatment (Figure 2, K and L and Supplemental Figure 2O). DNA pull-down assays also confirmed the direct binding of Flag-tagged p65 to biotin-labeled p65 binding probes of the *WTAP* promoter in vitro (Figure 2, M and N). In addition, C/EBP-β could also slightly activate the WTAP expression (Supplemental Figure 2E), and DNA pull-down assays confirmed the direct binding of C/EBP-β to the *WTAP* promoter (Supplemental Figure 2P). Because p65 can further activate inducible transcription factors such as ATF3, C/EBP-δ and C/EBP-β to enhance the LPS-induced transcriptional response (3), we hypothesized that although C/EBP-β may play a role in the transcription of *WTAP* mRNA, the upregulation of WTAP expression predominantly depends on the activation of NF-κB p65.

### WTAP positively regulates proinflammatory responses.

**Figure 3:**
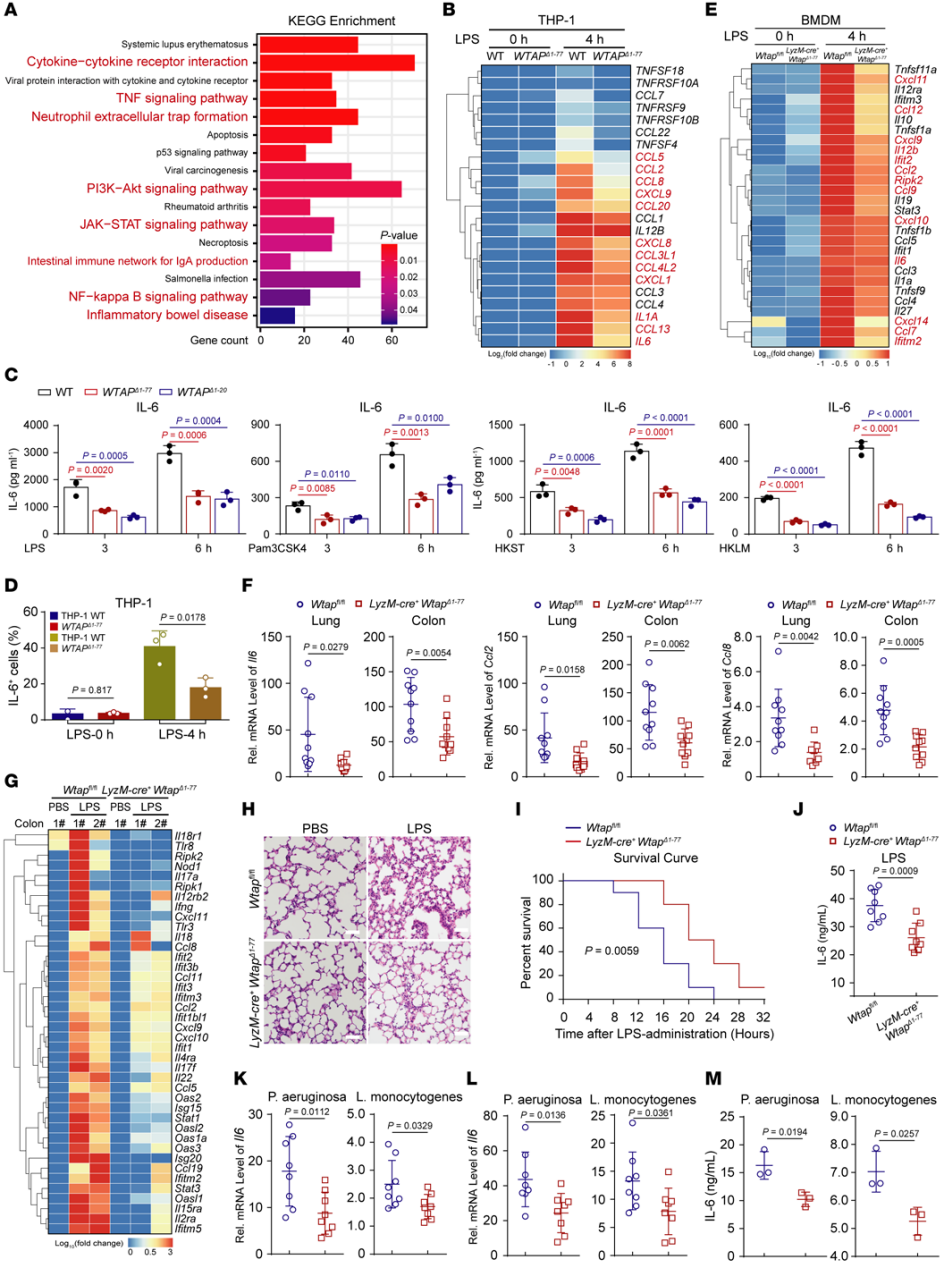
WTAP positively regulates proinflammatory responses. (**A**) KEGG analyses of downregulated genes in *WTAP*^Δ1–77^ THP-1 cells stimulated with LPS compared with WT cells. (**B**) Heatmap showing the mRNA abundance of proinflammatory cytokines in WT and *WTAP*^Δ1–77^ THP-1 cells stimulated with LPS. (**C**) ELISAs detecting IL-6 secretion in supernatants of WT and *WTAP*^Δ1–77^ THP-1 cells stimulated with inflammatory stimuli. (**D**) Flow cytometry showing IL-6 fluorescence intensity in WT and *WTAP*^Δ1–77^ THP-1 cells. (**E**) Heatmap showing the mRNA abundance of proinflammatory cytokines in WT and *Wtap*^Δ1–77^ BMDMs stimulated with LPS. (**F** and **G**) qRT–PCR (**F**) and heatmap (**G**) showing the mRNA abundance of proinflammatory cytokines in indicated tissues from *Wtap*^fl/fl^ and *LyzM-cre*^+^ *Wtap*^Δ1–77^ mice that were intraperitoneally injected with LPS for 12 hours. (**F**) *n =* 10 mice. (**H**) H&E assays showing the lung injury of *Wtap*^fl/fl^ and *LyzM-cre*^+^ *Wtap*^Δ1–77^ mice that were intraperitoneally injected with LPS for 6 hours. Scale bars: 50 μm. *n =* 4 mice. (**I**) The survival of *Wtap*^fl/fl^ and *LyzM-cre*^+^ *Wtap*^Δ1–77^ mice was monitored for 32 hours following the intraperitoneal injection of LPS. *n =* 10 mice. (**J**) ELISAs were performed to detect IL-6 secretion in serum from *Wtap*^fl/fl^ and *LyzM-cre*^+^ *Wtap*^Δ1–77^ mice that were intraperitoneally injected with LPS for 12 hours. *n =* 8 mice. (**K**–**M**) qRT–PCR showing the mRNA abundance of *Il6* in the lung (**K**) or colon (**L**) tissues or ELISAs detecting IL-6 secretion in serum (**M**) from *Wtap*^fl/fl^ and *LyzM-cre*^+^ *Wtap*^Δ1–77^ mice that were intraperitoneally injected with *P*. *aeruginosa* or *L*. *monocytogenes* for 16 hours. **K** and **L**, *n =* 8 mice. **M**, *n =* 3 mice. Data are presented as the mean ± SD in **C**, **D**, **F**, and **J**–**M**, with individual measurements overlaid as dots. Statistical analysis was performed using 1-way ANOVA multiple comparisons in **C**, or 2-tailed Student’s *t* test in **D**, **F**, and **J**–**M**. Data are representative of 3 independent biological experiments in **H**.

To determine the significance of WTAP in inflammatory responses, we first generated *WTAP*-knockout THP-1 and 293T cells using the CRISPR/Cas9 approach, and the *Wtap* conditional KO (CKO) mice by crossing *Wtap*^fl/fl^ mice with mice expressing Cre recombinase under the control of the lysozyme 2 promoter (*LyzM*-Cre) (Supplemental Figures 3 and 4). After genomic sequencing and a series of functional characterization, we found that the status of *WTAP*^Δ1–20^ and *WTAP*^Δ1–77^ cells should be very close to that of cells in which the protein is completely knocked out (Supplemental Figure 3 and 4). More details about the characterizations of the WTAP-deficient cells and mice are available in the Supplemental data. We next performed RNA-Seq analyses with WT and *WTAP*^Δ1–77^ THP-1 cells before and after stimulation with LPS. KEGG enrichment analyses revealed that the downregulated transcripts in *WTAP*^Δ1–77^ THP-1 cells were mainly enriched in the cytokine production and inflammatory signaling pathways (Figure 3, A and B). In contrast, the upregulated genes in *WTAP*^Δ1–77^ THP-1 cells were enriched in a few pathways that are not directly associated with inflammatory responses (Supplemental Figure 5A). To confirm the results obtained by RNA-Seq, qRT–PCR analyses were performed and revealed marked reductions in *IL6*, *CCL2*, *CCL8,* and *CXCL8* expression in *WTAP*^Δ1–77^ THP-1 cells after stimulation with LPS (Supplemental Figure 5B). Similar results were obtained with *WTAP*^Δ1–77^ THP-1 cells treated with Pam3CSK4, HKST, or HKLM (Supplemental Figure 5C). Consistently, compared with the WT cells, *WTAP*^Δ1–77^ THP-1 cells exhibited a significant reduction in IL-6 expression and secretion in response to the aforementioned stimuli (Figure 3, C and D). Further, ectopic expression of WTAP (Supplemental Figure 5D) substantially facilitated the expression of IL-6 and other inflammatory genes under inflammatory stimulation (Supplemental Figure 5, E and F). These results confirmed that WTAP accelerates inflammatory responses by promoting the expression of many proinflammatory cytokines in response to different inflammatory stimuli.

### WTAP aggravates LPS-induced sepsis in mice.

Because WTAP expression was upregulated after LPS stimulation and WTAP deficiency can reduce inflammatory responses in macrophages, we next examined the biological effects of WTAP on the progression of inflammatory diseases in *LyzM*-Cre^+^ *Wtap*^Δ1–77^ mice. BMDMs first isolated from *Wtap*^fl/fl^ and *LyzM*-Cre^+^ *Wtap*^Δ1–77^ mice were treated with LPS and then used for RNA-Seq analyses. The results showed that WTAP-deficient BMDMs exhibited a reduction in the expression of proinflammatory cytokines upon LPS stimulation (Figure 3E), which is consistent with the trend observed in THP-1 cells. As mentioned above, the abundance of the *WTAP* mRNA was higher in the PBMCs from patients with sepsis than in those from healthy individuals (Supplemental Figure 1C). A retrospective study of publicly available data also revealed that WTAP is a risk gene for sepsis, and its expression was correlated with 28-day cumulative mortality (34). Thus, we further investigated the biological effects of WTAP using *LyzM*-Cre^+^ *Wtap*^Δ1–77^ and *Wtap*^fl/fl^ mouse models of LPS-induced sepsis. Consistent with the results obtained for LPS-induced macrophages, the LPS-induced expression of *Il6*, *Ccl2,* and *Ccl8* was decreased in lung and colon tissues from *LyzM*-Cre^+^ *Wtap*^Δ1–77^ mice compared with *Wtap*^fl/fl^ mice (Figure 3F), whereas the expression of *Il1a*, *Il1b,* and *Tnfa* was not obviously different (Supplemental Figure 5, G and H). The same trend was obtained with RNA-Seq data from colon samples (Figure 3G). Notably, *LyzM*-Cre^+^ *Wtap*^Δ1–77^ mice exhibited less lung inflammation and fewer pathological manifestations of lung injury (Figure 3H) and were therefore more tolerant to LPS-induced fatal sepsis (Figure 3I) than their WT counterparts. Moreover, the serum IL-6 concentration in *LyzM*-Cre^+^ *Wtap*^Δ1–77^ mice was significantly reduced compared with their *Wtap*^fl/fl^ littermates (Figure 3J). Similarly, after intraperitoneal (i.p.) injection of *Pseudomonas aeruginosa* (*P*. *aeruginosa*) or *Listeria monocytogenes* (*L*. *monocytogenes*), the expression of *Il6*, *Ccl2,* and *Ccl8* in lung (Figure 3K and Supplemental Figure 5, I and J) and colon (Figure 3L and Supplemental Figure 5, K and L) tissues, as well as the secretion of IL-6 into serum (Figure 3M), decreased in *LyzM*-Cre^+^ *Wtap*^Δ1–77^ mice. Collectively, these data suggested that the increased expression of WTAP aggravates LPS- and bacteria-induced inflammatory responses in vivo.

### WTAP promotes the activation of STAT3 signaling through the m^6^A modification to accelerate inflammatory responses.

**Figure 4:**
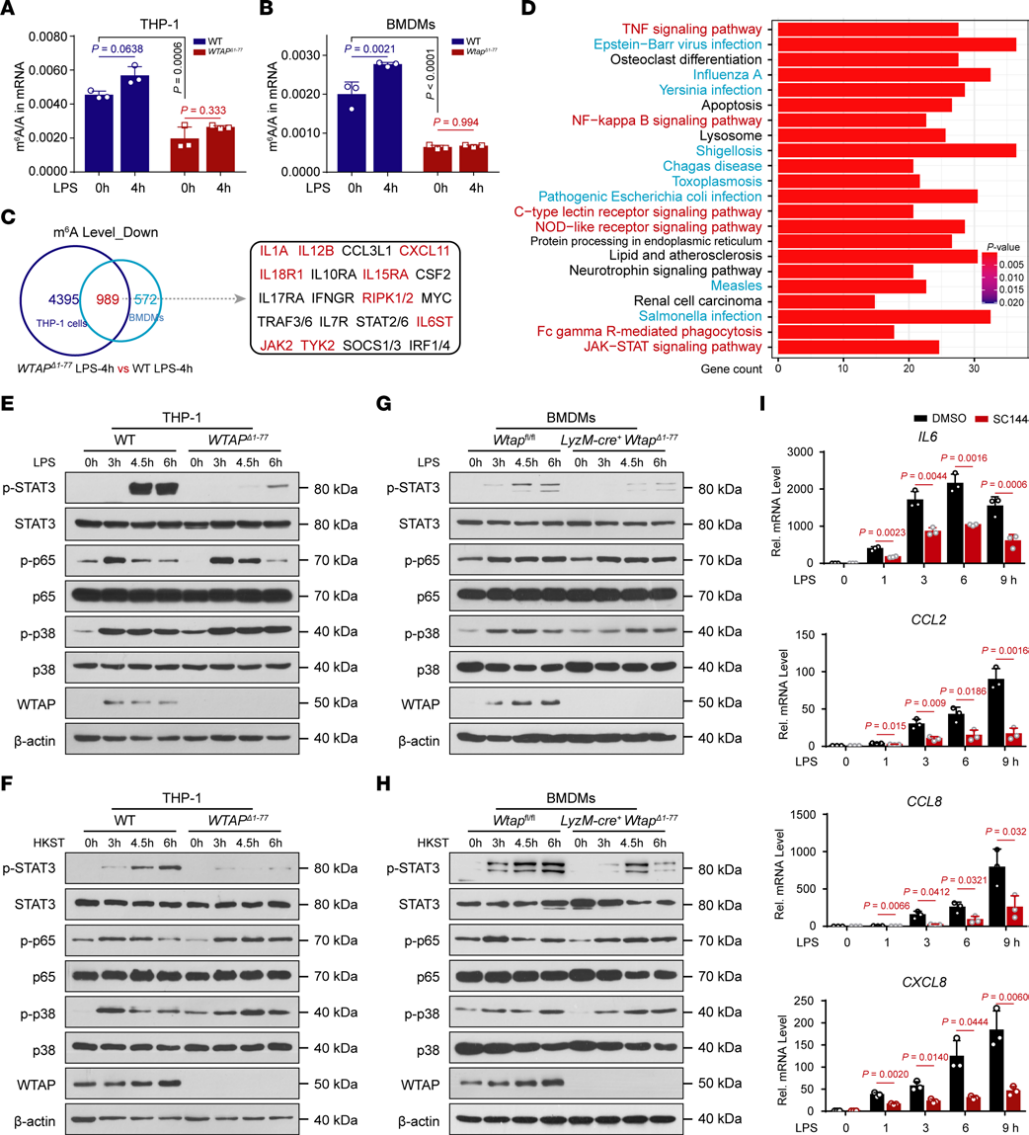
WTAP promotes the activation of STAT3 signaling through the m^6^A modification to accelerate inflammatory responses. (**A** and **B**) LC–MS/MS quantification of m^6^A abundance in mRNA extracted from WT and *WTAP*^Δ1–77^ THP-1 cells or *Wtap*^Δ1–77^ BMDMs with or without LPS treatment. (**C**) Venn diagrams showing transcripts with decreased m^6^A abundance in *WTAP*^Δ1–77^ THP-1 cells and *Wtap*^Δ1–77^ BMDMs compared with WT cells stimulated with LPS for 4 hours. (**D**) KEGG enrichment analysis of the overlapping transcripts presented in **C**. (**E**–**H**) Immunoblots showing total and phosphorylated STAT3, p65, and p38 levels in WT and *WTAP*^Δ1–77^ THP-1 cells (**E** and **F**) or BMDMs (**G** and **H**) stimulated with LPS or HKST at the indicated time points. (**I**) qRT–PCR showing the expression of *IL6*, *CCL2*, *CCL8,* and *CXCL8* in THP-1 cells treated with SC144, followed by stimulation with LPS at the indicated time points. Data are presented as the mean ± SD in **A**, **B**, and **I**, with individual measurements overlaid as dots. Statistical analysis was performed using 1-way ANOVA multiple comparisons in **A** and **B**, or 2-tailed Student’s *t* test in **I**. Data are representative of 3 independent biological experiments in **E**–**H**.

As a key m^6^A writer, WTAP is required for anchoring METTL3 and other cofactors to nuclear speckles to modulate m^6^A modification of RNA (17). To determine whether WTAP affects the inflammatory responses through m^6^A modification, we first quantified m^6^A abundance in cells by liquid chromatography–mass spectrometry (LC–MS/MS) assays and found that the overall level of the m^6^A modification increased in WT cells but not in WTAP-deficient cells after LPS stimulation (Figure 4, A and B). These data suggested that the upregulation of WTAP can improve the overall m^6^A modification abundance during inflammatory stimulation, which is consistent with the previous conclusion that WTAP is responsible for the observed increase in m^6^A modification upon bacterial infection (35).

We then performed methylated RNA immunoprecipitation sequencing (MeRIP-Seq) analyses to map mRNA transcripts with different m^6^A peaks accompanied by different mRNA levels in WT and *WTAP*^Δ1–77^ THP-1 cells or *Wtap*^Δ1–77^ BMDMs after LPS stimulation. According to the MeRIP-Seq data, the consensus m^6^A core motifs were enriched in the m^6^A peaks in all the samples (Supplemental Figure 6A). A peak distribution analysis revealed that the m^6^A sites were enriched in both exons and 3′UTRs (Supplemental Figure 6B), with the greatest enrichment near the stop codon (Supplemental Figure 6, C and D). Moreover, the MeRIP-Seq analysis revealed that the m^6^A peaks of nearly 5,000 transcripts in *WTAP*^Δ1–77^ THP-1 cells or more than 1,500 transcripts in *Wtap*^Δ1–77^ BMDMs were lower than in WT cells upon LPS treatment, respectively (Figure 4C). These identified transcripts were enriched in many inflammatory signaling pathways, including the NF-κB (TNF-α/TLR/NLR/CLR-mediated), JAK-STAT, and MAPK pathways (Supplemental Figure 6, E and F). We further compared the transcripts with reduced m^6^A marks after WTAP deletion between THP-1 cells and BMDMs treated with LPS and identified 989 overlapping transcripts (Figure 4C). KEGG enrichment analyses revealed that these overlapping transcripts were closely related to bacterial infection and inflammatory signaling pathways (Figure 4D), such as *IL6ST*, *IL15RA*, *IL18R1*, *TYK2*, *JAK2*, *RIPK2*, *IL1A*, *IL12B,* and *CXCL11* (Figure 4C).

Since both RNA-Seq and MeRIP-Seq indicated that WTAP is closely related to inflammatory pathways, such as the NF-κB, JAK-STAT, and MAPK pathways, we explored the effect of WTAP on the activation of these pathways. The results showed that the phosphorylation of STAT3, but not that of p65 or p38, was substantially decreased in *WTAP*^Δ1–77^ THP-1 cells in response to LPS or HKST treatment (Figure 4, E and F and Supplemental Figure 6, G and H). The same treatment was applied to BMDMs from *Wtap^fl/fl^* or *LyzM*-Cre^+^ *Wtap*^Δ1–77^ mice to further confirm this effect, and similar results were obtained (Figure 4, G and H and Supplemental Figure 6, G and H). Consistently, i.p. injection of LPS increased the level of phosphorylated STAT3 (Supplemental Figure 7A). However, this induction was substantially reduced in the lung and colon tissues from *LyzM*-Cre^+^ *Wtap*^Δ1–77^ mice (Supplemental Figure 7A). In contrast, ectopic expression of WTAP in *WTAP*^Δ1–77^ THP-1 cells substantially increased the level of phosphorylated STAT3 after LPS stimulation (Supplemental Figure 7B). We further found that the LPS-induced expression of *IL6*, *CCL2*, *CCL8,* and *CXCL8* (Figure 4I), but not that of *TNFA*, *IL1B*, and *LTA* (Supplemental Figure 7C), was markedly reduced after pretreatment with the gp130 (IL6ST) inhibitor SC144. Interestingly, although the expression of many proinflammatory cytokines was reduced in *WTAP*^Δ1–77^ THP-1 cells, the expression and secretion of IL-1β, TNF-α, or LTA (TNF-β) did not obviously change compared with those of WT THP-1 cells in response to the indicated stimuli (Supplemental Figure 7, D–G), which is consistent with the fact that p65 activation is not affected by WTAP. Together, these data suggested that WTAP-mediated m^6^A modification is deeply involved in the regulation of bacterial infection and inflammatory response in both humans and mice and may accelerate inflammatory responses by enhancing the STAT3 signaling axis.

### WTAP promotes the protein expression of proinflammatory genes through m^6^A modification.

**Figure 5:**
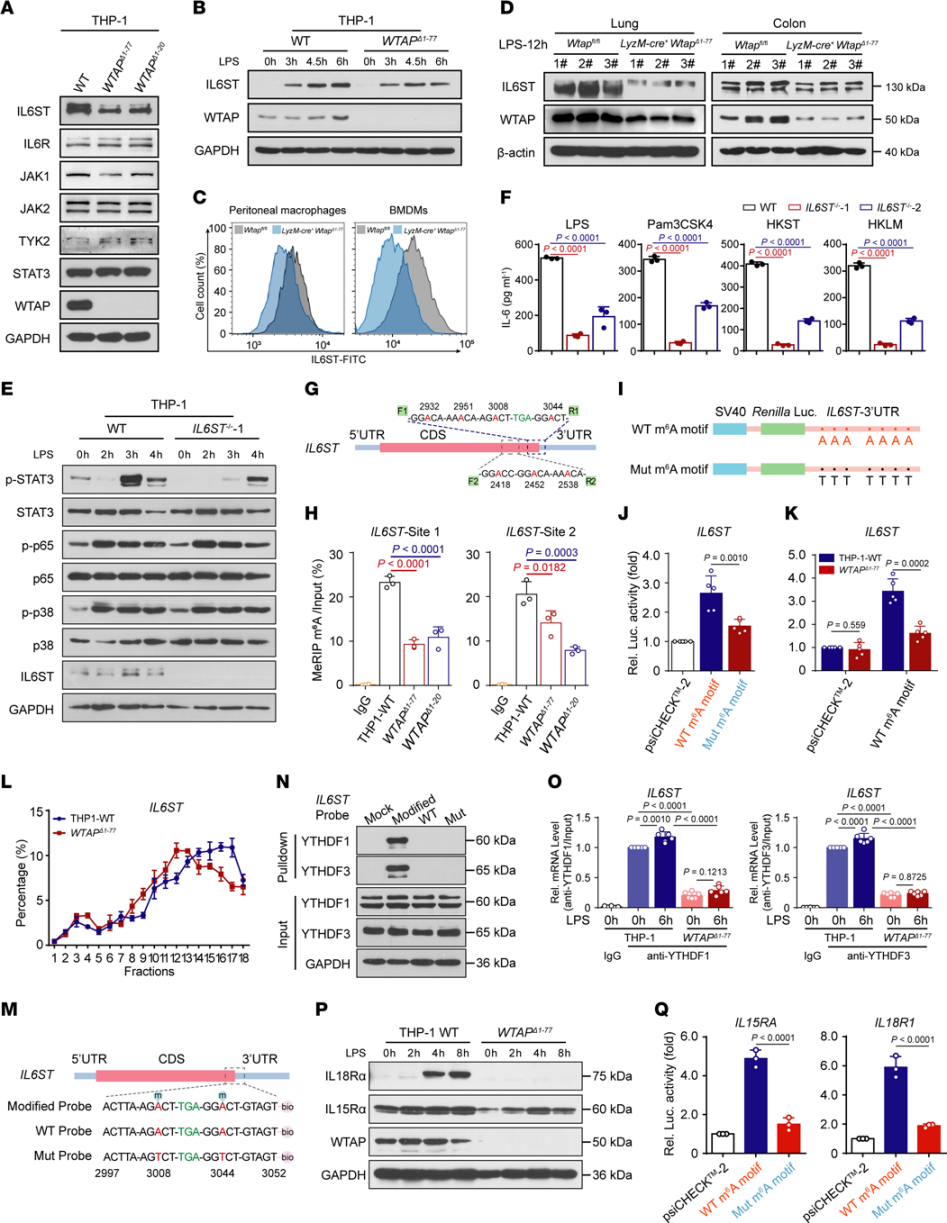
WTAP promotes the protein expression of proinflammatory genes through m^6^A modification. (**A** and **B**) Immunoblots showing the expression of critical adapters in the IL-6/ STAT3 signaling pathway in WT and *WTAP*^Δ1–77^ THP-1 cells. (**C**) Flow cytometry showing IL6ST-FITC fluorescence in cell surface of peritoneal macrophages and BMDMs. (**D**) Immunoblots showing the expression of WTAP and IL6ST in the lung or colon tissues from *Wtap*^fl/fl^ and *LyzM*-*cre*^+^ *Wtap*^Δ1–77^ mice that were intraperitoneally injected with LPS for 12 hours. (**E**) Immunoblots showing the levels of phosphorylated STAT3, p65, and p38 in WT and *IL6ST*^–/–^ THP-1 cells stimulated with LPS at the indicated time points. (**F**) ELISAs detecting IL-6 secretion in the supernatants of WT and *IL6ST*^–/–^ THP-1 cells stimulated with indicated inflammatory stimuli. (**G**) Schematic representation showing the position of m^6^A motifs within the IL6ST transcripts. F1/R1 represents detection site 1 and F2/R2 represents detection site 2. (**H**) MeRIP-qPCR showing the abundance of *IL6ST* transcripts in WT and *WTAP*^Δ1–77^ THP-1 cells stimulated with LPS for 6 hours. (**I**) WT or mutant (Mut) m^6^A consensus sequences (**A**–**T** mutation) on the *IL6ST*-3′ UTR were fused with the *Renilla* luciferase reporter in the psiCHECK-2 vector. (**J** and **K**) Relative luciferase activities in 293T cells after transfection with indicated reporter vectors. (**L**) qRT–PCR showing the proportion of *IL6ST* mRNA in polysome fractions from WT and *WTAP*^Δ1–77^ THP-1 cells stimulated with LPS for 6 hours. (**M**) Schematic representation of the biotin-labeled probes of *IL6ST* transcripts. (**N**) RNA pull-down analyses showing the interaction between different *IL6ST* RNA probes and the YTHDF1/3 protein. (**O**) RIP-qPCR showing the interaction between *IL6ST* mRNA and YTHDF1/3 in WT and *WTAP*^Δ1–77^ THP-1 cells. (**P**) Immunoblot analyses showing the abundance of IL18Rα and IL15Rα in WT and *WTAP*^Δ1–77^ THP-1 cells stimulated with LPS at the indicated time points. (**Q**) Relative luciferase activities in 293T cells after transfection with indicated reporter vectors. Data are representative of 3 independent biological experiments in **A**–**E**, **N**, and **P.** Data are presented as the mean ± SD in **F**, **H**, **J**–**L**, **O**, and **Q**, with individual measurements overlaid as dots. Statistical analysis was performed using 1-way ANOVA multiple comparisons in **F**, **H**, **J**, **O**, and **Q**, or 2-tailed Student’s *t* test in **K**.

Based on the sequencing data, we found that many m^6^A-modified genes regulated by WTAP are involved in the transduction of the STAT3 signaling axis, such as IL6ST, TYK2, JAK2, IL15RA, and IL18R1 (Figure 4C and Supplemental Figure 8, A–I). Since the transcription and secretion of IL-6 was significantly reduced in WTAP-deficient cells and its contribution to the activation of STAT3 may be predominant in acute inflammatory response, we then measured the protein abundance of the identified key components involved in the IL-6/STAT3 signaling axis, including IL6ST, IL6R, TYK2, JAK1, JAK2, and STAT3 (36). The results showed that among these key components, only the IL6ST protein abundance was lower in *WTAP*^Δ1–77^ THP-1 cells and *Wtap*^Δ1–77^ BMDMs than in control cells (Figure 5A and Supplemental Figure 9A). Moreover, the upregulation of IL6ST induced by LPS was inhibited in the *WTAP*^Δ1–77^ THP-1 cells (Figure 5B). Consistently, the abundance of IL6ST on the cell surface of *Wtap*^Δ1–77^ macrophages was substantially reduced (Figure 5C). Notably, the analysis of mouse models of LPS-induced sepsis showed that the increased protein expression of IL6ST and WTAP (Supplemental Figure 9, B and C) was reduced in the lung and colon tissues (Figure 5D) from *LyzM*-Cre^+^ *Wtap*^Δ1–77^ mice. We next generated *IL6ST*^–/–^ THP-1 cells using the CRISPR/Cas9 approach, and as shown in *WTAP*^Δ1–77^ THP-1 cells, the level of phosphorylated STAT3 was decreased in *IL6ST*^–/–^ THP-1 cells after stimulation with LPS (Figure 5E). IL-6 expression was also reduced in *IL6ST*^–/–^ THP-1 cells in response to different inflammatory stimuli (Figure 5F).

MeRIP-Seq revealed that the m^6^A peaks obtained with high confidence were distributed in regions near the stop codon in *IL6ST* transcripts (Supplemental Figure 8, A and G). We then designed 2 gene-specific primer pairs, F1/R1 and F2/R2 (Supplemental Table 3), to measure the change in m^6^A abundance at sites 1 and 2 of *IL6ST* transcripts via MeRIP-qPCR assay (Figure 5G). The results showed that the m^6^A marks enriched at both site 1 and 2 in the *IL6ST* transcripts were markedly decreased in *WTAP*^Δ1–77^ THP-1 cells compared with control cells (Figure 5H). For verification, we mutated the putative m^6^A-modified adenosine by replacing it with a thymine in the *IL6ST* mRNA and inserted the WT or mutated (Mut) UTRs into a reporter gene plasmid (psiCHECK-2) (Figure 5I). Luciferase reporter assays showed that the luciferase activity of the Mut reporter was significantly weaker than that of the WT reporter (Figure 5J). Moreover, the luciferase activity of the WT reporter was weaker in *WTAP*^Δ1–77^ THP-1 cells (Figure 5K). Ectopic expression of Flag-tagged WTAP in *WTAP*^Δ1–20^ 293T cells enhanced the luciferase activity of the WT reporter (Supplemental Figure 9D). All these data indicated that the detected m^6^A mark sites in *IL6ST* transcripts are direct substrates of WTAP and are crucial for maintaining the output of the IL6ST protein.

The m^6^A modification can affect many aspects of gene expression, including nuclear export, splicing, 3′-end processing, decay, and translation (37). Because deleting WTAP did not affect the abundance or stability of the *IL6ST* mRNA (Supplemental Figure 9, E and F), we next measured the translation efficiency of IL6ST through polysome profiling (Supplemental Figure 9G). We calculated the proportion of mRNAs in polysome fractions via qRT–PCR and found that the distribution of *IL6ST* mRNA shifted to the lighter fraction in *WTAP*^Δ1–77^ THP-1 cells after stimulation with LPS for 4 hours (Figure 5L). However, this difference was not detected for *GAPDH* mRNA (Supplemental Figure 9H). Taken together, these findings indicated that WTAP can facilitate IL6ST expression by enhancing the translation efficiency via the m^6^A modification. The functional interpretation of m^6^A modification involves RNA-binding proteins called readers, mainly YTHDF1/2/3 and YTHDC1/2, which can influence the degradation or translation of m^6^A-modified RNA (38, 39). Because YTHDF1 and YTHDF3 are the major m^6^A readers that promote the translation of m^6^A-modified mRNAs, we sought to determine whether YTHDF1 or YTHDF3 targets cellular *IL6ST* transcripts to regulate their translation. Immunoblot analyses showed that the deletion of YTHDF1 or YTHDF3 in THP-1 cells using a CRISPR-mediated genome editing approach reduced the expression of IL6ST protein (Supplemental Figure 9I). To verify the direct binding of YTHDF1 and YTHDF3 to m^6^A-methylated *IL6ST* mRNA, we synthesized biotin-labeled RNA probes based on the distribution of the m^6^A peak on *IL6ST* transcripts (Figure 5M) and performed an RNA pull-down assay followed by immunoblotting of the isolated proteins. The results revealed that methylated *IL6ST* transcripts strongly interacted with YTHDF1 and YTHDF3 (Figure 5N). Further, RNA immunoprecipitation (RIP) analyses with antibodies against YTHDF1 or YTHDF3 followed by qRT–PCR revealed that the amount of *IL6ST* mRNA bound to YTHDF1 or YTHDF3 was increased after stimulation with LPS but reduced after WTAP depletion (Figure 5O). These results suggested that the m^6^A marks on *IL6ST* mRNA mediated by WTAP obviously promoted the binding of YTHDF1 and YTHDF3 to *IL6ST* mRNA. Polysome profiling assays showed that the deletion of YTHDF1 or YTHDF3 in THP-1 cells efficiently decreased the expression of IL6ST protein by hindering its translation (Supplemental Figure 9, J and K). Overall, these results indicated that the increased efficiency of IL6ST translation induced by the m^6^A modification is mediated by YTHDF1 and YTHDF3.

In addition to IL-6/IL6ST/STAT3 signaling, the IL-15/IL15R and IL18/IL18R signaling, which dictate T cell response, regulate B cell homing, and activate NK cells (40), may also mediate the regulation of inflammatory responses by activating STAT3 (41, 42). According to our data, the m^6^A peaks distributed in the 3′-UTR regions of *IL15RA* and *IL18R1*, the crucial receptor molecules for IL-15/IL15R and IL18/IL18R signaling, also almost completely disappeared when WTAP was depleted (Supplemental Figure 8, B and C). The abundance of IL15Rα and IL18Rα proteins was reduced in *WTAP*^Δ1–77^ THP-1 cells compared with the control cells (Figure 5P), suggesting that WTAP-mediated m^6^A modification can regulate the expression of IL15Rα and IL18Rα proteins. Further reporter assays performed by replacing the putative m^6^A-modified adenosine with a thymine in the 3′ UTR of *IL15RA* and *IL18R1* mRNAs confirmed that the abundance of *IL15RA* and *IL18R1* proteins was increased by WTAP-mediated m^6^A modification (Figure 5Q and Supplemental Figure 9, L and M). We further rescued the expression of WTAP in *WTAP*^Δ1–77^ THP-1 cells and found that the overall m^6^A modification level increased with the ectopic expression of WTAP (Supplemental Figure 9N). Accordingly, the abundance of IL6ST, IL15Rα, and IL18Rα proteins also increased with increasing m^6^A abundance (Supplemental Figure 9, O and P). All these data revealed that the significance of WTAP in fine-tuning the activation of the IL6/STAT3 signaling axis, implying that the protein synthesis of many proinflammatory genes can be regulated by WTAP via the m^6^A modification in immune homeostasis and disease occurrence, which is unique to WTAP compared with other m^6^A regulators.

### Phase separation of WTAP promotes METTL3 recruitment to efficiently modify inflammatory transcripts.

By verifying the MeRIP-Seq data, we found that the genes affected by WTAP in THP-1 cells did not completely overlap before and after LPS treatment (Supplemental Figure 10A). Transcripts with low m^6^A abundance in *WTAP*^Δ1–77^ THP-1 cells were involved in very few metabolic pathways in the resting state (Supplemental Figure 10B) but were mainly enriched in cytokine production and inflammatory signaling pathways after LPS treatment (Supplemental Figure 10C). Additionally, metagene profiles of the m^6^A peak distribution showed that inflammatory stimulation increased the abundance of m^6^A marks but decreased the number of m^6^A peaks (Supplemental Figure 6, C and D). These observations implied that the upregulation of WTAP activated by p65 may lead to the more concentrated deposition of m^6^A marks on inflammatory genes.

**Figure 6:**
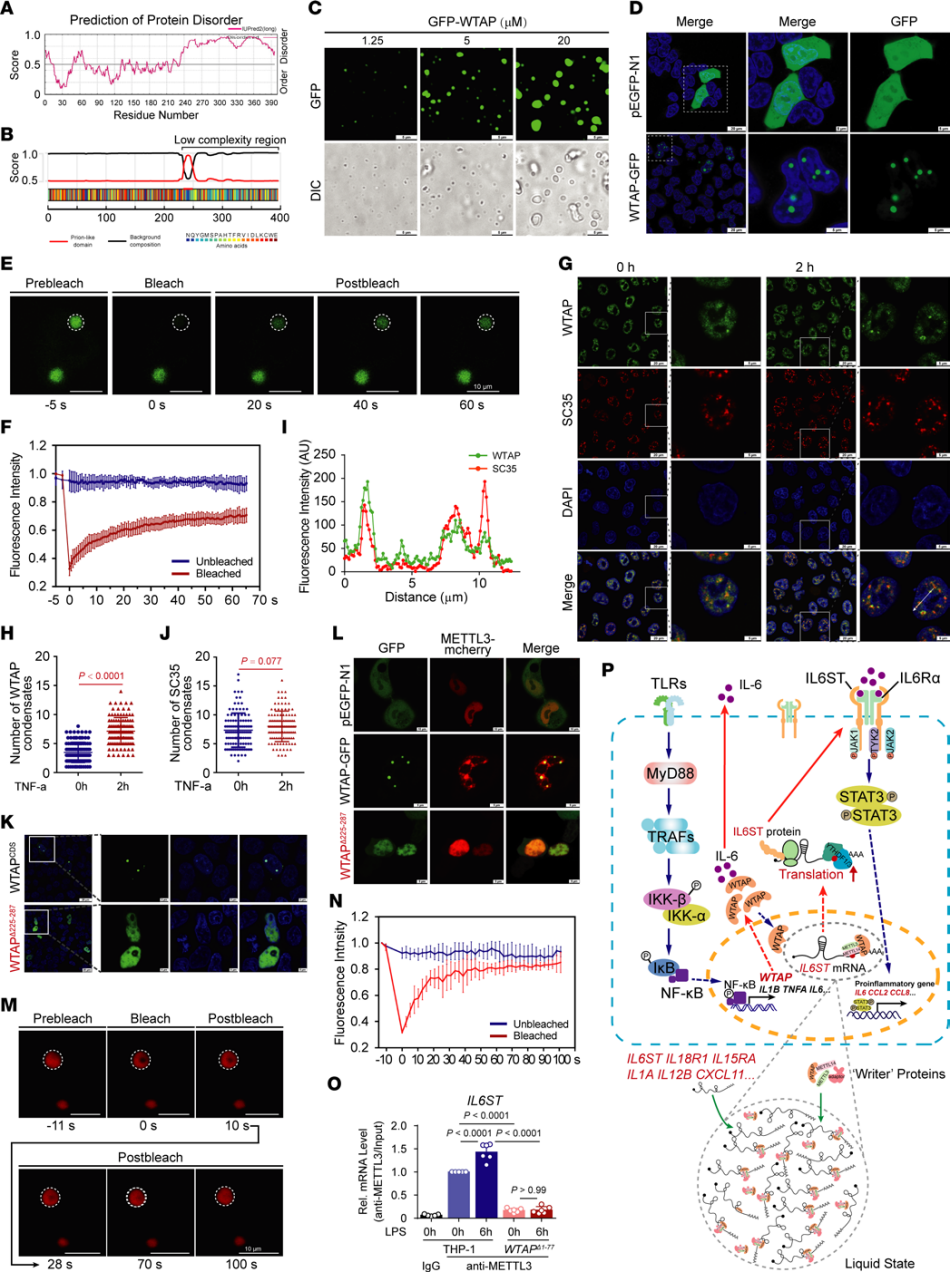
Phase separation of WTAP leads to METTL3 recruitment to efficiently modify inflammatory transcripts. (**A** and **B**) Prediction of the disordered regions and prion-like domains (PrD) of WTAP using the IUPred2A (**A**) or PLAAC (**B**) tool. (**C**) Images of GFP-WTAP droplet formation at room temperature with indicated GFP-WTAP concentrations (350 mM NaCl). Scale bars: 5 μm. (**D**) LSCM images of 293T cells expressing PEGFP-N1 or GFP-tagged WTAP constructs, and dotted box in left-most image is enlarged in the other images. Scale bars: 20 or 5 μm. (**E**) FRAP assays of the GFP-tagged WTAP droplets before and after photobleaching, and the dotted circle highlights the foci undergoing targeted bleaching. Scale bars: 10 μm. (**F**) FRAP quantification of GFP-WTAP droplets over a period of 65 seconds. (**G**) LSCM images of HeLa cells treated with TNF-α for 2 hours, and dotted box in left-most image is enlarged in the other images. Scale bars: 20 or 5 μm. (**H**) Statistics of the number of WTAP droplets in **G**. **(I)** Quantitative line profile of colocalization along a white arrow of the image of **G**. (**J**) Statistics of the number of SC35 droplets in **G**. (**K**) LSCM images of 293T cells expressing full-length or truncated WTAP labeled with GFP. Scale bars: 20 or 5 μm. (**L**) LSCM images of the mCherry-tagged METTL3 droplets (red) in 293T cells before and after cotransfection with GFP-tagged full-length or truncated WTAP. Scale bars: 10 or 5 μm. (**M**)FRAP assays of the mCherry-tagged METTL3 droplets before and after photobleaching from **L**. The dotted circle highlights the foci undergoing targeted bleaching. Scale bars: 10 μm. (**N**) FRAP quantification of mCherry-METTL3 droplets over a period of 100 seconds. (**O**) METTL3 was immunoprecipitated and RIP-qPCR was performed to assess the association of *IL6ST* transcripts with METTL3. (**P**) Working model for WTAP facilitating inflammatory responses through m^6^A modification and phase separation. Data are representative of 3 independent biological experiments in **C**–**E**, **G**, and **K**–**M**. Data are presented as the mean ± SD in **H**, **J** and **O**, with individual measurements overlaid as dots. Statistical analysis was performed using 2-tailed Student’s *t* test in **H** and **J**, or 1-way ANOVA multiple comparisons in **O**. Indicated scale bars are shown in **C**–**E**, **G**, and **K**–**M**.

Recently, liquid–liquid phase separation (LLPS) has emerged as a widespread mechanism through which cells dynamically recruit and organize key signaling molecules. LLPS has also been revealed to play an important role in the formation of the m^6^A writer complex and the regulation of the fate of m^6^A-modified mRNAs (43-46). An analysis of the disordered regions in the WTAP protein using the IUPred2A (https://iupred2a.elte.hu/) and PLAAC (http://plaac.wi.mit.edu/) tools revealed a low-complexity region of 16.5 kDa in the C-terminus of the WTAP coding sequence (CDS) (Figure 6, A and B), which may form liquid droplets as a result of phase separation (47). We further explored whether WTAP can undergo LLPS upon inflammatory stress and how it plays a role in the progression of m^6^A modification of inflammatory transcripts. To this end, recombinantly expressed green fluorescent protein–WTAP (GFP-WTAP) fusion proteins were bacterially expressed and purified (Supplemental Figure 11A). GFP-WTAP formed spherical droplets with an aspect ratio close to 1, and an increase in the protein concentration and salt concentration increased the abundance of GFP droplets from barely detectable small foci to regular and large droplets (Figure 6C and Supplemental Figure 11B). Through time-lapse microscopy, we found that the droplet size of GFP-WTAP increased over time (Supplemental Figure 11C), and we captured the fusion between droplets within 40 seconds (Supplemental Figure 11D), implying the high dynamics and fluidity of the GFP-WTAP droplets. Conversely, 10% 1,6-hexanediol (Hex), a compound that putatively dissolves liquid–liquid phase-separated condensates, substantially inhibited droplet formation (Supplemental Figure 11E).

To further test the ability of WTAP to undergo LLPS in intact cells, we constructed a C-terminal enhanced GFP–tagged (EGFP-tagged) WTAP vector and ectopically expressed it in 293T cells (Supplemental Figure 11F). The results showed that GFP-tagged WTAP formed discrete puncta in the nucleus (Figure 6D). Consistent with the in vitro results, fluorescence recovery after photobleaching (FRAP) assays in 293T cells showed that the fluorescence of GFP-tagged WTAP was efficiently and gradually recovered after bleaching (Figure 6, E and F), indicating the potential phase separation capability of WTAP in vivo. Notably, endogenous WTAP condensates formed in HeLa cells, and the number of these droplets increased significantly after cells were stimulated with TNF-α due to the upregulated expression of WTAP (Figure 6, G and H and Supplemental Figure 11G), suggesting that upregulated WTAP induced by inflammatory stimuli is more prone to phase separation. Consistent with another study, WTAP droplets colocalized with the nuclear speckle marker SC35 (17) (Figure 6, G and I). However, no difference in the number of SC35 droplets was observed before and after inflammatory stimulation (Figure 6J), suggesting that endogenous WTAP droplets formed through self-occurring LLPS rather than the dynamics of the nuclear speckles, and that the LLPS of WTAP may facilitate its colocalization with nuclear speckles.

To further identify the regions in WTAP that are needed for phase separation, we used the PONDR tool (http://www.pondr.com) to predict 5 unfolded intrinsically disordered regions (IDRs) with scores greater than 0.7 in the WTAP protein sequence (Supplemental Figure 11H). A series of truncated WTAP mutants labeled with GFP were subsequently constructed (Supplemental Figure 10H) and expressed in 293T cells (Supplemental Figure 11I). Through confocal imaging, we found that WTAP failed to form droplets only when amino acid residues 225–287 were deleted (Figure 6K and Supplemental Figure 11J), suggesting that this sequence is essential for the LLPS of WTAP. Biomolecular condensates formed through LLPS can maintain locally elevated concentrations of resident proteins and/or RNAs (48). Hence, we subsequently explored the biological function of the phase separation of WTAP. Previous studies revealed that WTAP recruits METTL3 and other cofactors to nuclear speckles and that the RNA-binding capacity of METTL3 is profoundly reduced in the absence of WTAP (17). Here, we found that mCherry-METTL3 (Supplemental Figure 11K) readily fused with phase-separated GFP-WTAP and colocalized to all GFP-WTAP droplets (Supplemental Figure 11L), indicating that fusion with WTAP enhanced METTL3 LLPS in vitro. In vivo, METTL3 also formed liquid droplets with the help of WTAP (Figure 6, L–N), since deleting the LLPS sequence in WTAP disrupted the ability of WTAP to recruit METTL3 through LLPS (Figure 6L) without affecting the direct binding of METTL3 and WTAP (Supplemental Figure 11M), suggesting that WTAP LLPS might prime METTL3 condensation in cells. RIP with antibodies against METTL3 followed by qRT–PCR was performed to confirm that the LLPS of WTAP promotes METTL3 to methylate inflammatory mRNAs, and the results revealed that the amounts of the *IL6ST*, *IL15RA,* and *IL18R1* mRNAs bound by METTL3 were significantly increased after stimulation with LPS and were reduced by WTAP depletion (Figure 6O and Supplemental Figure 11, N and O). These data further indicated that the LLPS of WTAP facilitates the aggregation of METTL3 and the methylation of inflammatory mRNAs. Consistently, the absence of phase separation clearly inhibited the WTAP-dependent m^6^A modification (Supplemental Figure 11P). Taken together, these data suggested that upregulated WTAP undergoes phase separation to facilitate the assembly of the writer complex and the localization of the writer complex to nuclear speckles. Since nuclear speckles are active regions for gene transcription and RNA processing and are rich in transcriptionally active proinflammatory transcripts during inflammatory stress, WTAP-mediated LLPS may improve the accessibility between the m^6^A writer complex and inflammatory transcripts in nuclear speckles, promoting the deposition of m^6^A onto transcriptionally active inflammatory transcripts and the activation of proinflammatory responses (Figure 6P).

### WTAP deficiency alleviates the progression of DSS-induced IBD in mice.

**Figure 7:**
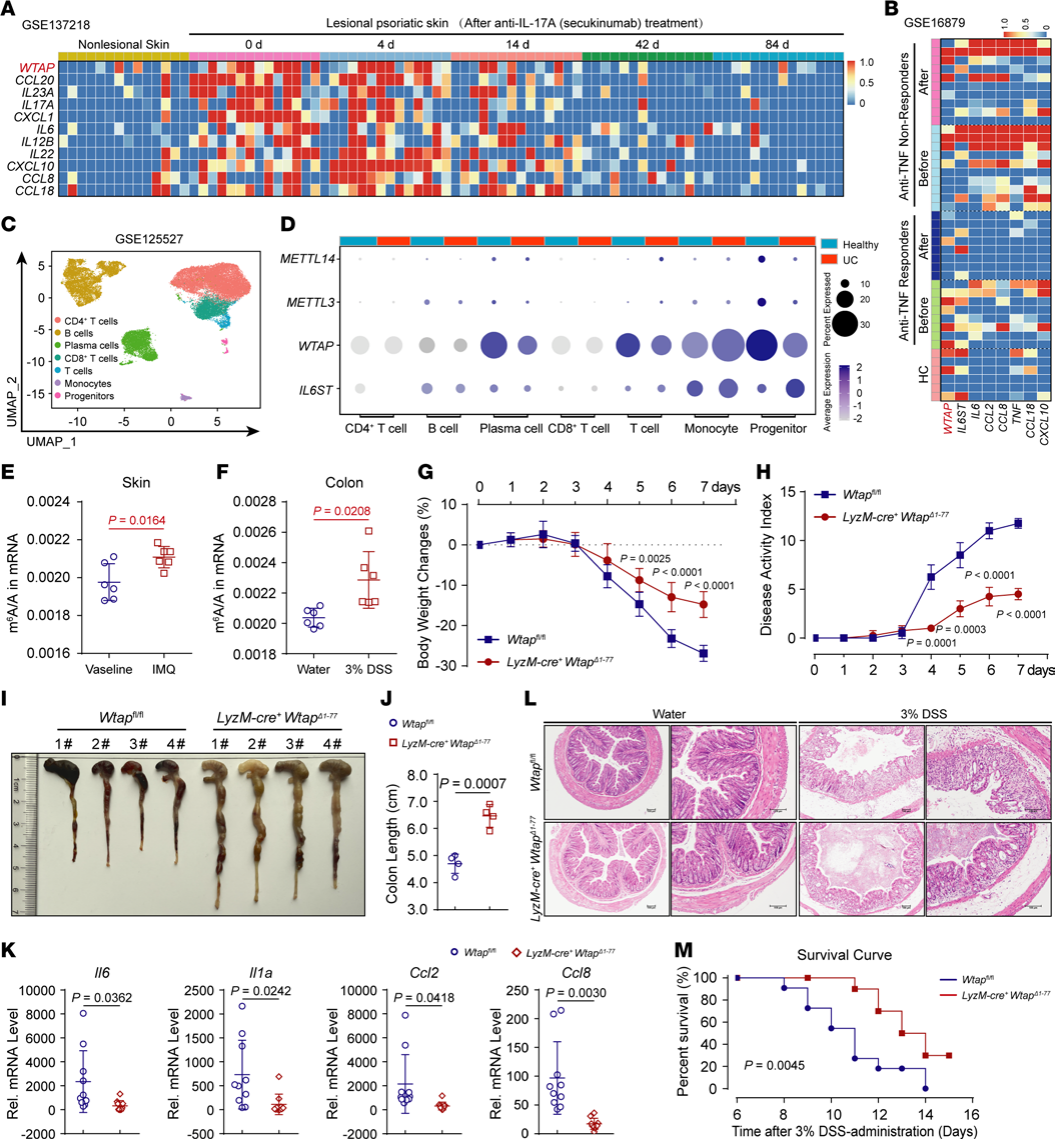
WTAP deficiency alleviates the progression of DSS-induced IBD in mice. (**A** and **B**) Heatmap showing the expression of WTAP and proinflammatory genes in samples from patients with psoriasis before and after anti-IL-17A therapy (**A**) or from patients with CD before and after anti-TNF therapy (**B**). (**C**) Uniform manifold approximation and projection (UMAP) plots showing rectal tissue–derived CD45^+^ immune cells from all participants. (**D**) Dot plots showing the expression of METTL14, METTL3, WTAP, and IL6ST in major immune cell groups. (**E** and **F**) LC-MS/MS quantification of m^6^A abundance in mRNA extracted from skin tissues in IMQ-induced psoriasis (**E**) or colon tissues in DSS-induced colitis (**F**) of mice. (**G** and **H**) Body weight changes (**G**) and disease activity index (**H**) of mice were monitored daily. *n =* 7 mice. (**I** and **J**) Macroscopic appearances (**I**) and colon lengths (**J**) of *Wtap*^fl/fl^ and *LyzM-cre*^+^ *Wtap*^Δ1–77^ mice were recorded on day 6. *n =* 4 mice. Scale bars: 1 cm. (**K**) qRT–PCR showing the mRNA abundance of *Il6*, *Il1*α, *Ccl2,* and *Ccl8* in the colon tissues from *Wtap*^fl/fl^ and *LyzM-cre*^+^ *Wtap*^Δ1–77^ mice that had been given 3% DSS in their drinking water for 6 days. *n =* 10 mice. (**L**) Histopathological changes in colon tissue were determined by H&E staining. Scale bars: 100 μm. *n =* 4 mice. (**M**) The survival of the *Wtap*^fl/fl^ and *LyzM-cre*^+^ *Wtap*^Δ1–77^ mice after continuous feeding with 3% DSS was monitored for 15 days. *n =* 10 mice. Data are representative of 3 independent biological experiments in **I** and **L**). Data are presented as the mean± SD in **E**, **F**, **G**, and **K**, with individual measurements overlaid as dots, statistical analysis was performed using 2-tailed Student’s *t* test. Indicated scale bars are shown in (**I** and **L**).

All of the aforementioned data suggest that WTAP is a true proinflammatory risk factor. We further investigated the potential role and clinical relevance of upregulated WTAP in the progression of inflammatory diseases, and revealed that both WTAP and IL6ST were upregulated in patients with SLE, asthma, sepsis, RA, psoriasis, and IBD (Supplemental Figure 1, A–F and Supplemental Figure 12, A–E). Moreover, high WTAP expression was positively correlated with the level of inflammation in patients. For example, the upregulation of WTAP was reversed in patients with psoriasis treated with anti-IL17A therapy and in patients with Crohn’s disease (CD) treated with anti-TNF therapy (GSE137218/16879; Supplemental Table 1); correspondingly, proinflammatory cytokine expression was also decreased in these patients after treatment (Figure 7, A and B). We further evaluated the expression of WTAP in the progression of IBD. An analysis of a single-cell data set of patients with IBD (GSE125527; Figure 7C and Supplemental Table 1) showed that the expression of WTAP, rather than METTL3, was substantially increased in some immune cell subsets, particularly in monocytes, in patients with IBD (Figure 7D). The prevalence of WTAP upregulation in samples from patients with IBD was then verified using additional public data sets (GSE119600/10616; Supplemental Figure 12, F and G and Supplemental Table 1). Moreover, using data from the IBDMDB database, an analysis of the correlation between genes involved in m^6^A methylation and genes identified as IBD risk genes or biomarkers showed that WTAP exhibited the highest correlation with the expression of IBD genes among all the m^6^A regulators (Supplemental Figure 12H). Increased expression of WTAP was accompanied by an increase in the global m^6^A abundance in patients with various inflammatory diseases (Supplemental Figure 12, I and J), which is consistent with our findings from LPS-induced macrophages, imiquimod-induced (IMQ-induced) psoriasis (Figure 7E) and 3% dextran sodium sulfate–induced (DSS-induced) colitis (Figure 7F) mouse models. Thus, the high expression of WTAP accompanied by a high level of m^6^A modification and high expression levels of proinflammatory cytokines are common characteristics of distinct inflammatory diseases.

To verify the biological correlation between WTAP and IBD signatures, we subjected *LyzM*-Cre^+^ *Wtap*^Δ1–77^ mice and their *Wtap*^fl/fl^ control littermates to DSS to induce acute colitis. The administration of DSS in drinking water can cause the death of intestinal epithelial cells and thus compromise gut barrier function and cause inflammation (49). We found that, compared with their *Wtap*^fl/fl^ control littermates, the *LyzM*-Cre^+^ *Wtap*^Δ1–77^ mice treated with DSS displayed attenuated colitis accompanied by less weight loss (Figure 7G) and a lower disease activity index (Figure 7H). Moreover, WTAP deficiency substantially attenuated the shortening of colon length in DSS-challenged mice (Figure 7, I and J). The low expression of *Il6*, *Il1*α, *Ccl2,* and *Ccl8* in DSS-challenged *LyzM*-Cre^+^ *Wtap*^Δ1–77^ mice indicated attenuated colonic inflammation (Figure 7K). The histopathological assessment revealed that the colonic mucosa of *LyzM*-Cre^+^ *Wtap*^Δ1–77^ mice was relatively intact, with less inflammatory cell infiltration after DSS treatment (Figure 7L). Further, *LyzM*-Cre^+^ *Wtap*^Δ1–77^ mice exhibited a delay in death and a lower death rate (Figure 7M). Taken together, these results suggested that WTAP deficiency attenuates the severity of DSS-induced IBD, revealing a clinically relevant correlation between WTAP levels and IBD progression. Because increased expression of WTAP is ubiquitous in many inflammatory diseases, we hypothesize that WTAP is an important risk factor that exerts a broad spectrum of effects on inflammatory diseases and may serve as a potential therapeutic target for inflammatory diseases.

### Reducing the level of m^6^A modification can reverse the high inflammatory state.

**Figure 8:**
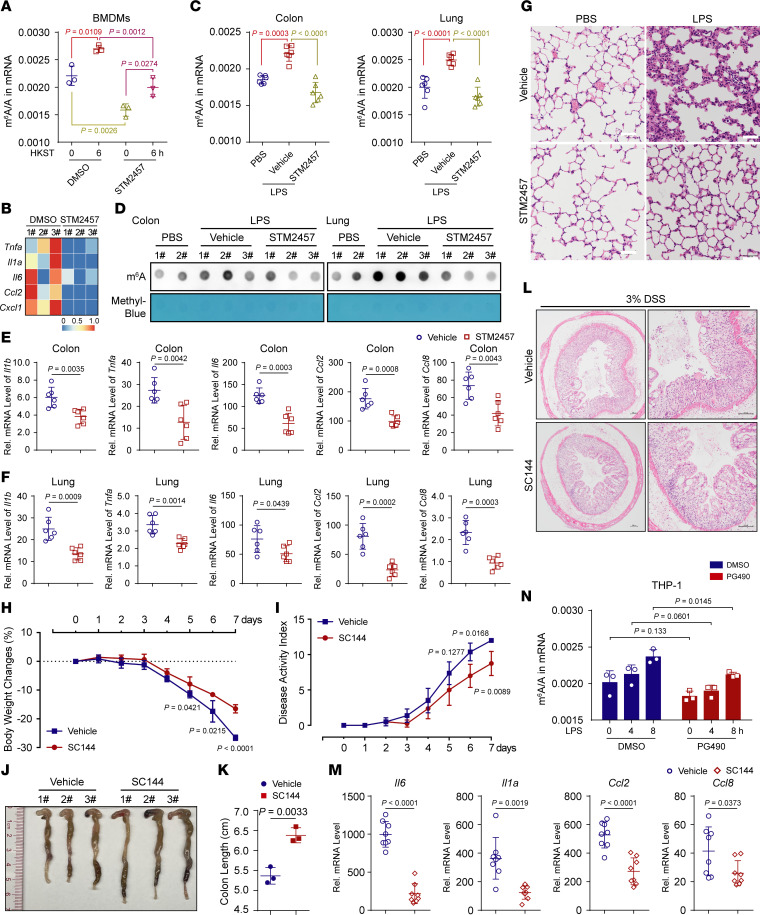
Reducing the level of m^6^A modification can reverse the high inflammatory state. (**A** and **B**) LC–MS/MS quantifying the m^6^A abundance (**A**) and heatmap showing the expression of proinflammatory genes (**B**) in HKST-stimulated BMDMs pretreated with DMSO or STM2457. (**C** and **D**) LC–MS/MS (**C**) and m^6^A dot blot (**D**) quantifying the m^6^A abundance in mRNA extracted from colon or lung tissues in septicemic mice pretreated with vehicle or STM2457. *n =* 3 mice. (**E** and **F**) qRT–PCR showing the mRNA abundance of proinflammatory genes in the colon (**E**) or lung (**F**) tissues from mice treated as above. *n =* 6 mice. (**G**) H&E assays showing the lung injury of LPS-induced sepsis of mice that treated as above. Scale bars: 50 μm. *n =* 3 mice. (**H** and **I**) Body weight changes (**H**) and disease activity index (**I**) of mice were monitored daily. *n =* 4 mice per group. (**J** and **K**) Macroscopic appearances (**J**) and colon lengths (**K**) of mice were recorded on day 8. *n =* 3 mice. (**L**) H&E assays showing the histopathological changes in colon tissue. Scale bars: 100 μm. *n =* 3 mice. (**M**) qRT–PCR showing the mRNA abundance of proinflammatory genes in the colon tissues from IBD mice intraperitoneally injected with vehicle or SC144. *n =* 8 mice. (**N**) LC–MS/MS quantification of m^6^A levels in mRNA extracted from LPS-stimulated THP-1 cells that pretreated with DMSO or PG490. Data are representative of 3 independent biological experiments in **D**, **G**, and **J**. Data are presented as the mean ± SD in **A**, **C**, **E**, **F**, **K**, **M,** and **N**, with individual measurements overlaid as dots. Statistical analysis was performed using 1-way ANOVA multiple comparisons in **A** and **C** or 2-tailed Student’s *t* test in **E**, **F**, **K**, **M**, and **N**.

As we have shown above, hyperinflammatory states are often accompanied by high levels of m^6^A mark, and we further tested whether the METTL3 inhibitor STM2457 could alleviate hyperinflammatory states by reducing the levels of the m^6^A modification. Reducing the abundance of m^6^A marks with STM2457 (Figure 8A) substantially inhibited HKST-induced expression of proinflammatory genes (Figure 8B). We also verified this effect using a mouse model of LPS-induced sepsis by showing that levels of the m^6^A modification were significantly reduced in colon and lung tissues from mice treated with STM2457 (Figure 8, C and D). A decrease in the m^6^A modification level blocked the upregulation of inflammatory cytokines and activation of inflammatory signals induced by LPS (Figure 8, E and F). Similarly, STM2457 effectively alleviated lung inflammation and the pathological characteristics of lung injury (Figure 8G). Thus, reducing global m^6^A levels may be a potential therapeutic strategy for alleviating hyperinflammation.

In addition, the specific IL6ST inhibitor bazedoxifene (BZA) has long been approved by the US FDA as a selective modulator of estrogen receptors to treat osteoporosis (50), suggesting the promise of IL6ST inhibitors as drugs. In this study, we found that WTAP positively regulates proinflammatory response primarily through IL6ST. Therefore, is blocking IL6ST a promising approach for treating inflammatory bowel disease or even other inflammatory diseases in the future? To test this hypothesis, we i.p. injected the vehicle or SC144, along with feeding 3% DSS drinking water, and monitored the disease severity of mice daily. Compared with their control littermates, the mice treated with SC144 displayed attenuated colitis accompanied by less weight loss (Figure 8H) and a lower disease activity index (Figure 8I). Moreover, SC144 treatment attenuated the shortening of colon length in DSS-challenged mice (Figure 8, J and K). The histopathological assessment (Figure 8L) and the low expression of *Il6*, *Il1*α, *Ccl2,* and *Ccl8* in SC144-treated mice indicated attenuated colonic inflammation (Figure 8M). These data suggested that blocking the activation of STAT3 signaling by inhibiting IL6ST can alleviate the progression of DSS-induced IBD in mice. Consistent with this result, a recent study also reported that gp130 (IL6ST) blockade may benefit some patients with Crohn’s disease (51). Hence, our study confirmed that screening suitable inhibitors of IL6ST with few side effects may be a promising approach for treating inflammatory bowel diseases in the future.

## Discussion

### WTAP acts as a smart adapter in the assembly of the m^6^A writer complex to participate in regulating inflammation.

Inflammation is vital for protecting the host against invading pathogens and for repairing tissue damage and requires tight and concise control of pro- and antiinflammatory gene expression. The m^6^A, the most prevalent internal mRNA modification, has been recently linked to various inflammatory states, including autoimmunity, infection, metabolic diseases, and cancers. In this study, we further found that WTAP is a p65-controlled gene that is universally upregulated under distinct inflammatory stimuli. Elevated WTAP protein is strongly related to increased levels of m^6^A modification and excessive inflammatory responses. Notably, the transcription and expression of WTAP have been shown to be regulated in various biological processes. For example, at the transcriptional level, HIF1-α (31) and STAT3 (30) can transactivate WTAP expression by directly binding to its promoter region, and WTAP can also be upregulated by the epigenetic alteration of H3K4me3 (52). At the translational level, pseudogene WTAPP1 can bind to WTAP mRNA to promote its translation by recruiting the EIF3 translation initiation complex (53). At the posttranslational level, our previous study showed that WTAP undergoes degradation via the ubiquitination–proteasome pathway in virus-infected cells, leading to a reduction in the abundance of m^6^A marks and a subsequent attenuation in the intensity of IFN-I signaling (54). Here, we further found that WTAP can also be activated by NF-κB p65 under inflammatory stress. Unlike other m^6^A proteins that regulate inflammatory responses in a context-dependent manner, WTAP is more extensively involved in the establishment of hyperinflammatory states and the occurrence of inflammatory diseases.

In addition to showing that WTAP is a p65-controlled gene, the basic mechanism by which WTAP coordinates the assembly dynamics of the writer complex to promote the m^6^A modification of many inflammatory genes was further revealed in this study. Although WTAP has been shown to be critical for the localization of the m^6^A writer complex to nuclear speckles, its role in the dynamic assembly of the writer complex is not fully explored. A previous study reported that METTL3 can undergo LLPS (46), and METTL3 interacts with m^6^A-METTL-associated complex (MACOM) mainly through WTAP (55). Here, we further revealed that the highly expressed WTAP spontaneously forms condensates through LLPS under inflammatory stress. Moreover, when WTAP is absent or cannot undergo LLPS, METTL3 is dispersed in the nucleus, indicating that the LLPS of WTAP is essential for the assembly of the writer complex and provides dynamic m^6^A regulation under distinct physiological or pathological conditions, which may be important for the recruitment of more writer proteins to nuclear speckles. Because nuclear speckles are sites with many transcriptionally active genes (56), WTAP-mediated LLPS may increase local writer concentrations and improve the accessibility between the m^6^A writer complex and inflammatory transcripts in nuclear speckles, thereby promoting the m^6^A modification of inflammatory genes and accelerating inflammatory response. In the past decade, biomolecular condensates formed by LLPS have been widely reported to modulate many cellular functions by compartmentalizing specific proteins and nucleic acids in subcellular environments with distinct properties (57, 58). Our findings further expand the regulatory roles of phase separation to the dynamic assembly of the writer complex and the methylation of specific transcripts by describing how cells utilize the composition and compartmentation of multivalent condensates to affect the m^6^A epitranscriptome.

### The fine-tuning of the STAT3 signaling axis by targeting WTAP LLPS may be an effective therapeutic strategy for excessive inflammation.

The m^6^A modification has been reported to regulate inflammatory responses and related diseases. Through analyses of clinical data and mouse disease models, we clearly found that high expression of WTAP is associated with inflammatory diseases, such as sepsis, SLE, asthma (59), RA, psoriasis (60), and IBD (61) (Supplemental Figure 1). In addition to revealing the relationship between increased WTAP and increased levels of m^6^A modification in inflammation, we specifically showed that an increase in WTAP protein was accompanied by increases in the abundances of m^6^A on *IL6ST*, *IL15RA*, *IL18R1,* and many other transcriptionally active proinflammatory cytokines. High abundance of IL6ST, IL15RA, and IL18R1 proteins promotes the activation of the STAT3 signaling axis, which in turn contributes to the production of proinflammatory cytokines, such as IL-6, CCL2, and CCL8 (Figure 6P). Because the STAT3 signaling axis has been well characterized as a mechanism that accelerates the production of a variety of cytokines and chemokines (62) and is strongly associated with inflammatory diseases, including cytokine storm syndromes, ADs, and cancers (63, 64), the NF-κB/WTAP/STAT3 axis identified in this study indicated that WTAP is an ideal therapeutic target for the treatment of many inflammatory diseases and cancers. Here, we found that hyperinflammatory states in the body are often accompanied by high levels of m^6^A methylation and that reducing the abundance of m^6^A marks with STM2457 can ameliorate hyperinflammation. So, reversing high m^6^A levels in disease states has broad prospects in the treatment of inflammatory diseases in the future. Because WTAP is more concentrated in the inflammatory context by affecting the m^6^A modification of nascent inflammatory genes, the epigenetic therapies that target WTAP to reprogram the m^6^A landscape in cells may achieve more selective and safer therapeutic outcomes. In addition, we found that the NF-κB p65 inhibitor PG490 markedly reduced the overall m^6^A abundance by inhibiting WTAP expression (Figure 8N). Thus, for the development of novel small antiinflammatory molecules, researchers may need to consider the effects of these inhibitors on the epitranscriptome.

In summary, we provide the first evidence that high levels of m^6^A modification in a variety of hyperinflammatory states depend on NF-κB because the key component of the writer complex, WTAP, is transcriptionally regulated by p65, and its overexpression can lead to increased levels of m^6^A modification. We also discovered that upregulated WTAP undergoes phase separation that facilitates the aggregation of the writer complex and its localization to nuclear speckles, as well as the deposition of m^6^A on transcriptionally active transcripts, resulting in the promotion of proinflammatory responses and the exacerbation of inflammatory diseases. Hence, we hypothesize that the upregulation of WTAP is a risk factor for many hyperinflammatory states and inflammatory diseases. Interrupting the assembly of the m^6^A writer complex by targeting the phase separation of WTAP to reduce the global m^6^A level may be a potential therapeutic strategy for preventing excessive inflammation.

## Methods

### Sex as a biological variable.

Our study examined male and female animals, and similar findings are reported for both sexes.

### Mice.

C57BL/6 *LyzM*-Cre^+^ *Wtap*^Δ1–77^ mice were generated by Gempharmatech Co., Ltd. using the CRISPR/Cas9 approach. All mouse lines were maintained at Sun Yat-sen University under specific pathogen-free (SPF) conditions in ventilated microisolator cages. The IACUC of Sun Yat-sen University (Guangzhou, China) approved all the experimental protocols concerning the handling of mice. The study is compliant with all relevant ethical regulations regarding animals.

### m^6^A RNA-IP-qRT–PCR.

An MeRIP assay was performed following a previously described procedure with minor modifications (54). Briefly, 200 μg of total RNA was fragmented to approximately 150–200 nucleotides in length, purified by magnetic beads, and then incubated with anti-m^6^A antibody- or IgG-conjugated protein A/G magnetic beads in 1 × IP buffer at 4°C overnight. Immunoprecipitated methylated RNAs were eluted by free competitive m^6^A, and recovered with a RNeasy kit (QIAGEN). One-tenth of the fragmented RNA was saved for use as the input control and further analyzed by qRT–PCR with the MeRIP RNAs using primers for the targeted gene. The related enrichment of m^6^A in each sample was calculated by normalizing the number of amplification cycles (Cq) of the m^6^A-IP portion to the Cq of the corresponding input portion.

### MeRIP-Seq and data analysis.

Total RNA was extracted by TRIzol reagent and mRNA was isolated using a Dynabeads mRNA purification kit (Thermo Fisher Scientific). Then, cellar mRNA was fragmented using a fragmentation kit (Thermo Fisher Scientific), and subsequent m^6^A immunoprecipitation, MeRIP-Seq, and data analysis was carried out as previously described with minor modifications (65).

### Supplemental Methods.

Supplemental Methods include information about reagents, generation of knockout cells, in vivo LPS challenge, in vitro recombinant protein expression and purification, ELISA assays, quantitative RT–PCR (qRT–PCR), luciferase reporter gene assay, RNA decay assay, fluorescence recovery after photobleaching (FRAP) assay, laser scanning confocal microscopy (LSCM), live-cell imaging, in vitro phase separation assay, m^6^A dot blots, RNA immunoprecipitation assay, DNA/RNA pull-down assay, quantification of the m^6^A modification by LC-MS/MS, and RNA-Seq and data analysis.

### Statistics.

The data are represented as the mean ± SD unless otherwise indicated, and the sample size for each experiment is indicated in the figure legends. GraphPad Prism software was used to perform statistical analysis. Data were analyzed by 1-way ANOVA with Tukey’s post hoc test for multigroup comparisons or by 2-tailed Student’s *t* test for 2-group comparisons. Survival curves were compared by the log-rank test. Differences between 2 groups were considered statistically significant when the *P* value was less than 0.05.

### Study approval.

All animal experiments were performed in accordance with the NIH Guide for the Care and Use of Laboratory Animals (National Academies Press, 2011), with the approval of the Institutional Animal Care and Use Committee (IACUC) of Sun Yat-sen University (Guangzhou, China).

### Data and code availability.

Publicly available data sets that were analyzed in this study include GSE19315, GSE198326, GSE2411, GSE2638, GSE69063, GSE13887, GSE137268, GSE97779, GSE166388, GSE208303, GSE125527, GSE16879, GSE193193, GSE179874, GSE119600, GSE10616, GSE137218, GSE227851, and GSE189847, and are available at the GEO database (https://www.ncbi.nlm.nih.gov/geo/). The validated IBDMDB data and relevant participants’ information are available at the IBDMDB database (http://ibdmdb.org). All data generated for this paper have been deposited at the SRA under access number: PRJNA943438. Values for all data points in graphs are reported in the Supporting Data Values file. See complete unedited blots in the supplemental material.

## Author contributions

YG, SY, and AX conceived and designed the study, analyzed the data, and prepared the manuscript with input from the other authors. YG, RC, TL, JH, YC, YL, XX, and GX performed the experiments collaboratively. BL, HC, and GL performed the data analysis of MeRIP-seq. SY and AX conceived the study, supervised experiments, and wrote the paper. AX and SY led the project and finally approved the manuscript.

## Supplementary Material

Supplemental data

Unedited blot and gel images

Supporting data values

## Version 1 07/15/2024

Electronic publication
