# Expression of Concern for Elevated WTAP promotes hyperinflammation by increasing m6A modification in inflammatory disease models

**DOI:** 10.1172/JCI213420

**Published:** 2026-10-15

**Authors:** Yong Ge, Rong Chen, Tao Ling, Biaodi Liu, Jingrong Huang, Youxiang Cheng, Yi Lin, Hongxuan Chen, Xiongmei Xie, Guomeng Xia, Guanzheng Luo, Shaochun Yuan, Anlong Xu

Original citation: *J Clin Invest*. 2024;134(14):e177932. https://doi.org/10.1172/JCI177932

Citation for this expression of concern: *J Clin Invest*. 2026;136(20):e213420. https://doi.org/10.1172/JCI213420

The authors recently alerted the Editors to errors in the source data for Figure 3F, Figure 6N, Supplemental Figure 7C, and Supplemental Figure 9J that were identified in response to an institutional mandate for a post-publication audit of the data. Although the authors have indicated that the errors were due to inadvertent mistakes, the Editors are issuing this Expression of Concern to alert readers to the issues. We have requested institutional oversight into this matter and will alert readers to the outcome when the institutional evaluation is complete.

## Version 1 10/15/2026

Electronic publication

