# Supplementary material for: Elevated WTAP promotes hyperinflammation by increasing m6A modification in inflammatory disease models
Source: J Clin Invest. 2024 May 16;134(14):e177932. doi: 10.1172/JCI177932 (PMC11245160; doi:10.1172/JCI177932)
Supplement: Supplemental data [file jci-134-177932-s030.pdf]

Supplemental Materials for

Elevated WTAP promotes hyperinflammation by increasing m^6A modification in inflammatory disease models

Yong Ge, Rong Chen, Tao Ling, Biaodi Liu, Jingrong Huang, Youxiang Cheng, Yi Lin, Hongxuan Chen, Xiongmei Xie, Guomeng Xia, Guanzheng Luo, Shaochun Yuan & Anlong Xu

Correspondence to:

Anlong Xu

lssxal@mail.sysu.edu.cn
Guangzhou 510275, China
P: 086-020-39332990

Or to:

Shaochun Yuan
yuanshch@mail.sysu.edu.cn
Guangzhou 510275, China
P: 086-020-84114373

Supplemental Methods

Reagents. Reagents used are listed in Supplementary Table 4.

Cells. The 293T, HeLa and THP-1 cell lines were purchased from ATCC. All these cells were cultured in endotoxin-free DMEM or RPMI-1640, supplemented with 10% FBS and 1% penicillin–streptomycin. PBMCs and BMDMs were isolated and cultured as described previously (1).

Generation of knockout THP-1 cells. Knockout (KO) cells were constructed using the CRISPR/Cas9 system. Small guide RNAs (gRNAs) targeting the genome sequence of target genes were designed using an online gRNA design tool (by Zhang Feng laboratory, http://crispr.mit.edu/), and subcloned into the lentiCRISPR v2 vector. This vector was transfected into the HEK293T cells along with the following two lentiviral packing plasmids: psPAX2 and pVSV-G. The culture supernatants were collected at 48 and 60 hours after transfection and concentrated by ultracentrifugation before use for infection. The infection-positive (GFP^+) cells were selected by flow cytometry, monoclonal cells were confirmed by genomic sequencing and immunoblotting analysis with the corresponding antibody. The gRNA sequences used for generating the KO cells are listed in Supplementary Table 3.

In vivo LPS challenge. For endotoxicity studies, age-matched *Wtap*$^{fl/fl}$ and *LyzM-cre*$^{+}$ *Wtap*$^{\Delta 1\text{-}77}$ mice (8 weeks old) were intraperitoneally injected with LPS (40 mg kg^{-1}). Mouse survival was monitored every 4 hr.

Wtap$^{fl/fl}$ and *LyzM-cre*$^{+}$ *Wtap*$^{\Delta 1\text{-}77}$ mice (8 weeks old) were intraperitoneally injected with LPS (10 mg kg^{-1}) or isodose PBS. After 12 hr, the mice were killed, blood was collected, and serum levels of IL-6 were measured by ELISA. Lung and colon tissues were collected, and RNA samples were extracted for qRT-PCR to determine the mRNA levels of inflammatory cytokines.

Wtap$^{fl/fl}$ and *LyzM-cre*$^{+}$ *Wtap*$^{\Delta 1\text{-}77}$ mice (8 weeks old) were intraperitoneally injected with LPS (10 mg kg^{-1}) or PBS. After 6 hr, the mice were killed, and lungs from control or LPS-stimulated mice were dissected, fixed in 4% paraformaldehyde (PFA) embedded in paraffin, sectioned, stained with hematoxylin and eosin (H&E) solution, and examined by light microscopy to detect histological changes.

Wtap$^{fl/fl}$ and *LyzM-cre*$^{+}$ *Wtap*$^{\Delta 1\text{-}77}$ mice (8 weeks old) were intraperitoneally injected with *P. aeruginosa* (ATCC27853, 2×10^8 CFU) or *L. monocytogenes* (ATCC19116, 1×10^8 CFU) or PBS. After 16 hr, the mice were killed, and the related phenotypes were measured as described above.

In vitro recombinant protein expression and purification. The expressing plasmids (pET-32a) encoding WTAP conjugated eGFP and METTL3 conjugated mCherry were transformed into BL21 *E.coli*. *E.coli* were cultured in LB with Ampicillin (50 µg/mL) at 37 °C for about 12 hr till OD600 = 0.6. After induction with 1 mM IPTG at 37 °C for 8 hr, the cultured *E.coli* was harvested by centrifugation at 4000 rpm, 4 °C, 10 min and resuspended in lysis buffer. Cells were lysed by sonication on ice and centrifuged (12,000 rpm, 30 min, 4 °C) to remove debris and collected the supernatant. The supernatant was purified by incubation with Ni-NTA agarose beads (QIAGEN) overnight at 4 °C. Then, Ni-NTA beads were washed with wash buffer (50 mM NaH2PO4, 300 mM NaCl, 20 mM imidazole, pH 7.8), and proteins were eluted with elution buffer (50 mM NaH2PO4, 300 mM NaCl, 300 mM imidazole, pH 7.8). The purified proteins were further dialyzed by using PD10 column (GE Healthcare), and concentrated using Amicon Ultra 30 K (Millipore) concentrators at 4 °C. Concentrated proteins were quantified by the BCA method (Thermo Fisher) and stored at -80 °C.

ELISA assays and quantitative RT–PCR (qRT–PCR). The concentrations of IL-6, IL-1β and TNF-α in culture supernatants were measured using kits from R&D Systems or Proteintech, according to the manufacturer's instructions.

Total RNA was extracted using TRIzol reagent (Invitrogen) and reversed-transcribed with a PrimeScript™ RT reagent kit with gDNA Eraser (TaKaRa) according to the manufacturer's instructions. And 2×Polarsignal® qPCR mix (MIKX) was used for quantitative real-time PCR analysis. The data were normalized by the level of *GAPDH* or *ACTB* expression in each individual sample, and primer sequences used are listed in Supplementary Table 3.

Luciferase reporter gene assay. For a promoter reporter gene assay, 293T cells were plated in 48-well plates and transiently transfected with WTAP promoter reporter (pGL3 basic plasmid), with increasing doses of Flag-tagged NF-κB p65, IRF3 or C/EBPβ and 7.5 ng of the *Renilla* luciferase reporter vector using Lipofectamine 2000. At 24 hrs post-transfection, luciferase activities were measured with a dual-luciferase reporter assay system (Promega) according to the manufacturer's instructions. Reporter gene activity was determined by normalization of Firefly luciferase activity to *Renilla* luciferase activity.

For other reporter gene assays, wild-type and $WTAP^{\Delta 1\text{-}20}$ 293T cells or wild-type $WTAP^{\Delta 1\text{-}77}$ THP-1 cells were plated in 48-well plates and transiently transfected with the indicated reporter vectors

(psiCHECK^{TM}-2) expressing wild-type or mutated UTRs of *IL6ST* using Lipofectamine 2000. At 24 hrs post-transfection, the luciferase activities were measured as above. Reporter gene activity was determined by normalization of the *Renilla* luciferase activity to Firefly luciferase activity.

RNA decay assay. *WTAP*$^{\Delta 1\text{-}77}$ and WT THP-1 cells were seeded at a density of 8×10^5 cells/mL in 12-well plates. After treatment with PMA (50 ng/mL, Sigma), the cells were treated with transcription inhibitor Act D (5 μg/mL, Sigma) to block *de novo* RNA synthesis, and collected at different times. RNA samples were extracted for qRT–PCR to determine the mRNA levels of the indicated genes.

Fluorescence recovery after photobleaching (FRAP) assay. FRAP assay was conducted by Leica TCS SP8 STED 3X confocal microscopy. 488- or 568-nm laser beam was used to bleach the fluorescent protein at a region of interest (ROI), followed with collecting time-lapse images. Fluorescence intensity of indicated ROI was measured and normalized to the fluorescence intensity of pre-bleaching image by Leica AS Lite.

Laser scanning confocal microscopy (LSCM). Cells for fluorescence experiments were cultured in glass bottom culture dishes (Nest Scientific). After treated with indicated stimulation, cells were fixed by 4% paraformaldehyde for 10 min, followed with 3 times wash with PBS. Then, cells were permeabilized with methyl alcohol for 30 min at −20 °C and rinsed with PBS for 3 times. After blocking in 5% goat serum for 1 h at room temperature, cells were incubated with primary antibodies at 4 °C, overnight. 1 × PBST (PBS with 0.1% Tween20) was used to wash the cells for 3 times and subsequently incubated with fluorescently labeled secondary antibodies at room temperature for 1 h. Confocal images were obtained by Leica TCS SP8 STED 3X confocal microscope. The number of condensates were counted and analyzed by ImageJ software.

Live-cell imaging. At 24 hrs post-transfection incubation, cells were loaded into temperature- and CO2-controlled live-cell imaging chamber of Leica TCS SP8 STED 3X confocal microscope. Cells were imaged typically by use of 2 laser wavelengths (488 nm for eGFP imaging and 560 nm for mCherry imaging).

In vitro phase separation assay. The purified recombinant proteins were mixed at indicated concentration with LLPS buffer and 5% PEG8000 at 37 °C. The mixture was pipetted onto glass bottom dish and imaged by Leica TCS SP8 STED 3X confocal microscope equipped with 100 × 1.40 NA oil objectives.

m^6A dot blots. Total RNA was extracted with TRIzol reagent. Equal amounts of RNA (300 ng) were denatured at 95 °C for 3 min. The samples were immediately chilled on ice, added to a positively charged nylon membrane (PALL), and then cross-linked with a UV crosslinker. After blocking and incubating with the m^6A antibody (CST) overnight, the membrane was incubated with the secondary antibody at room temperature for 1 hr. Signals were detected using a chemiluminescence imaging system. Methylene blue in 0.3 M sodium acetate (pH 5.2) was used to indicate the amount of total RNA.

RNA immunoprecipitation assay (RIP). RIP was conducted using a RIP assay Kit (MBL) following the manufacturer's instructions. In brief, Protein A/G magnetic beads coated with 5 μg of specific antibody or normal IgG were incubated with cell lysates at 4 °C overnight. Proteins were then extracted for immunoblot analysis, and the co-precipitated RNAs were isolated by elution buffer and purified by TRIzol reagent, and subsequently subjected to qRT–PCR analysis.

DNA/RNA pull-down assay. Biotin-labeled DNA and RNA probes were synthesized by RiboBio. For DNA pull-down assay, Flag-tagged NF-κB p65 expression constructs were transfected into 293T cells. At 24 hrs post-transfection, whole cell lysates were extracted from 293T cells using IP lysis buffer. Biotin-coupled DNA-protein complex was pulled down by incubating whole cell lysates with high-capacity streptavidin agarose beads (Thermo Fisher) according to the manufacturer's instructions. The bound proteins were eluted and used for immunoblot analysis.

For RNA pull-down assay, biotin-coupled RNA complex was pulled down by incubating cell lysates with high-capacity streptavidin agarose beads, bound proteins were then extracted for immunoblot analysis.

Quantification of the m^6A modification by LC-MS/MS. The 200ng extracted mRNA was digested into nucleosides by Nuclease P1 (1 U, NEB, M0660S) and shrimp alkaline phosphatase (rSAP, 1 U, NEB, M0371S) in 50 μL RNase-free water at 37 °C overnight. The mixture was diluted to 100 μL, 10 μL of which was injected into an LC–MS/MS system consisting of a high-performance liquid chromatographer (Shimadzu) equipped with a C18-T column (Weltech) and a Triple Quad 4500 (AB SCIEX) mass spectrometer in positive ion mode by multiple-reaction monitoring. Mass transitions of m/z 268.0–136.0 (A), m/z 245.0–113.1 (U), m/z 244.0–112.1 (C), m/z 284.0–152.0 (G) and m/z 282.0–150.1 (m^6A) were monitored. A concentration series of pure commercial nucleosides (MCE) was employed to generate standard curves. The concentration of nucleosides in

samples were obtained by fitting signal intensity to a standard curve with certain ratios calculated subsequently.

RNA-seq and data analysis. Whole-cell total RNA was isolated using TRIzol reagent and quantified using a NanoDrop 2000 spectrophotometer (Thermo). The cDNA library was constructed by Biomarker Technologies. Sequencing was performed on an Illumina HiSeq 2500 platform. High-quality reads were mapped to the human reference genome (hg19) or mouse reference genome (mm9) using HISAT2. DESeq, an R package, was applied for differential gene expression analysis. We filtered the differentially expressed genes based on a false discovery rate (FDR) <0.05.

Supplemental data

Characterizations of WTAP-deficient cells

To determine the function of WTAP in inflammatory responses, we first generated *WTAP*-knockout THP-1 cells using the CRISPR/Cas9 approach. Two sgRNAs were designed to target the second and third exons (E2 and E3) of *WTAP*. sgRNA#1 caused a two-base insertion, whereas sgRNA#2 caused a one-base deletion in the *WTAP* genomic locus (Supplementary Figure 3, A and B), leading to the introduction of a new stop codon upstream of the CDS and resulting in the early termination of WTAP translation. However, based on the predicted open reading frame (ORF), cells may skip these mutations by initiating translation at an alternative site to produce alternative WTAP isoforms of 376 and 319 aa, respectively (Supplementary Figure 3C). A subsequent immunoblotting assay using a monoclonal antibody (mAb) purchased from Abcam confirmed the extremely low expression of these two isoforms (Supplementary Figure 3D). Thus, the cell lines were designated "$WTAP^{\Delta 1\text{-}20}$ THP-1 cells" and "$WTAP^{\Delta 1\text{-}77}$ THP-1 cells". In addition to generating WTAP-deficient THP-1 cells, we also used the same sgRNAs to knock out WTAP in 293T cells and successfully obtained clones using sgRNA#1. Genomic sequencing confirmed that sgRNA#1 targeted the second exon and introduced a four-base deletion, also resulting in the early termination of WTAP translation. However, similar to what occurred in $WTAP^{\Delta 1\text{-}20}$ THP-1 cells, the truncated $\mathrm{WTAP}^{\Delta 1\text{-}20}$ isoform may be produced and was rarely expressed in 293T knockout cells (Supplementary Figure 3, E-G); thus, the cells were designated "$WTAP^{\Delta 1\text{-}20}$ 293T cells".

Characterizations of WTAP conditional KO mice

We next generated the *Wtap* conditional KO (CKO) mice by crossing $Wtap^{\mathrm{flox/flox}}$ mice with mice expressing Cre recombinase under the control of the lysozyme 2 promoter (*LyzM*-Cre) (Supplementary Figure 3H). Genomic sequencing confirmed that the third exon was fully removed after crossing, which also resulted in the early termination of WTAP translation (Supplementary Figure 3, I and J). Similarly, the ORF prediction revealed that gene-edited BMDMs may also produce alternative WTAP isoforms with 319 aa (Supplementary Figure 3J). However, none of the purchased antibodies targeting WTAP could identify this predicted isoform (Supplementary Figure 3, K and L). Although commercially available antibodies for detection are not sufficient, the expression of $\mathrm{WTAP}^{\Delta 1\text{-}77}$ should be extremely low in BMDMs if it is present, as we have previously observed in THP-1 cells.

Functional deficiency of truncated WTAP$^{\Delta1-20}$ and WTAP$^{\Delta1-77}$

WTAP possesses a nuclear localization signal (NLS) at its N-terminus (Supplementary Figure 3C), and mutation of this signal can affect the entry of the protein into the nucleus and its function (2). Since the NLS is missing in both alternative isoforms, the entry of these isoforms into to the nucleus may be affected. Nuclear–cytoplasmic extraction assays confirmed that WTAP$^{\Delta1-20}$ and WTAP$^{\Delta1-77}$ were more concentrated in the cytoplasm (Supplementary Figure 3, M and N). Moreover, LC–MS/MS assays indicated that ectopic expression of full-length WTAP but not the two alternative isoforms in *WTAP*$^{\Delta1-20}$ 293T cells could increase the global m^{6}A modification level (Supplementary Figure 3O). Similarly, the m^{6}A modification levels in *WTAP*$^{\Delta1-20}$ and *WTAP*$^{\Delta1-77}$ THP-1 cells were lower than those in wild-type cells (Supplementary Figure 3P), and the same trend was observed in BMDMs from *LyzM*-Cre$^+$ *Wtap*$^{\Delta1-77}$ mice (Supplementary Figure 3Q). Due to the extremely low expression and the severely impaired function of WTAP$^{\Delta1-20}$ and WTAP$^{\Delta1-77}$, the status of *WTAP*$^{\Delta1-20}$ and *WTAP*$^{\Delta1-77}$ cells should be very close to that of cells in which the protein is completely knocked out. Due to the extremely low expression and severely impaired function of WTAP$^{\Delta1-20}$ and WTAP$^{\Delta1-77}$, the status of *WTAP*$^{\Delta1-20}$ and *WTAP*$^{\Delta1-77}$ cells should be very similar to that of cells in which the protein is completely deleted.

Additionally, no differences in the proportions of major immune cell populations were observed between *Wtap*$^{fl/fl}$ and *LyzM*-Cre$^+$ *Wtap*$^{\Delta1-77}$ mice in the steady state (Supplementary Figure 4, A-C), indicating that the depletion of *Wtap* in myeloid cells did not affect macrophage development or maturation.

References

1. Ge Y, et al. Degradation of WTAP blocks antiviral responses by reducing the m^{6}A levels of IRF3 and IFNAR1 mRNA. *EMBO Rep*. 2021;22(11):e52101.
2. Scholler E, et al. Interactions, localization, and phosphorylation of the m^{6}A generating METTL3-METTL14-WTAP complex. *RNA*. 2018;24(4):499-512.

Supplemental figures and figure legends

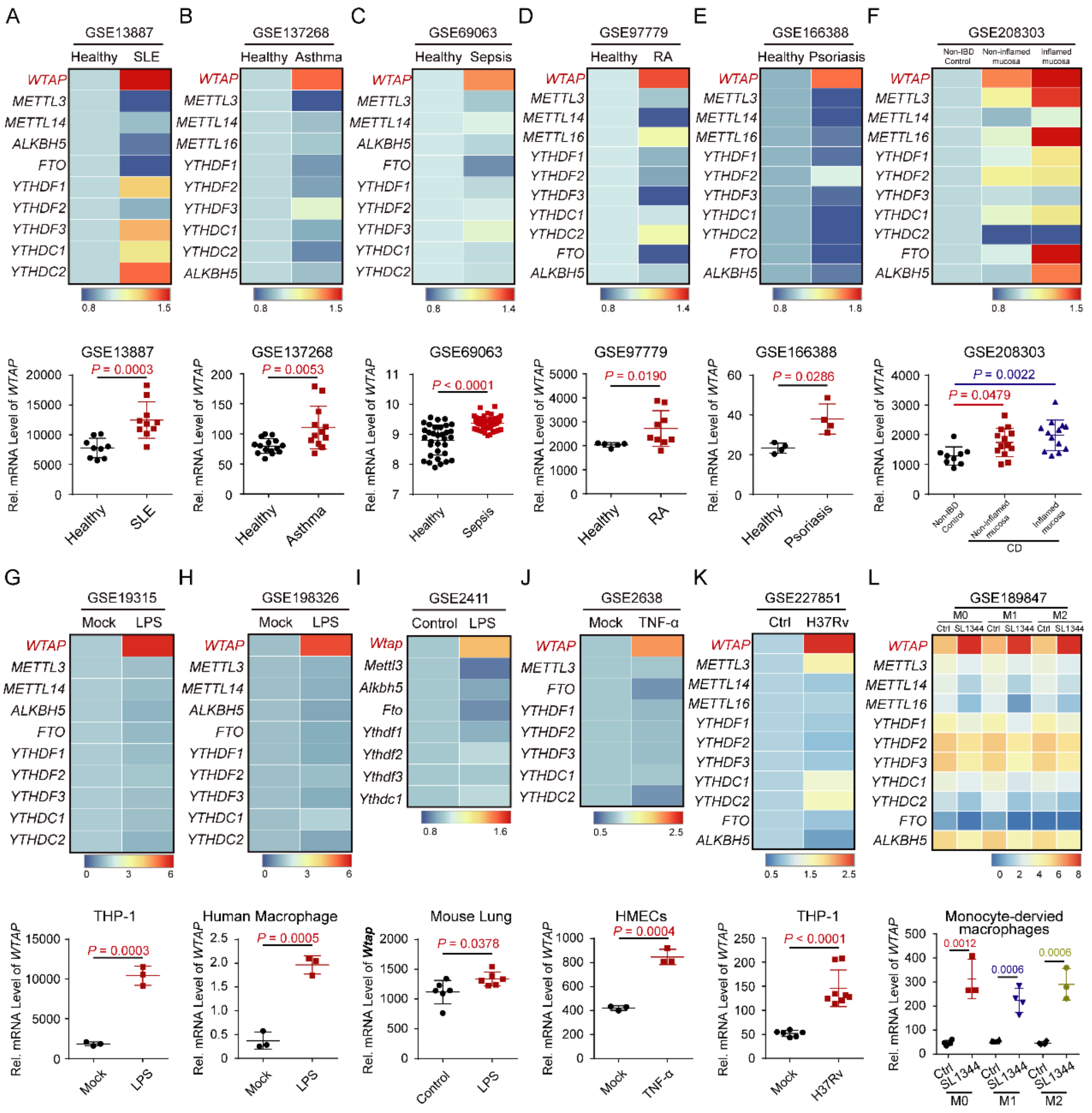

Supplemental Figure 1. Hyperinflammation is accompanied by elevated WTAP level in many inflammatory diseases. (**A** to **F**) Heatmap showing the mRNA abundance of m^{6}A-related genes in patients with SLE (A), asthma (B), sepsis (C), RA (D), psoriasis (E) and IBD (F) compared with healthy controls. **(G** to **L)** Heatmap showing the mRNA abundance of m^{6}A-related genes in THP-1 cells (G) and human macrophages (H) stimulated with LPS, in lung tissues of mice with LPS-induced lung injury (I), in TNF-α-stimulated HMECs (J), in *Mycobacterium tuberculosis*-infected THP-1 cells (K) or in *S. typhimurium*-infected macrophages (L). The relative mRNA expression of *WTAP* is shown below.

Data are presented as the mean ± s.d. in (A to I) with individual measurements overlaid as dots. Statistical analysis was performed using Mann–Whitney U test in (A to E), Kruskal–Wallis test in

(F) or two-tailed Student's *t*-test in (G to L).

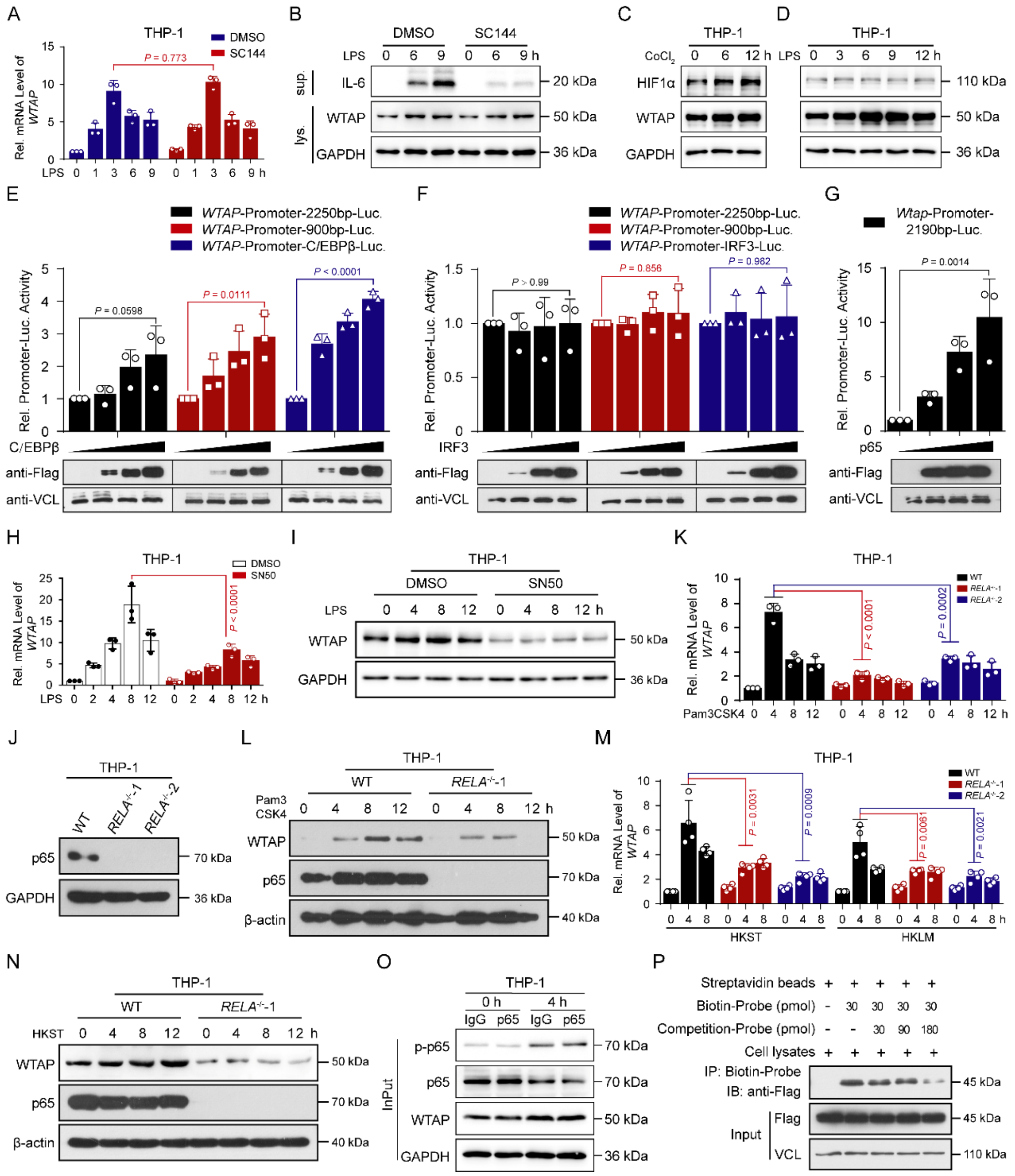

Supplemental Figure 2. The upregulated WTAP in hyperinflammation is controlled by NF-κB p65. (**A** and **B**) qRT-PCR (A) or immunoblots (B) showing the expression of WTAP in THP-1 cells pretreated with SC144, followed by stimulation with LPS at the indicated time points. (**C**) Immunoblots showing the expression of WTAP and HIF1α in THP-1 cells treated with $CoCl_2$. (**D**) Immunoblots showing the expression of WTAP and HIF1α in THP-1 cells stimulated with LPS at different time points. (**E** and **F**) Luciferase activity analyses in 293T cells transfected with a luciferase reporter for the *WTAP* promoter, together with increasing doses of Flag-tagged C/EBPβ (E) or IRF3 (F). (**G**) Luciferase activity analyses in 293T cells transfected with a luciferase reporter for mouse *Wtap* promoter, together with increasing doses of Flag-tagged NF-κB p65. (**H** and **I**) qRT-

PCR or immunoblots showing the expression of WTAP in THP-1 cells pretreated with SN50, followed by stimulation with LPS at the indicated time points. **(J)** Immunoblots showing the efficiency of p65 knockout in THP-1 cells. (**K-N**) qRT-PCR (K and M) or immunoblots (L and N) showing the expression of WTAP in WT and *RELA*$^{-/-}$ THP-1 cells that were stimulated with Pam3CSK4, HKST or HKLM at the indicated time points. (**O**) Immunoblots showing the expression of WTAP and (p-) p65 in THP-1 cells stimulated with LPS at different time points. **(P)** DNA pull-down assays showing the effect of C/EBPβ binding with *WTAP* promoter probes.

Data are presented as the mean ± s.d. in (A), (E to H), (K) and (M) with individual measurements overlaid as dots. Statistical analysis was performed using two-tailed Student's *t*-test in (A) and (E to H), or one-way ANOVA multiple comparisons in (K) and (M). Data are representative of three independent biological experiments in (B to G), (I), (J), (L) and (N-P).

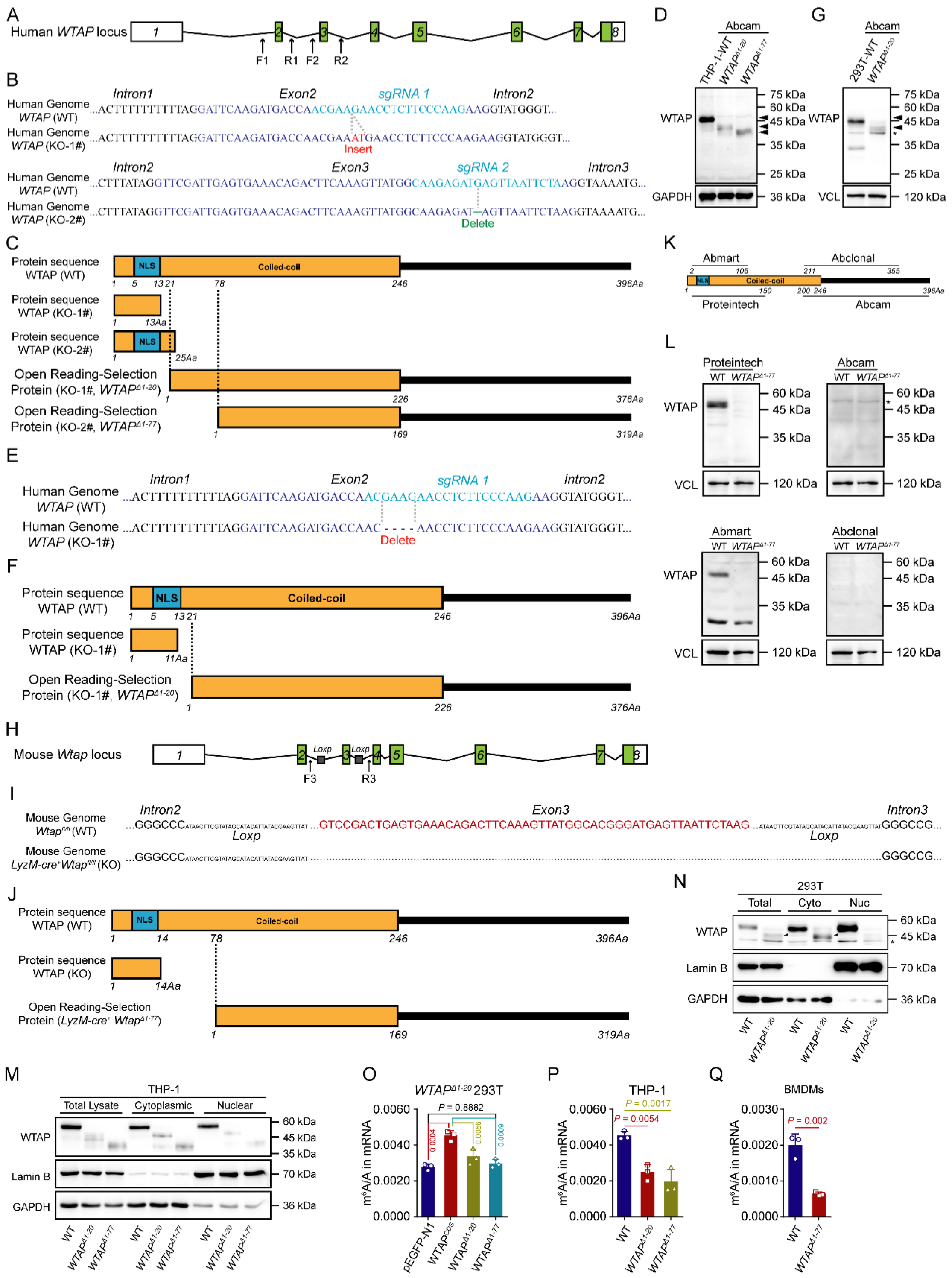

Supplemental Figure 3. WTAP-deficient cells express shorter but dysfunctional isoforms of WTAP. (**A**) Schematic representation of the construction of *WTAP* KO THP-1 cells. (**B**) Genotypes of sequenced THP-1 cells. PCR products were cloned and sequenced. (**C**) Open reading frame prediction**.** (**D**) Immunoblots showing the expression of WTAP in WT and KO THP-1 cells. (**E**) Genotypes of sequenced 293T cells. PCR products were cloned and sequenced. (**F**) Open reading frame prediction**.** (**G**) Immunoblots showing the expression of WTAP in WT and KO 293T cells.

(**H**) Schematic showing the *Wtap* conditional KO (cKO) strategy using the Cre-loxP system in mouse myeloid cells. (**I**) Genotypes of sequenced cells. PCR products were cloned and sequenced. (**J**) Open reading frame prediction**.** (**K**) Immunogen information for different brands of WTAP antibodies. (**L**) Immunoblots showing the expression of WTAP in WT and KO BMDMs. (**M** and **N**) Immunoblots showing the subcellular localization of wild-type and alternative WTAP proteins in WT, $WTAP^{\Delta 1\text{-}20}$ and $WTAP^{\Delta 1\text{-}77}$ THP-1 cells or in WT and $WTAP^{\Delta 1\text{-}20}$ 293T cells. (**O**) LC–MS/MS detection the m^{6}A abundance in mRNA extracted from $WTAP^{\Delta 1\text{-}20}$ 293T cells transfected with PEGFP-N1, GFP-tagged WTAP, GFP-tagged $\mathrm{WTAP}^{\Delta 1\text{-}20}$ or GFP-tagged $\mathrm{WTAP}^{\Delta 1\text{-}77}$. (**P**) LC–MS/MS detection the m^{6}A abundance in mRNA extracted from WT, $WTAP^{\Delta 1\text{-}20}$ and $WTAP^{\Delta 1\text{-}77}$ THP-1 cells. (**Q**) LC–MS/MS detection the m^{6}A abundance in mRNA extracted from WT and $Wtap^{\Delta 1\text{-}77}$ BMDMs.

Data are representative of three independent biological experiments in (D), (G) and (L-N). Data are presented as the mean ± s.d. in (O to Q), with individual measurements overlaid as dots. Statistical analysis was performed using one-way ANOVA multiple comparisons in (O) and (P), or two-tailed Student's *t*-test in (Q).

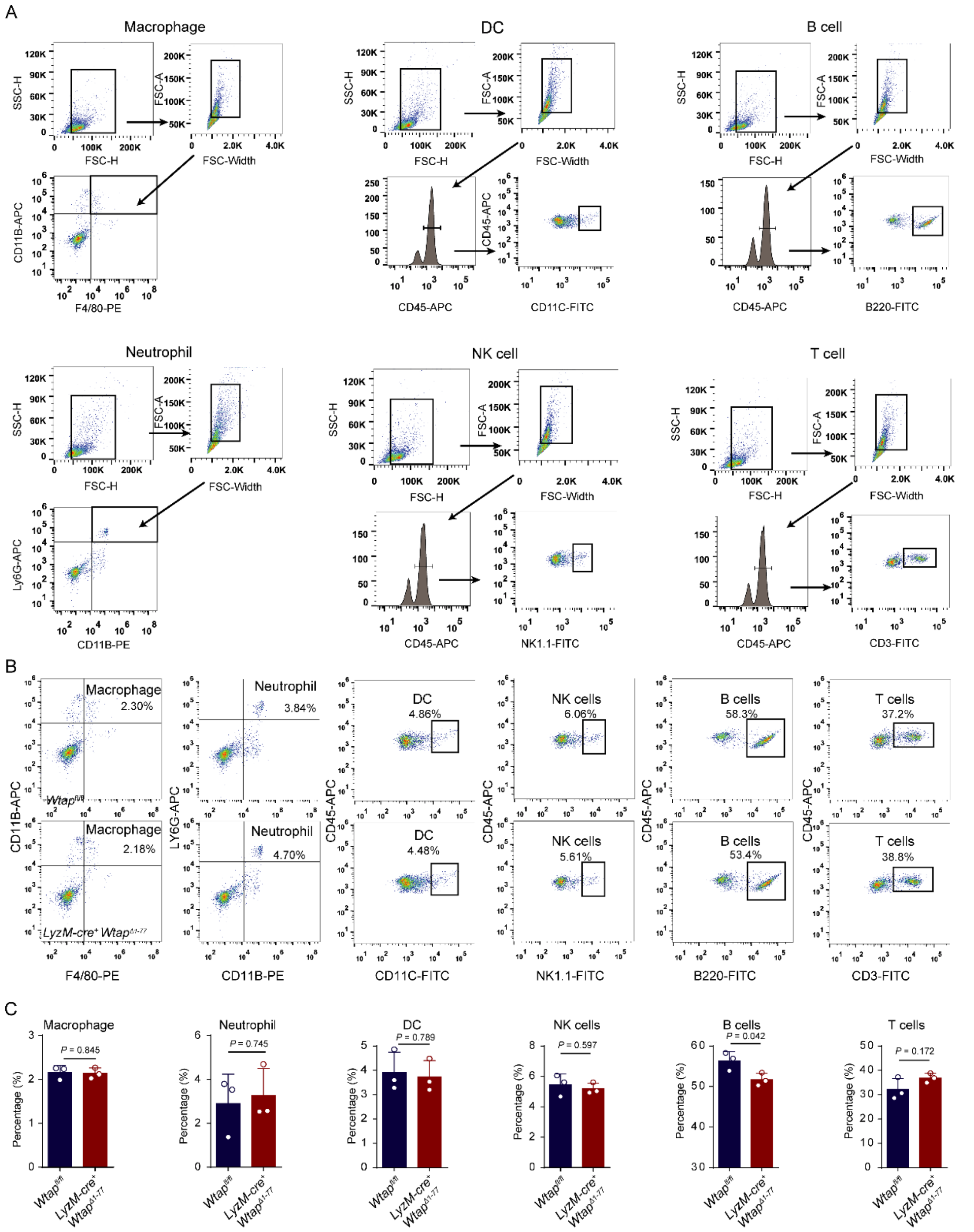

Supplemental Figure 4. *Wtap* deficiency in myeloid cells did not affect the development or maturation of major immune cell populations. (**A**) The gating strategy for sorting out various immune cells. (**B**) FACS analyses of major immune cell populations in spleens from *Wtap*$^{fl/fl}$ mice and *LyzM*-Cre^{+} *Wtap*$^{\Delta 1\text{-}77}$ mice. n = 3 mice per group. (**C**) Percentage of macrophages, neutrophils, DC, NK, T and B cells from (B). n = 3 mice per group.

Data are representative of three independent biological experiments in (B). Data are presented as the mean ± s.d. in (C), with individual measurements overlaid as dots, statistical analysis was

performed using two-tailed Student's *t*-test.

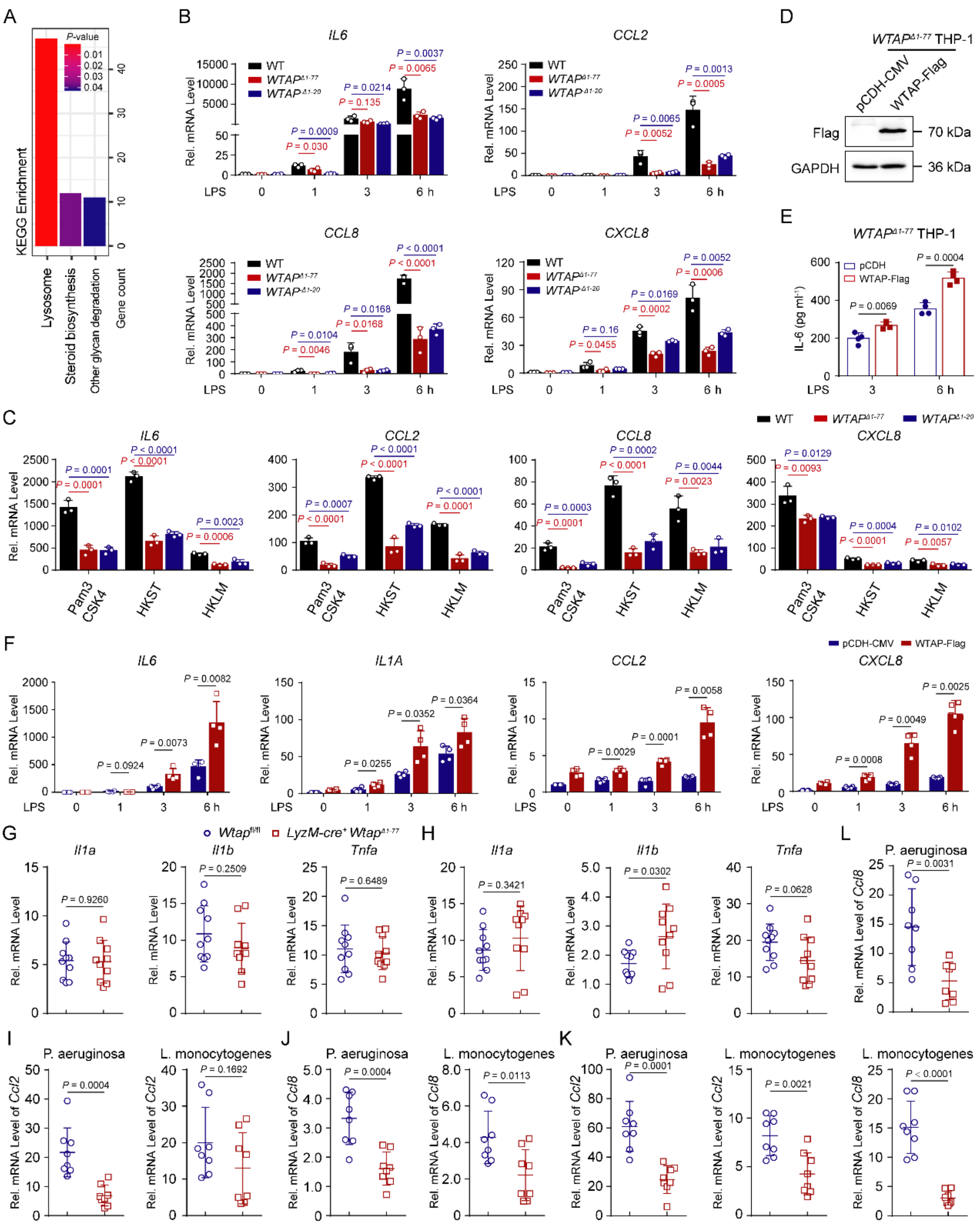

Supplemental Figure 5. WTAP positively regulates proinflammatory responses. (A) KEGG analyses of up-regulated DEGs in *WTAP*$^{\Delta 1\text{-}77}$ THP-1 cells stimulated with LPS compared with WT cells. (**B**) qRT–PCR showing the mRNA abundance of *IL6*, *CCL2*, *CCL8* and *CXCL8* in WT and *WTAP*$^{\Delta 1\text{-}77}$ THP-1 cells stimulated with LPS at the indicated time points. **(C)** qRT–PCR showing the mRNA abundance of *IL6*, *CCL2*, *CCL8* and *CXCL8* in WT and *WTAP*$^{\Delta 1\text{-}77}$ THP-1 cells that were stimulated with Pam3CSK4, HKST or HKLM at 6 hr. **(D)** Immunoblots showing the ectopic expression of Flag-tagged WTAP in *WTAP*$^{\Delta 1\text{-}77}$ THP-1 cells. **(E)** ELISAs were performed to detect

IL-6 secretion in supernatants of *WTAP*$^{Δ1\text{-}77}$ THP-1 cells transfected with empty vector or Flag-tagged WTAP, followed by stimulation with LPS at 3 and 6 hr. **(F)** qRT–PCR showing the mRNA abundance of *IL6*, *IL1A*, *CCL2* and *CXCL8* in *WTAP*$^{Δ1\text{-}77}$ THP-1 cells transfected with Flag-tagged WTAP, followed by stimulation with LPS at different time points. (**G** and **H**) qRT–PCR showing the mRNA abundance of *Il1a*, *Il1b* and *Tnfa* in the lung (G) or colon (H) tissues from *Wtap*$^{fl/fl}$ and *LyzM-cre*$^{+}$ *Wtap*$^{Δ1\text{-}77}$ mice that were intraperitoneally injected with LPS (10 mg kg^{-1}) for 12 hr. n = 10 mice per group. (**I** to **L**) qRT–PCR showing the mRNA abundance of *Ccl2* and *Ccl8* in the colon tissues from *Wtap*$^{fl/fl}$ and *LyzM-cre*$^{+}$ *Wtap*$^{Δ1\text{-}77}$ mice that were intraperitoneally injected with *P. aeruginosa* (I and J) or *L. monocytogenes* (K and L) for 16 hr. n = 8 mice per group.

Data are presented as the mean ± s.d. in (B), (C) and (E to L), with individual measurements overlaid as dots. Statistical analysis was performed using one-way ANOVA multiple comparisons in (B) and (C), or two-tailed Student's *t*-test in (E to L). Data are representative of three independent biological experiments in (D).

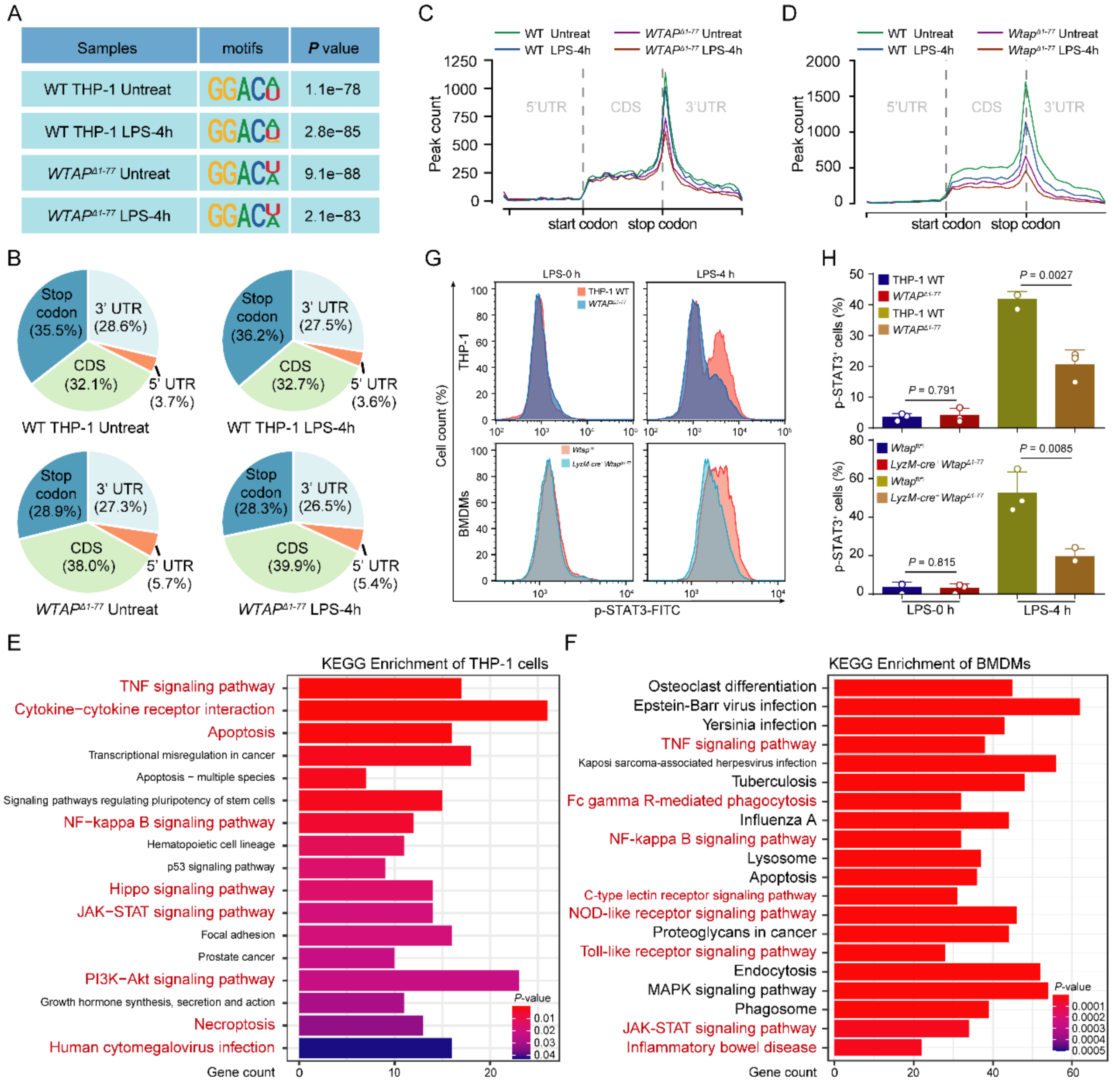

Supplemental Figure 6. WTAP deficiency significantly reduces the abundance of m^{6}A modification of inflammatory genes. (**A**) Sequence motif was identified within m^{6}A peaks by HOMER analyses of WT and *WTAP*$^{\Delta1\text{-}77}$ THP-1 cells with or without LPS treatment. (**B**) Pie charts depicting the proportion of m^{6}A peak distribution in the 5' UTR, CDS, and 3' UTR regions across mRNA transcriptome. (**C** and **D**) Metagene profiles of m^{6}A peak distribution across the 5' UTR, CDS, and 3' UTR in WT and *WTAP*$^{\Delta1\text{-}77}$ THP-1 cells (C) or *Wtap*$^{\Delta1\text{-}77}$ BMDMs (D) with or without LPS treatment. (**E** and **F**) KEGG enrichment analysis of the transcripts with decreased m^{6}A marks in *WTAP*$^{\Delta1\text{-}77}$ THP-1 cells (E) or *Wtap*$^{\Delta1\text{-}77}$ BMDMs (F) after treatment with LPS. (**G** and **H**) Representative flow cytometry data showing p-STAT3 fluorescence intensity in WT and *WTAP*$^{\Delta1\text{-}77}$ THP-1 cells or BMDMs before and after LPS stimulation.

Data are presented as the mean ± s.d. in (H), with individual measurements overlaid as dots, statistical analysis was performed using two-tailed Student's *t*-test. Data are representative of three

independent biological experiments in (G).

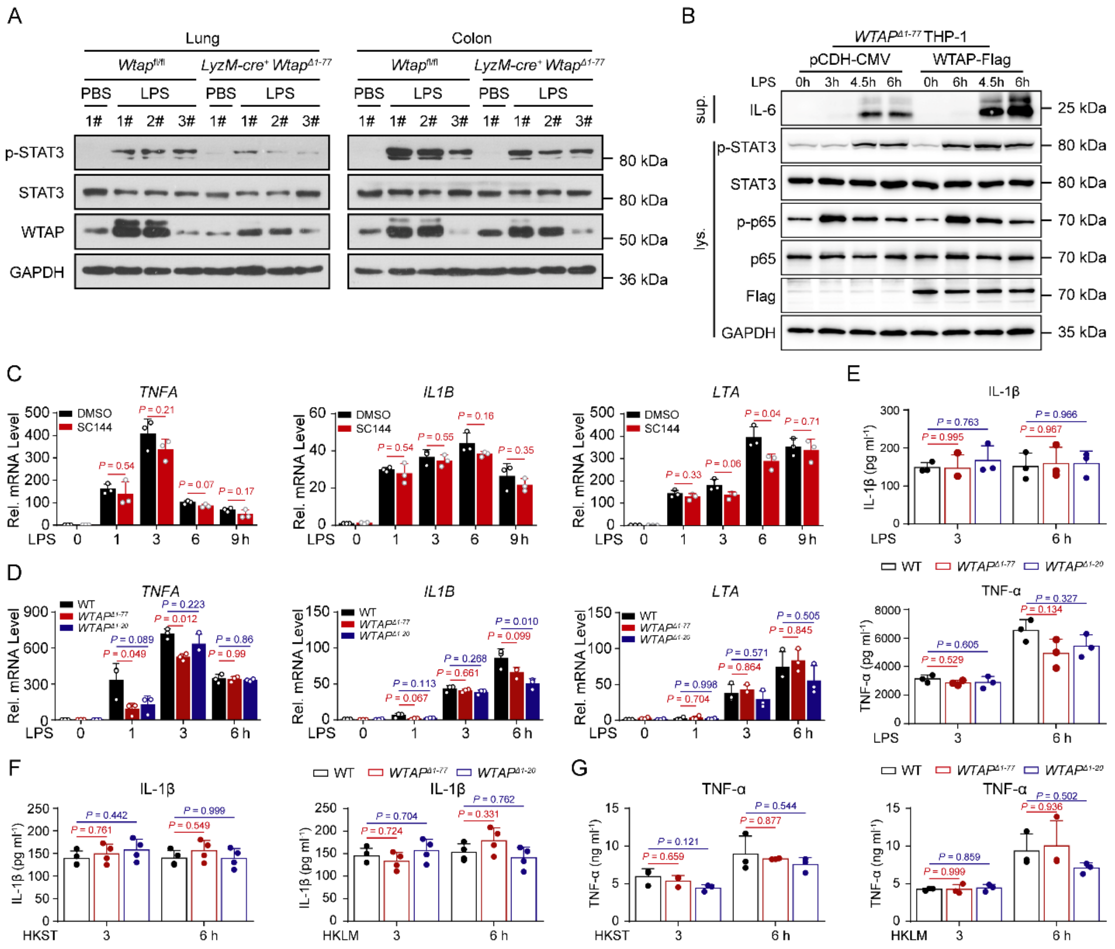

Supplemental Figure 7. WTAP deficiency does not affect the expression of IL-1β and TNF-α. (**A**) Immunoblots showing the total and phosphorylated STAT3 level in the lung or colon tissues from *Wtap*$^{fl/fl}$ and *LyzM-cre*$^{+}$ *Wtap*$^{\Delta 1\text{-}77}$ mice that had been intraperitoneally injected with LPS for 6 hr. (**B**) Immunoblots showing total and phosphorylated STAT3 and p65 levels in *WTAP*$^{\Delta 1\text{-}77}$ THP-1 cells transfected with Flag-tagged WTAP, followed by stimulation with LPS at different time points. (**C**) qRT–PCR showing the expression of *TNFA*, *IL1B* and *LTA* in THP-1 cells treated with SC144, followed by stimulation with LPS at the indicated time points. (**D**) qRT–PCR showing the expression of *TNFA*, *IL1B* and *LTA* in WT and *WTAP*$^{\Delta 1\text{-}77}$ THP-1 cells that were stimulated with LPS at 3 and 6 hr. (**E-G**) ELISAs were performed to measure IL-1β and TNF-α secretion in supernatants of WT and *WTAP*$^{\Delta 1\text{-}77}$ THP-1 cells that were stimulated with LPS (E), HKST (F) or HKLM (G) at 3 and 6 hr.

Data are presented as the mean ± s.d. in (C to G), with individual measurements overlaid as dots, statistical analysis was performed using two-tailed Student's *t*-test in (C), or one-way ANOVA

multiple comparisons in (D to G). Data are representative of three independent biological experiments in (A) and (B).

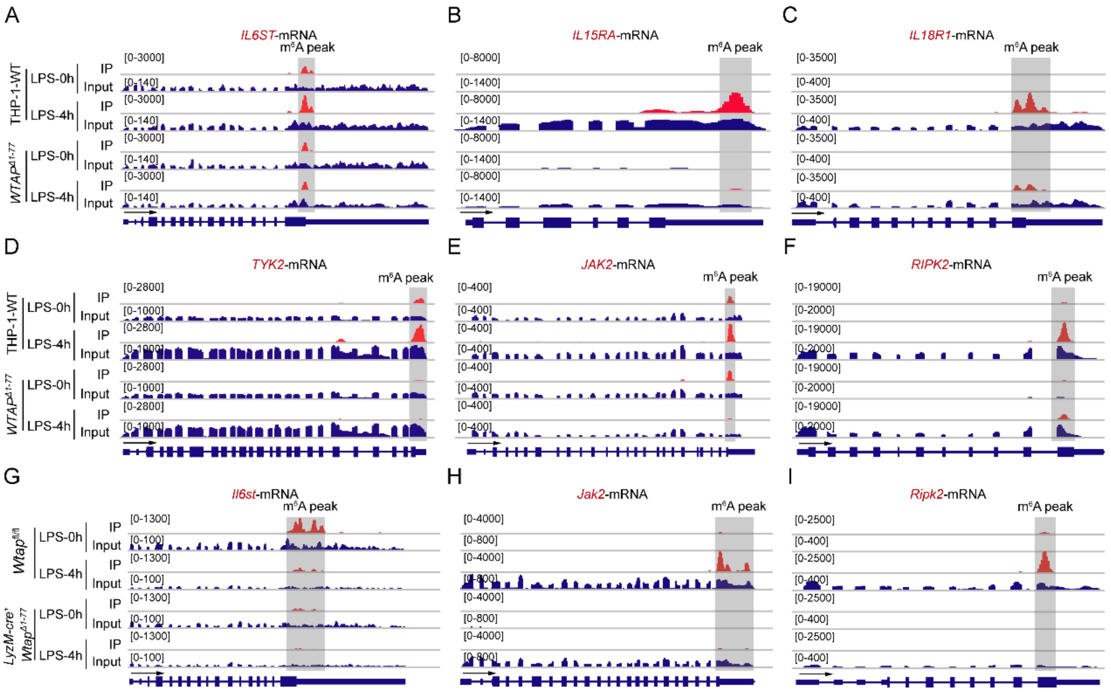

Supplemental Figure 8. WTAP deficiency decreases the m^{6}A modification of many inflammatory genes. (**A** to **F**) Integrative Genomics Viewer (IGV) browser tracks showing that m^{6}A peaks were enriched in the 3' UTR or CDS regions of *IL6ST* (A), *IL15RA* (B), *IL18R1* (C), *TYK2* (D), *JAK2* (E) and *RIPK2* (F) transcripts. (**G** to **I**) IGV browser tracks showing that m^{6}A peaks were enriched in the 3' UTR or CDS regions of *Il6st* (G), *Jak2* (H), and *Ripk2* (I) transcripts.

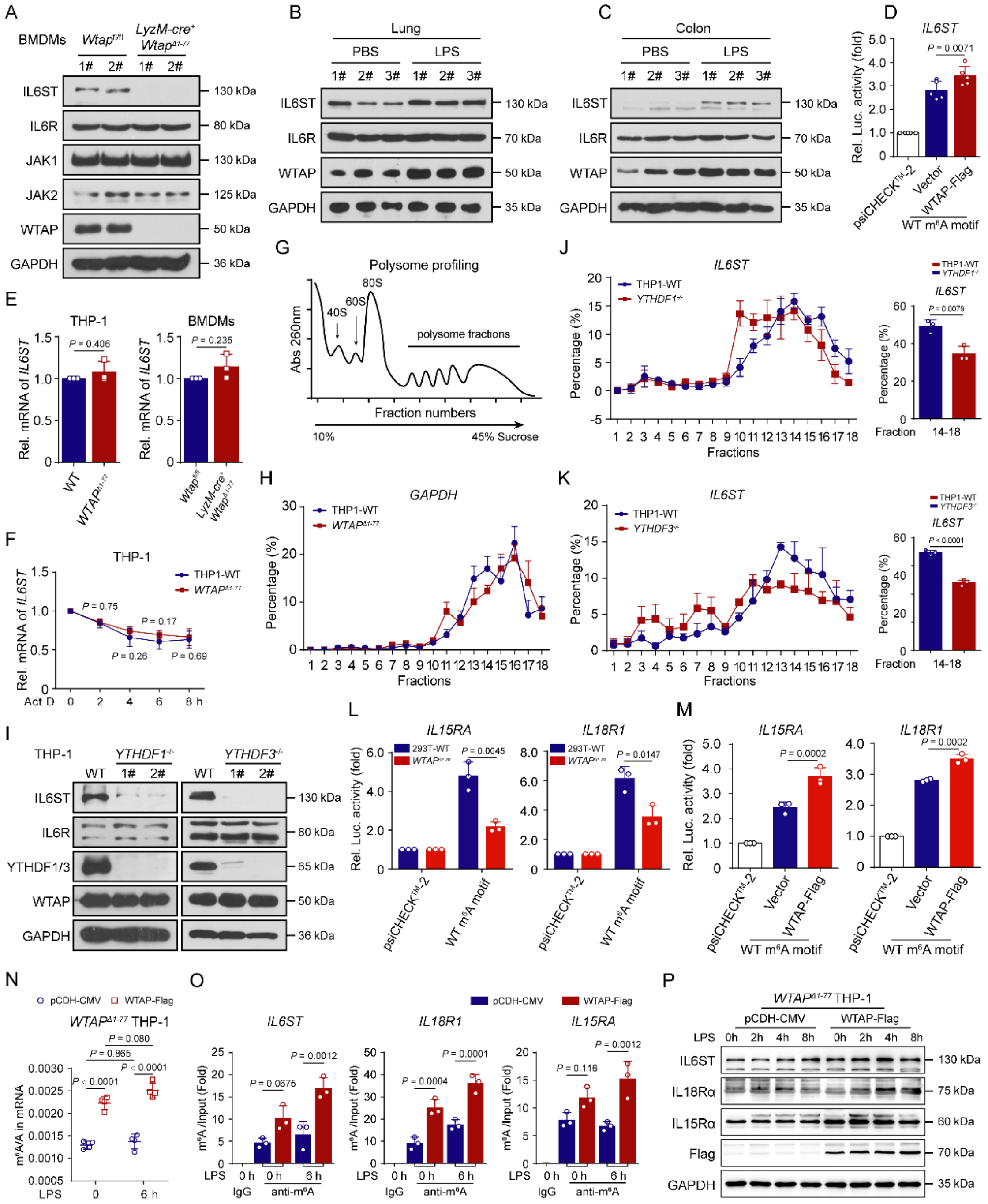

Supplemental Figure 9. WTAP promotes the protein expression of proinflammatory genes through m^{6}A modification. (**A**) Immunoblots showing the expression of critical adaptors in the IL-6/STAT3 signaling pathway in BMDMs from *Wtap*$^{fl/fl}$ or *LyzM*-Cre$^+$ *Wtap*$^{\Delta 1\text{-}77}$ mice. (**B** and **C**) Immunoblots showing the expression of WTAP and IL6ST in in the lung (B) or colon (C) tissues from wild-type mice that had been intraperitoneally injected with LPS or PBS for 12 hr. (**D**) Relative luciferase activities of reporter vectors bearing *IL6ST*-3' UTR with wild-type m^{6}A sites after co-transfection with empty vector (EV) or Flag-tagged WTAP into *WTAP* KO 293T cells. (**E**) qRT–PCR showing the mRNA abundance of *IL6ST* in WT and *WTAP*$^{\Delta 1\text{-}77}$ THP-1 cells or BMDMs. (**F**)

qRT–PCR showing the mRNA abundance of *IL6ST* in WT and *WTAP*$^{Δ1-77}$ THP-1 cells treated with Act D at the indicated time points. (**G**) Schematic representation of the polysome profiling. (**H**) qRT–PCR showing the proportion of *GAPDH* mRNA in polysome fractions from WT and *WTAP*$^{-/-}$ THP-1 cells treated with LPS for 6 hr. (**I**) Immunoblots showing the expression of IL6ST in WT and *YTHDF1*$^{-/-}$ or *YTHDF3*$^{-/-}$ THP-1 cells. (**J** and **K**) qRT–PCR showing the proportion of *IL6ST* mRNA in polysome fractions from WT and *YTHDF1*$^{-/-}$ (J) or *YTHDF3*$^{-/-}$ (K) THP-1 cells. Relative enrichment of IL6ST mRNA in polysome part (fraction 14–18) was showed in left. (**L**) Relative luciferase activities in WT and *WTAP* KO 293T cells after transfection with reporter vectors bearing the *IL15RA*- or *IL18R1*-3' UTR with WT m^{6}A sites. (**M**) Relative luciferase activities of reporter vectors bearing *IL15RA*- or *IL18R1*-3' UTR with wild-type m^{6}A sites after co-transfection with empty vector (EV) or Flag-tagged WTAP into *WTAP* KO 293T cells. (**N**) LC–MS/MS quantification of m^{6}A abundance in mRNA extracted from *WTAP*$^{Δ1-77}$ THP-1 cells transfected with empty vector or Flag-tagged WTAP, followed by stimulation with LPS. (**O**) The abundance of *IL6ST*, *IL15RA* or *IL18R1* transcripts among mRNAs immunoprecipitated with an anti-m^{6}A antibody in *WTAP*$^{Δ1-77}$ THP-1 cells transfected with empty vector or Flag-tagged WTAP, followed by stimulation with LPS. (**P**) Immunoblots analyses showing the protein abundance of IL6ST, IL18Rα and IL15Rα in *WTAP*$^{Δ1-77}$ THP-1 cells transfected with empty vector or Flag-tagged WTAP, followed by stimulation with LPS at different time points.

Data are representative of three independent biological experiments in (A to C), (I) and (P). Data are presented as the mean ± s.d. in (D to F), (H) and (J to O), with individual measurements overlaid as dots. Statistical analysis was performed using one-way ANOVA multiple comparisons in (D) and (M to O), or two-tailed Student's *t*-test in (E), (F), and (J to L).

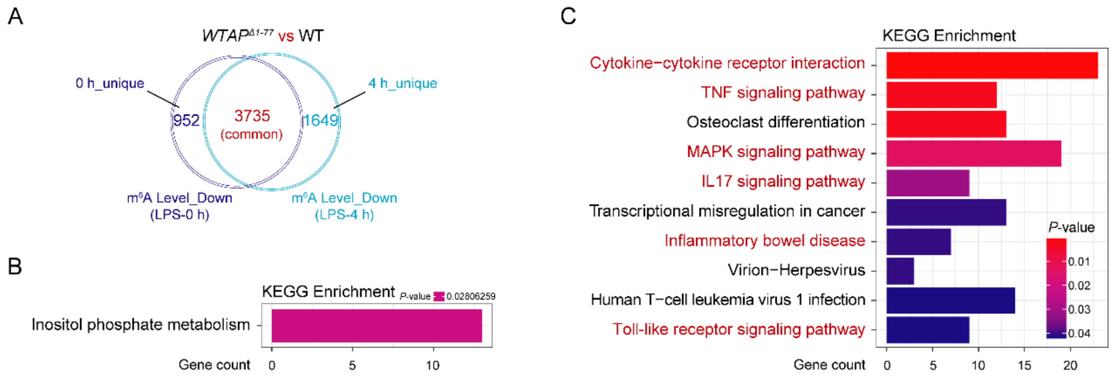

Supplemental Figure 10. WTAP is deeply involved in the regulation of inflammatory response. (**A**) Venn diagrams showing transcripts with decreased m^{6}A abundance in *WTAP*$^{\Delta 1\text{-}77}$ THP-1 cells compared with WT cells stimulated with or without LPS. (**B**) KEGG enrichment analysis of the 952 transcripts with decreased m^{6}A abundance only at resting state. (**C**) KEGG enrichment analysis of the 1649 transcripts with decreased m^{6}A abundance only after treatment with LPS for 4 hr.

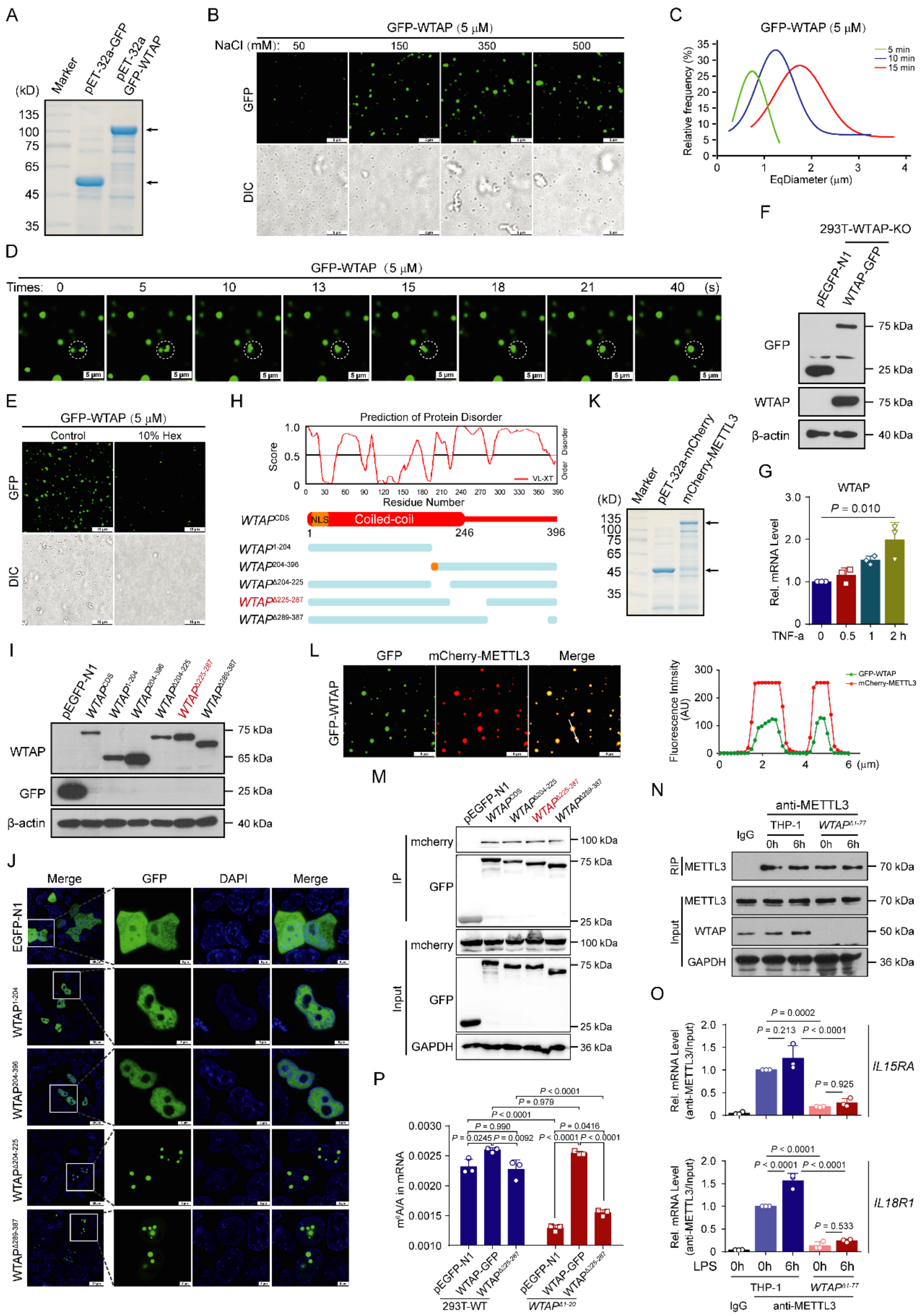

Supplemental Figure 11. Phase separation of WTAP leads to METTL3 recruitment to efficiently modify inflammatory transcripts. (**A**) Bacterial purified GFP-WTAP proteins were analyzed by SDS-PAGE and detected by coomassie blue staining. (**B**) Images of GFP-WTAP droplets formation at room temperature with indicated NaCl concentrations (5 μM GFP-WTAP).

(**C**) EqDiameter (the diameter of a circle with the same area as the measured object) frequency distribution of GFP-WTAP liquid droplets formed at the indicated timepoints (350 mM NaCl, 5 μM GFP-WTAP). (**D**) Time-lapse micrographs of the fusion at room temperature of GFP-WTAP (5 μM) liquid droplets formed at the indicated times. (**E**) GFP-WTAP were treated with 5% Hex and then subjected to droplet formation assay *in vitro* (350 mM NaCl, 5 μM GFP-WTAP). (**F**) Immunoblots showing of the expression of GFP-tagged WTAP. (**G**) qRT–PCR showing the mRNA abundance of *WTAP* in HeLa cells stimulated with TNF-α at the indicated time points. (**H**) Prediction of the unfolded intrinsically disordered regions (IDRs) of WTAP using the PONDR tool, and a series of truncated WTAP labeled with GFP are as shown. (**I** and **J**) Immunoblots (I) and representative confocal images (J) of 293T expressing truncated WTAP labeled with GFP. **(K)** Bacterial purified mCherry-METTL3 proteins were analyzed by SDS-PAGE and detected by coomassie blue staining. (**L**) Fluorescence of mCherry-METTL3 (10 μM) and GFP-WTAP (10 μM) mixed at room temperature with 350 mM NaCl. Quantitative line profile of colocalization along a white arrow of the image on the left. (**M**) Coimmunoprecipitation and immunoblot analyses of extracts of 293T cells transfected with various combinations of plasmids encoding GFP-tagged WTAP and mCherry-tagged METTL3. (**N** and **O**) METTL3 was immunoprecipitated (N) and RIP-qPCR was performed to assess the association of *IL15RA* or *IL18R1* transcripts with METTL3 (O). (**P**) LC–MS/MS quantification of m^6A abundance in mRNA extracted from WT and $WTAP^{\Delta 1\text{-}20}$ 293T cells transfected with indicated plasmids.

Data are representative of three independent biological experiments in (A to F), (I to N) and (P). Data are presented as the mean ± s.d. in (G), (O) and (P), with individual measurements overlaid as dots. Statistical analysis was performed using two-tailed Student's *t*-test in (G), or one-way ANOVA multiple comparisons in (O) and (P). Indicated scale bars are shown in (B), (D), (E), (J) and (L).

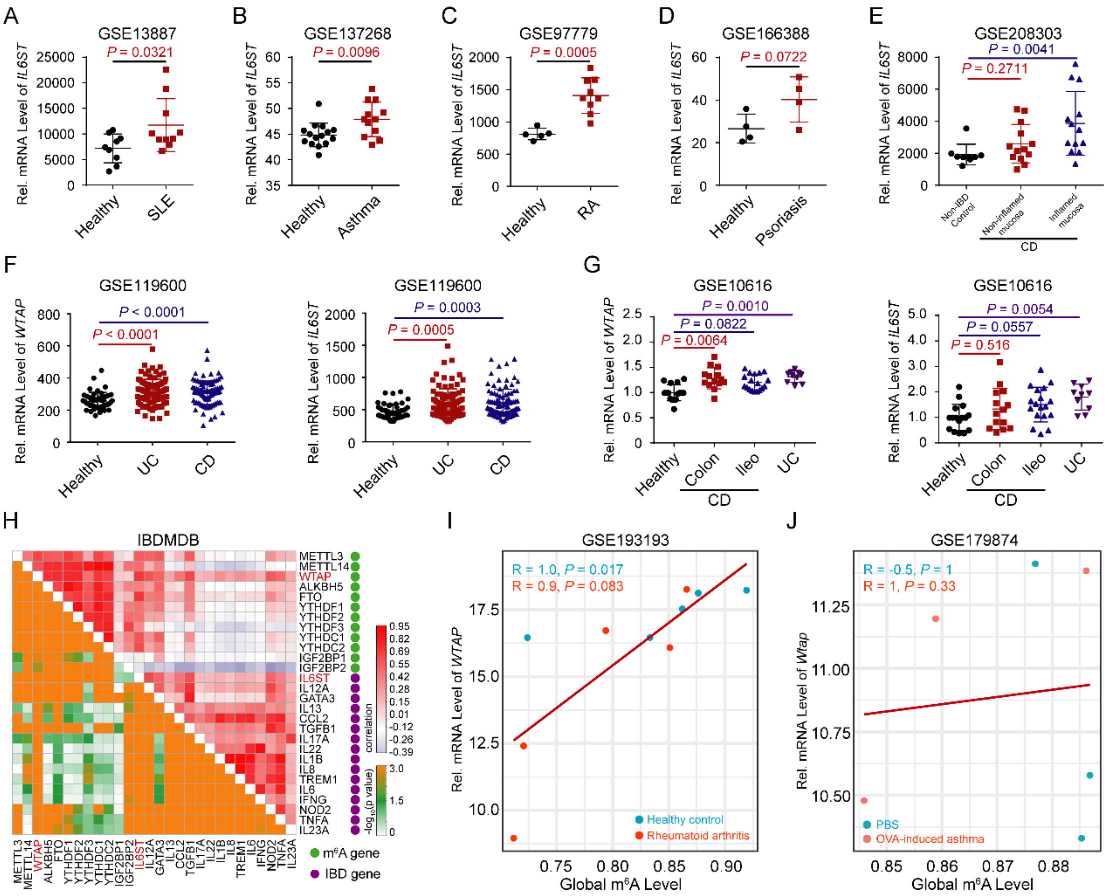

Supplemental Figure 12. The expression of WTAP and IL6ST is increased in patients with inflammatory diseases. (**A-E**) The mRNA abundance of *IL6ST* in patients with SLE, asthma, RA, psoriasis and IBD compared with those in healthy controls. (**F** and **G**) The mRNA abundance of *WTAP* and *IL6ST* in patients with IBD compared with healthy controls. (**H**) Spearman correlations between m^{6}A regulators and IBD core cytokines in IBDMDB patients. (**I** and **J**) Spearman correlations between the expression of WTAP and the global m^{6}A abundance in RA patients (I), or in ovalbumin (OVA)-induced acute allergic asthma mice (J).

Data are presented as the mean ± s.d. in (A to C), with individual measurements overlaid as dots. Statistical analysis was performed using Mann–Whitney U test in (A to D), or Kruskal–Wallis test in (E to G).

Supplemental tables

Supplementary Table 1. The brief descriptions of the inclusive data series Source

	**Subjects analysed (sample size)**					**Type of sample**	**Type of data**
GSE19315	Untreated (3)	LPS-treated (3)				THP-1 cells	mRNA
GSE198326	DMSO-treated (3)	LPS-treated (3)				Macrophages of healthy male	mRNA
GSE2411	Untreated (6)	LPS-treated (6)				Mouse lung homogenate	mRNA
GSE2638	Untreated (3)	TNF-treated (3)				HMEC	mRNA
GSE69063	Healthy controls (33)	Sepsis patients (57)				Peripheral blood samples	mRNA
GSE13887	Healthy controls (9)	SLE patients (10)				T cells	mRNA
GSE137268	Healthy controls (15)	Severe asthma patients (12)				Sputum	mRNA
GSE97779	Healthy controls (5)	Rheumatoid arthritis patients (9)				Synovial macrophages	mRNA
GSE166388	Healthy controls (4)	Psoriasis patients (4)				Skin epidermis	mRNA
GSE208303	Healthy mucosa of non-IBD (9)	Non-inflamed mucosa in patients with Crohn's disease (13)	Inflamed mucosa in patients with Crohn's disease (13)			Intestinal mucosa	mRNA
GSE16879	Normal mucosa from	Ileal mucosal from Crohn's ileitis	Ileal mucosal from Crohn's ileitis	Ileal mucosal from Crohn's ileitis non-	Ileal mucosal from Crohn's ileitis non-	Ileal mucosa	mRNA

	control patients (6)	responder before first infliximab treatment (8)	responder after first infliximab treatment (8)	responder before first infliximab treatment (10)	responder after first infliximab treatment (10)			
GSE137218	Nonlesional psoriatic skin of psoriatic patients（14）	Lesional psoriatic skin of psoriatic patients before secukinumab treatment（14）	Lesional psoriatic skin at day 4 during secukinumab treatment（14）	Lesional psoriatic skin at day 14 during secukinumab treatment（14）	Lesional psoriatic skin at day 42 during secukinumab treatment（14）	Lesional psoriatic skin at day 84 during secukinumab treatment（14）	Skin	mRNA
GSE125527	Healthy controls (8)			Ulcerative colitis (7)			Rectal CD45^{+} immune cells	mRNA
GSE193193	Healthy controls (5)			PBMC of rheumatoid arthritis patient (5)			Peripheral blood mononuclear cells	mRNA
GSE179874	PBS-induced control mice (3)			OVA-induced asthma mice (3)			Lung tissues in mouse model	mRNA
GSE119600	Healthy controls (47)		Ulcerative colitis (93)		Crohn’s disease (95)		Blood	mRNA
GSE10616	Healthy controls (16)		Colon-only Crohn's Disease (14)		Ileo-only Crohn's Disease (18)		Ulcerative Colitis (10)	Colon/Ileal

GSE227851	Uninfected (6)		H37Rv-infected (8)				Monocyte differentiated to macrophages with PMA	mRNA
GSE189847	Uninfected M0 (4)	SL1344-infected M0 (3)	Uninfected M1 (4)	SL1344-infected M1 (4)	Uninfected M2 (4)	SL1344-infected M2 (3)	Monocyte-derived macrophages	mRNA

Supplementary Table 2. Predicted transcription factors that binding to *WTAP* promoter

Name	Score	Relative score	Start	End	Strand	Predicted sequence
C/EBPβ	15.449436	0.981882518	1848	1858	+	gattgcaccac
C/EBPβ	14.261012	0.969425289	1713	1723	-	gatttcaccat
IRF3	9.186087	0.817418043	1384	1404	+	aaagaaaggaaagggaaggaa
p65	8.949939	0.834631296	1717	1726	-	tgggatttca
STAT3	8.424692	0.89961612	506	516	+	cttcttaaaat
p65	8.096203	0.812290467	86	95	-	ggggttttct
p65	8.085627	0.812013705	68	77	-	tggactttca
IRF3	7.820719	0.806317005	1972	1992	-	gttgaaagcaaattgaaacaa
STAT3	7.029458	0.882713481	1631	1641	-	attacaggaat
STAT3	6.6519017	0.878139555	1631	1641	+	attcctgtaat
C/EBPβ	6.5461287	0.888556797	210	220	+	tgttgcccaag
HIF1α	5.4975653	0.82156562	1135	1144	+	gcacatgcct
STAT3	5.4137197	0.863139531	1263	1273	-	tttttgagaac
STAT3	5.282355	0.861548105	679	689	-	atgcctgtaat
STAT3	5.282355	0.861548105	1768	1778	+	atgcctgtaat
HIF1α	5.2455807	0.815632422	552	561	-	agacgttcat
STAT3	5.1454854	0.859889993	496	506	-	gttcagaaaaa
HIF1α	5.028608	0.810523608	1133	1142	-	gcatgtgcca
STAT3	4.954211	0.857572791	578	588	+	ttttttaaaaa
C/EBPβ	15.449436	0.981882518	1848	1858	+	gattgcaccac
C/EBPβ	14.261012	0.969425289	1713	1723	-	gatttcaccat

The predicted results were analyzed by the JASPAR (http://jaspar.genereg.net/).

Supplementary Table 3. List of oligonucleotides used in this study

WTAP sgRNA sequence (5'-3')-1: CACCGCTTGGGAAGAGGTTCTTCGT
WTAP sgRNA sequence (5'-3')-2: CACCGCAAGAGATGAGTTAATTCTA
IL6ST sgRNA sequence (5'-3')-1: CACCGTGCAACGTCAACATCTTGCG
IL6ST sgRNA sequence (5'-3')-2: CACCGAGATGTTGACGTTGCAGACT
YTHDF1 sgRNA sequence (5'-3')-1: CACCGAAATGGTTCGTTACATCAGA
YTHDF1 sgRNA sequence (5'-3')-2: CACCGAGACCCAGAGCTCCGCGTAT
YTHDF3 sgRNA sequence (5'-3')-1: CACCGTTTGTCTGGCTACTTAAGTA
YTHDF3 sgRNA sequence (5'-3')-2: CACCGTGGACTATAATGCGTATGC
Human *GAPDH* Forward 5'-TGTTGCCATCAATGACCCCTT-3'
Human *GAPDH* Reverse 5'-CTCCACGACGTACTCAGCG-3
Mouse *Gapdh* Forward 5'-TGACCTCAACTACATGGTCTACA-3'
Mouse *Gapdh* Reverse 5'-CTTCCCATTCTCGGCCTTG-3'
Human *ACTB* Forward 5'-CATGTACGTTGCTATCCAGGC-3'
Human *ACTB* Reverse 5'-CTCCTTAATGTCACGCACGAT-3'
Mouse *Actb* Forward 5'-GTGACGTTGACATCCGTAAAGA-3'
Mouse *Actb* Reverse 5'-GCCGGACTCATCGTACTCC-3'
Human *WTAP* Forward 5'-ACTGGCCTAAGAGAGTCTGAAG-3'
Human *WTAP* Reverse 5'-GTTGCTAGTCGCATTACAAGGA-3'
Mouse *Wtap* Forward 5'-GAACCTCTTCCTAAAAAGGTCCG-3'
Mouse *Wtap* Reverse 5'-TTAACTCATCCCGTGCCATAAC-3'
Human *METTL3* Forward 5'-TTGTCTCCAACCTTCCGTAGT-3'
Human *METTL3* Reverse 5'-CCAGATCAGAGAGGTGGTGTAG-3'
Mouse *Mettl3* Forward 5'-CTGGGCACTTGGATTTAAGGAA-3'
Mouse *Mettl3* Reverse 5'-TGAGAGGTGGTGTAGCAACTT-3'
Human *METTL14* Forward 5'-AGTGCCGACAGCATTGGTG-3'
Human *METTL14* Reverse 5'-GGAGCAGAGGTATCATAGGAAGC-3'
Mouse *Mettl14* Forward 5'-CTGAGAGTGCGGATAGCATTG-3'
Mouse *Mettl14* Reverse 5'-GAGCAGATGTATCATAGGAAGCC-3'

Human *IL1B* Forward 5’-ATGATGGCTTATTACAGTGGCAA-3’
Human *IL1B* Reverse 5’-GTCGGAGATTCGTAGCTGGA-3’
Mouse Il1b Forward5’-CTGTGACTCATGGGATGATGATG-3’
Mouse *Il1b* Reverse 5’-CGGAGCCTGTAGTGCAGTTG-3’
Human *TNFA* Forward 5’-CCTCTCTCTAATCAGCCCTCTG-3’
Human *TNFA* Reverse 5’-GAGGACCTGGGAGTAGATGAG-3’
Mouse *Tnfa* Forward 5’-CCTGTAGCCCACGTCGTAG-3’
Mouse *Tnfa* Reverse 5’-GGGAGTAGACAAGGTACAACCC-3’
Human *LTA* Forward 5’-ATGACACCACCTGAACGTCTC-3’
Human *LTA* Reverse 5’-CTCTCCAGAGCAGTGAGTTCT-3’
Human *IL6* Forward 5’-ACTCACCTCTTCAGAACGAATTG-3’
Human *IL6* Reverse 5’-CCATCTTTGGAAGGTTCAGGTTG-3’
Mouse *Il6* Forward 5’-CTGCAAGAGACTTCCATCCAG-3’
Mouse *Il6* Reverse 5’-AGTGGTATAGACAGGTCTGTTGG-3’
Human *CCL2* Forward 5’-CAGCCAGATGCAATCAATGCC-3’
Human *CCL2* Reverse 5’-TGGAATCCTGAACCCACTTCT-3’
Mouse *Ccl2* Forward 5’-TTAAAAACCTGGATCGGAACCAA-3’
Mouse *Ccl2* Reverse 5’-GCATTAGCTTCAGATTTACGGGT-3’
Human *CCL8* Forward 5’-TGGAGAGCTACACAAGAATCACC-3’
Human *CCL8* Reverse 5’-TGGTCCAGATGCTTCATGGAA-3’
Mouse *Ccl8* Forward 5’-TCTACGCAGTGCTTCTTTGCC-3’
Mouse *Ccl8* Reverse 5’-AAGGGGGATCTTCAGCTTTAGTA-3’
Human *CXCL8* Forward 5’-ACTGAGAGTGATTGAGAGTGGAC-3’
Human *CXCL8* Reverse 5’-AACCCTCTGCACCCAGTTTTC-3’
Human *IL6ST* Forward 5’-CGGACAGCTTGAACAGAATGT-3’
Human *IL6ST* Reverse 5’-ACCATCCCACTCACACCTCA-3’
Mouse *Il6st* Forward 5’-CCGTGTGGTTACATCTACCCT-3’
Mouse *Il6st* Reverse 5’-CGTGGTTCTGTTGATGACAGTG-3’
IL6ST-m^{6}A site 1 Forward 5’-CTGTGGATCTGGGCAAATGAAAA-3’

IL6ST-m^{6}A site 1 Reverse 5’-ACTGCTGAAGTTGTAGCAGGA-3’
IL6ST-m^{6}A site 2 Forward 5’-AAGCCTTTTCCAGAAGATCTGA-3’
IL6ST-m^{6}A site 2 Reverse 5’-ACTGGACAGTGCTCGAAGTG-3’
IL18R1-m^{6}A site Forward 5’-GTGGACTCCATGAAGCATTGG-3’
IL18R1-m^{6}A site Reverse 5’-GGGGCAAGAATGTGAAGTCAG-3’
IL15RA-m^{6}A site Forward 5’-CTGCTCTGCACACATGGACA-3’
IL15RA -m^{6}A site Reverse 5’-TCAATGGAGAGGATTCGCTGG-3’

Supplementary Table 4. List of reagents and resources used in this study

REAGENT or RESOURCE	SOURCE	IDENTIFIER
**Antibodies**		
p65	CST	Cat# 8242
Phospho-p65	CST	Cat# 3033
p38	CST	Cat# 8690
Phospho-p38	CST	Cat# 4511
STAT3	CST	Cat# 9139
Phospho-STAT3	CST	Cat# 9145
GFP	CST	Cat# 2555
METTL14	CST	Cat# 51104
m^{6}A antibody	CST	Cat# 56593
WTAP	Abcam	Cat# ab195380
WTAP (mouse monoclonal/ rabbit polyclonal)	Proteintech	Cat# 60188-1-Ig/ 10200-1-AP
METTL3	Proteintech	Cat# 15073-1-AP
VCL	Proteintech	Cat# 66305-1-Ig
JAK1	Proteintech	Cat# 66466-1-AP
JAK2	Proteintech	Cat# 17670-1-AP
IL6R	Proteintech	Cat# 23457-1-AP
YTHDF1	Proteintech	Cat# 17479-1-AP
GAPDH	Proteintech	Cat# 10494-1-AP
β-actin	Proteintech	Cat# 66009-1-Ig
Flag	Proteintech	Cat# 66008-4-Ig
HA	Proteintech	Cat# 66006-2-Ig
Goat anti-Mouse IgG	Proteintech	Cat# SA00001-1
Goat anti-Rabbit IgG	Proteintech	Cat# SA00001-2
TYK2	Abclonal	Cat# A2128
IL6ST	Abclonal	Cat# A14656
IL18R1	Abclonal	Cat# A21208
IL15RA	Abclonal	Cat# A2983
YTHDF2	Abclonal	Cat# A15616
YTHDF3	Abclonal	Cat# A8395
Goat anti-Rabbit IgG, Alexa Fluor™ 488	Thermo Fisher	Cat# A-11008
Goat anti-Mouse IgG, Alexa Fluor™ 488	Thermo Fisher	Cat# A-10680
Goat anti-Rabbit IgG, Alexa Fluor™ 568	Thermo Fisher	Cat# A-11011
**Reagents**		
LPS (From E. coli O111:B4)	InvivoGen	Cat# tlrl-eblps
Pam3CSK4	InvivoGen	Cat# tlrl-pms
CL097	InvivoGen	Cat# tlrl-c97
HKST	InvivoGen	Cat# tlrl-hkst2
HKLM	InvivoGen	Cat# tlrl-hklm

PG490	Selleck	Cat# S3604
SN50	Selleck	Cat# S6671
SC144	Selleck	Cat# S7124
STM2457	Selleck	Cat# S9870
Lipofectamine 2000	Thermo Fisher	Cat# 11668019
Lipofectamine 3000	Thermo Fisher	Cat# L3000015
Lipofectamine RNAiMAX	Thermo Fisher	Cat# 13778030
High Capacity Streptavidin Agarose Resin	Thermo Fisher	Cat# 20359
Recombinant Murine M-CSF	PeproTech	Cat# 315-02
PMA	Sigma-Aldrich	Cat# P1585
Actinomycin D	Sigma-Aldrich	Cat# A1410
Brefeldin A	Sigma-Aldrich	Cat# B5936
Methylene blue	Sigma-Aldrich	Cat# M9140
Protein G Sepharose	Abcam	Cat# ab193259
TRIzol reagent	Invitrogen	Cat# 15596026
**Experimental models: Organisms/strains**		
LyzM-Cre^{+} *Wtap*$^{\Delta 1\text{-}77}$ mice (C57BL/6 background)	Generated for this study by Gempharmatech Co., Ltd..	N/A
Pseudomonas aeruginosa	ATCC	ATCC27853
Listeria monocytogenes	ATCC	ATCC19116
**Experimental models: Cell lines**		
HEK293T cells	ATCC	N/A
HeLa cells	ATCC	N/A
THP-1 cells	ATCC	N/A
WTAP$^{\Delta 1\text{-}20}$ THP-1 cells		
WTAP$^{\Delta 1\text{-}77}$ THP-1 cells	This paper	N/A
WTAP$^{\Delta 1\text{-}20}$ HEK293T cells	This paper	N/A
IL6ST$^{-/-}$ THP-1 cells	This paper	N/A
YTHDF1$^{-/-}$ THP-1 cells	This paper	N/A
YTHDF3$^{-/-}$ THP-1 cells	This paper	N/A
**Oligonucleotides**		
Primers used for qRT-PCR experiments, see Table S2	This paper	N/A
siRNA and sgRNA sequences, see Table S2	This paper	N/A
**Critical commercial assays**		
PrimeScript^{TM} RT reagent kit with gDNA Eraser	TaKaRa	Cat# RR047A
Evo M-MLV RT Mix Kit with gDNA Clean for qPCR	AG	Cat# AG11728
2× RealStar Power SYBR qPCR Mix	GenStar	Cat# A311-01
2×Polarsignal® qPCR mix	MIKX	Cat# MKG802
Human IL-1 beta Quantikine ELISA Kit	R&D Systems	Cat# DLB50
Human TNF-alpha Quantikine QuicKit ELISA	R&D Systems	Cat# QK210
Human IL-6 ELISA Kit	Proteintech	Cat# KE00139

Mouse IL-6 ELISA Kit	Proteintech	Cat# KE10007
Dual-Luciferase® Reporter Assay System	Promega	Cat# E1960
RIP-Assay Kit	MBL	Cat# RN1001
RNeasy Mini Kit	QIAGEN	Cat# 74104
Magna MeRIP m^6A Kit	Merck Millipore	Cat# 17-10499
EpiQuik m^6A RNA Methylation Quantification Kit	EpiGentek	Cat# P-9005-48
**Recombinant DNA**		
Plasmid: pGL3 basic	Lab stock	N/A
Plasmid: psiCHECK™-2	Lab stock	N/A
Plasmid: pEGFP-N1	Lab stock	N/A
Plasmid: pET-32a-GFP/mCherry	Lab stock	N/A
Plasmid: lentiCRISPR v2	Lab stock	N/A
Plasmid: psPAX2	Lab stock	N/A
Plasmid: pVSV-G	Lab stock	N/A
Plasmid: pcDNA3.1-FLAG	Lab stock	N/A
Plasmid: pcDNA3.1-mCherry	Lab stock	N/A
Plasmid: pcDNA3.1-FLAG-C/EBPβ	This paper	N/A
Plasmid: pcDNA3.1-FLAG-IRF3	This paper	N/A
Plasmid: pcDNA3.1-FLAG-p65	This paper	N/A
Plasmid: pcDNA3.1-FLAG-WTAP	This paper	N/A
Plasmid: pcDNA3.1-mCherry-METTL3	This paper	N/A
Plasmid: pGL3 basic-*WTAP* promoter-2250 bp	This paper	N/A
Plasmid: pGL3 basic-*WTAP* promoter-900 bp	This paper	N/A
Plasmid: pGL3 basic-*WTAP* promoter-p65/IRF3	This paper	N/A
Plasmid: pGL3 basic-*WTAP* promoter-C/EBPβ	This paper	N/A
Plasmid: pGL3 basic-*WTAP* promoter-p65-Del	This paper	N/A
Plasmid: pGL3 basic-*WTAP* promoter-p65-Mut	This paper	N/A
Plasmid: pGL3 basic-*Wtap* promoter-2190 bp	This paper	N/A
Plasmid: psiCHECKTM-2-IL6ST WT m^6A motif	This paper	N/A
Plasmid: psiCHECKTM-2-IL6ST Mut m^6A motif	This paper	N/A
Plasmid: psiCHECKTM-2-IL18R1 WT m^6A motif	This paper	N/A
Plasmid: psiCHECKTM-2-IL18R1 Mut m^6A motif	This paper	N/A
Plasmid: psiCHECKTM-2-IL15RA WT m^6A motif	This paper	N/A
Plasmid: psiCHECKTM-2-IL15RA Mut m^6A motif	This paper	N/A
Plasmid: pET-32a-GFP-WTAP	This paper	N/A
Plasmid: pET-32a-mCherry-METTL3	This paper	N/A
Plasmid: pEGFP-N1-$WTAP^{CDS}$	This paper	N/A
Plasmid: pEGFP-N1-$WTAP^{1-204}$	This paper	N/A
Plasmid: pEGFP-N1-$WTAP^{204-396}$	This paper	N/A
Plasmid: pEGFP-N1-$WTAP^{\Delta 204-225}$	This paper	N/A
Plasmid: pEGFP-N1-$WTAP^{\Delta 225-287}$	This paper	N/A
Plasmid: pEGFP-N1-$WTAP^{\Delta 289-387}$	This paper	N/A
Plasmid: pEGFP-N1-$WTAP^{\Delta 1-20}$	This paper	N/A

Plasmid: pEGFP-N1-WTAP$^{\Delta 1\text{-}77}$	This paper	N/A
Plasmid: pCDH-CMV	This paper	N/A
Plasmid: pCDH-CMV-WTAP-Flag	This paper	N/A
