# Supplementary material for: Elevated WTAP promotes hyperinflammation by increasing m6A modification in inflammatory disease models
Source: J Clin Invest. 2024 May 16;134(14):e177932. doi: 10.1172/JCI177932 (PMC11245160; doi:10.1172/JCI177932)

Uncropped blot for Figure 1

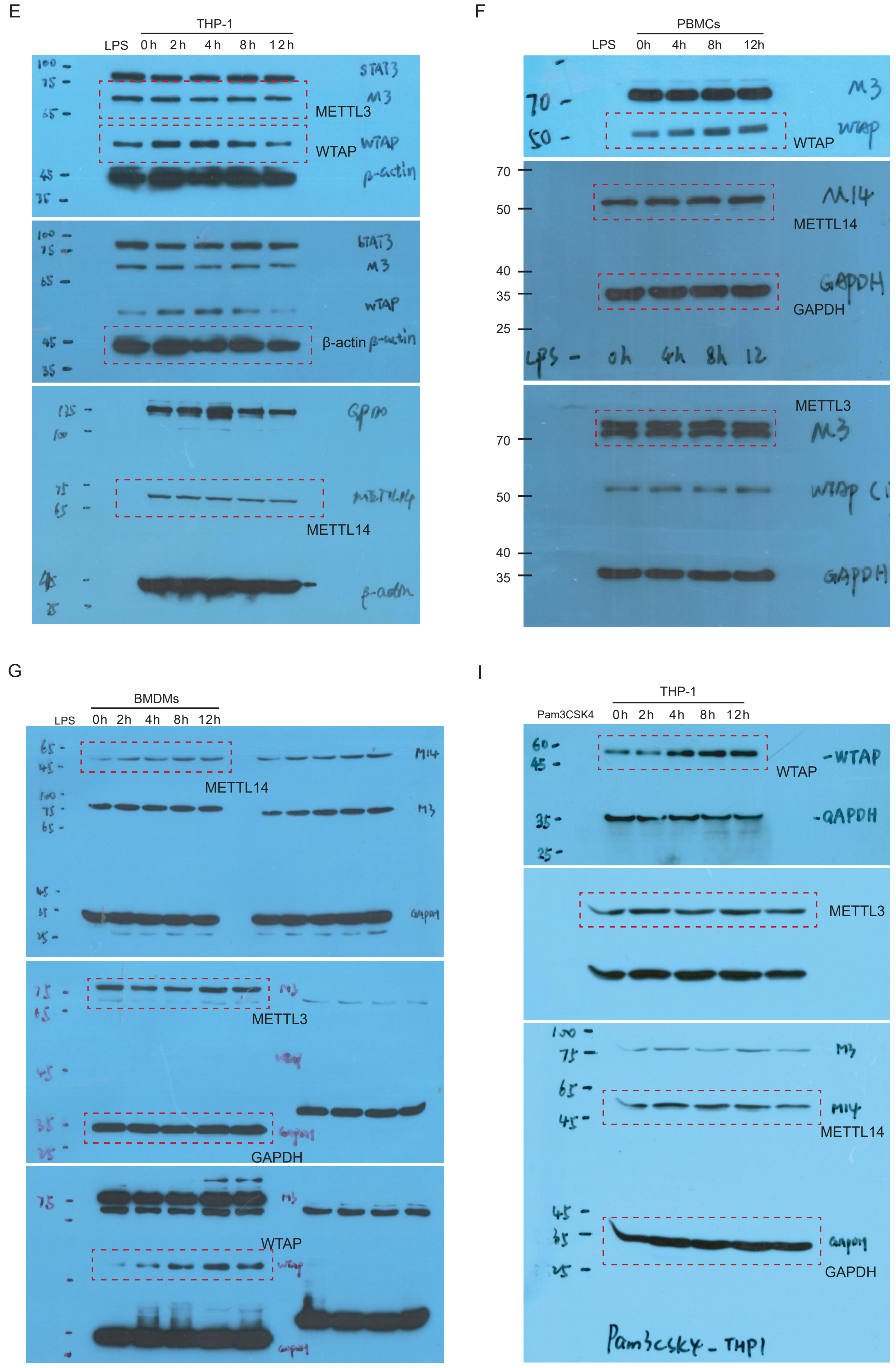

Uncropped blot for Figure 1

J

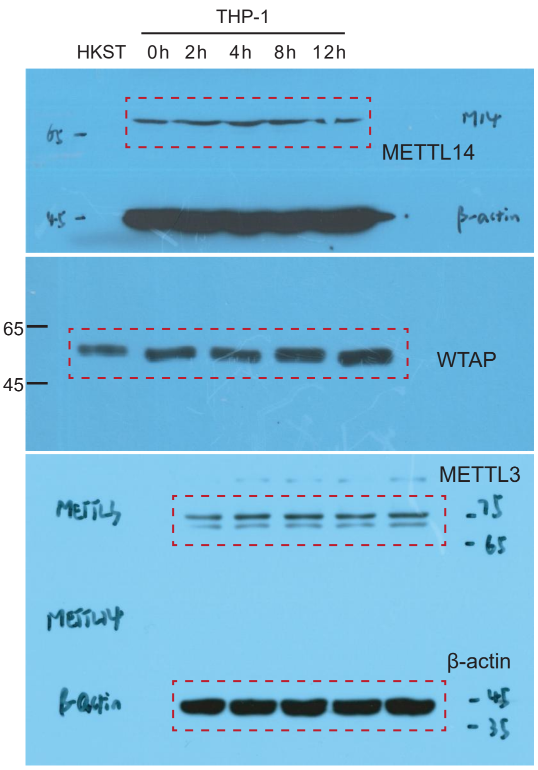

K

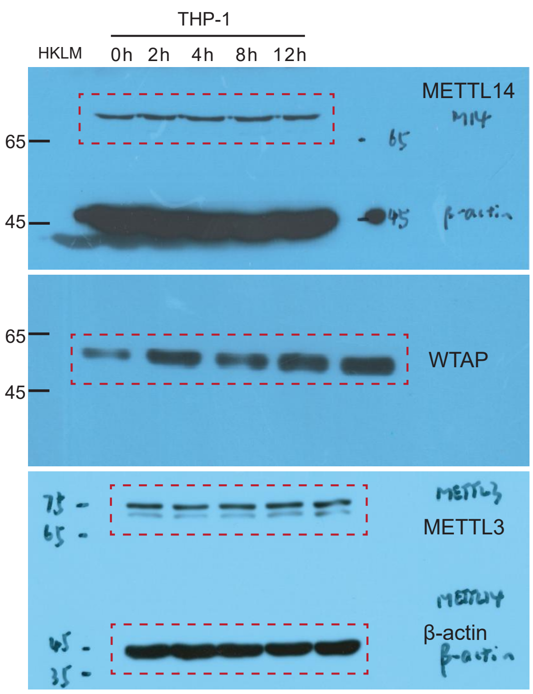

M

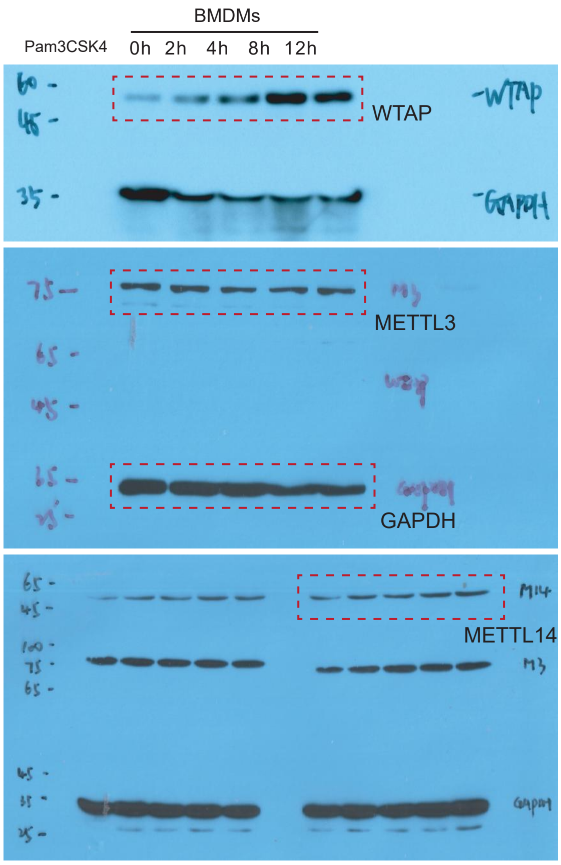

Uncropped blot for Figure 2

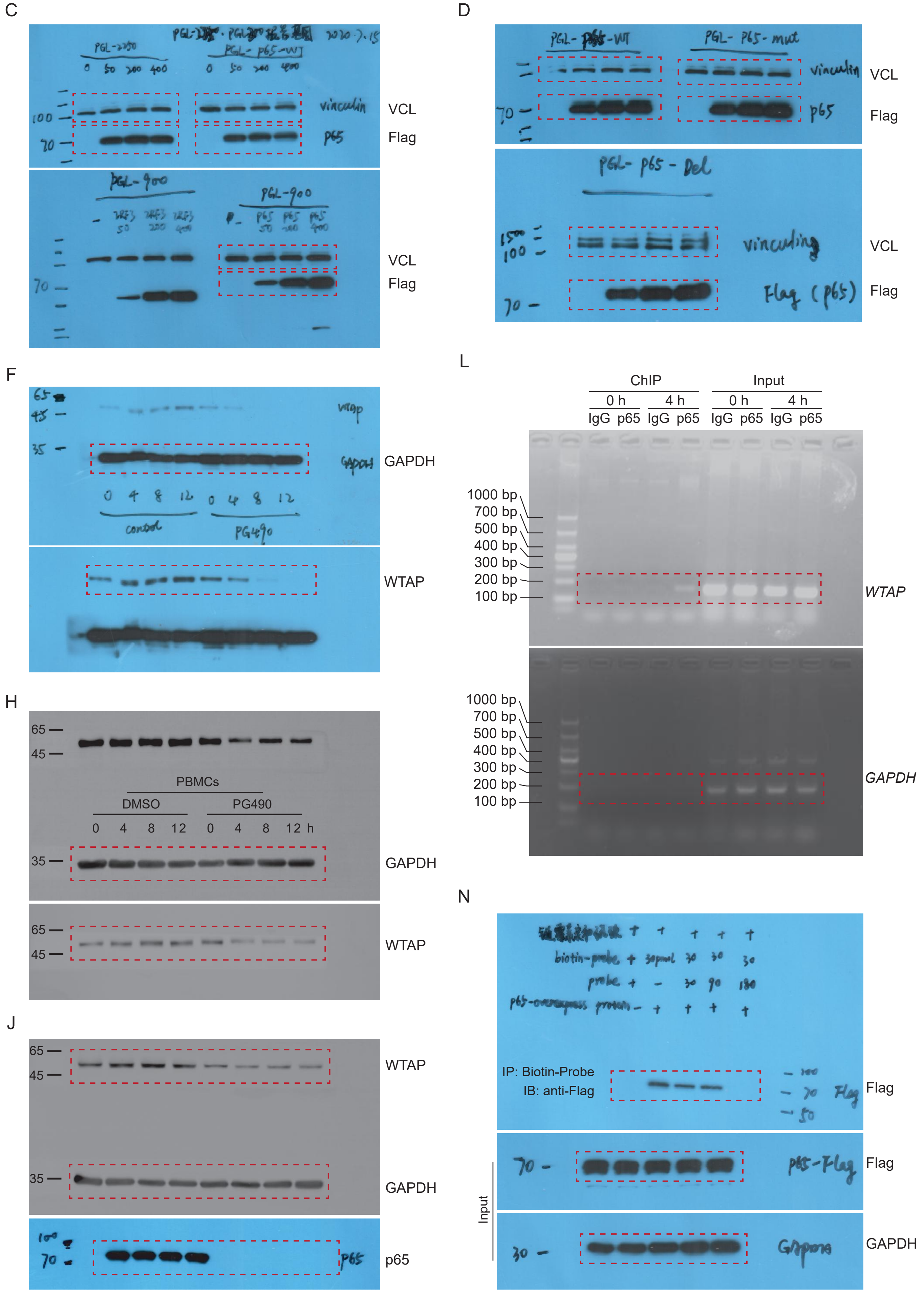

Uncropped blot for Figure 4

E

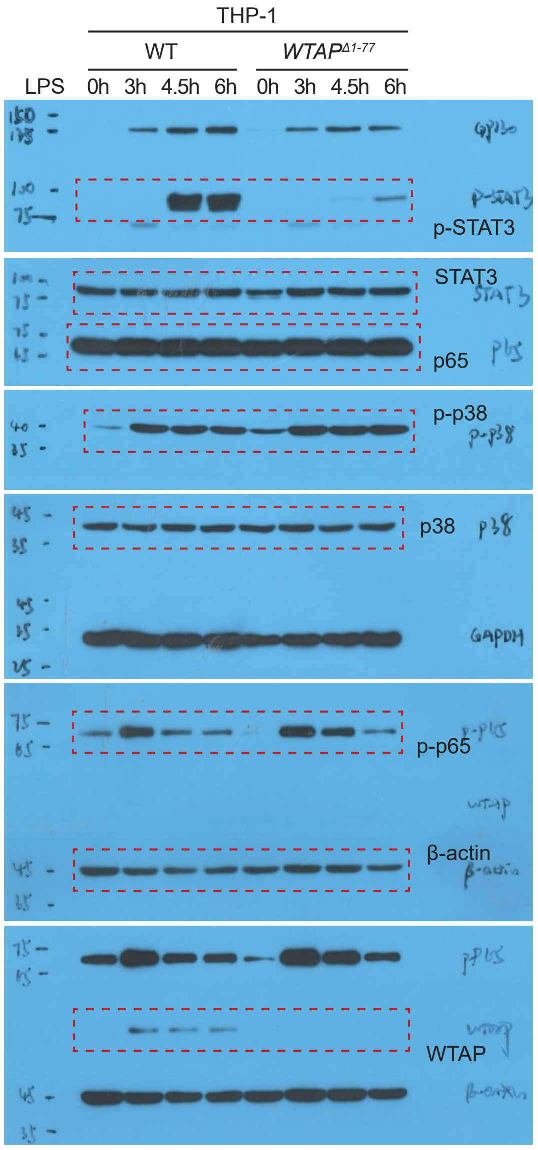

F

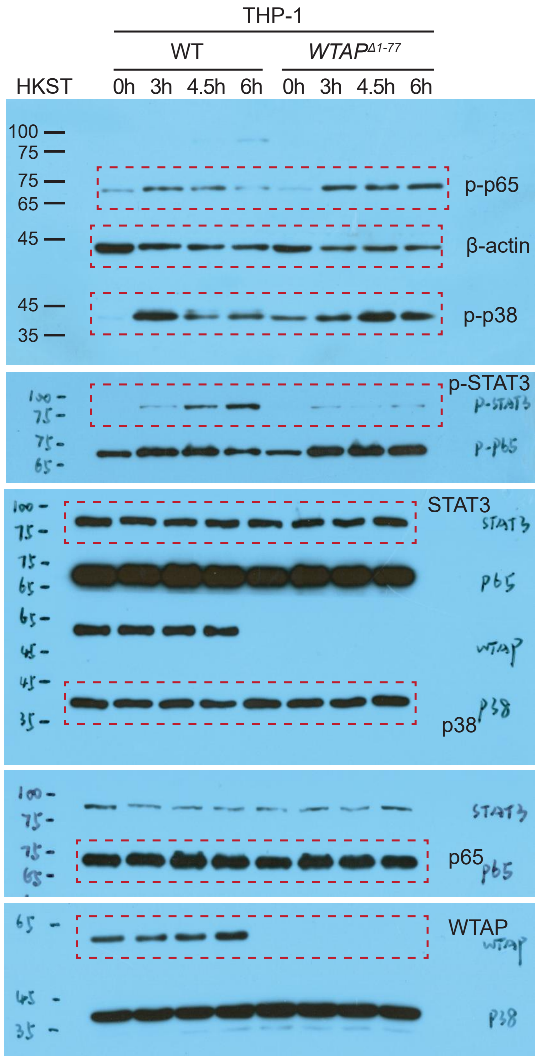

G

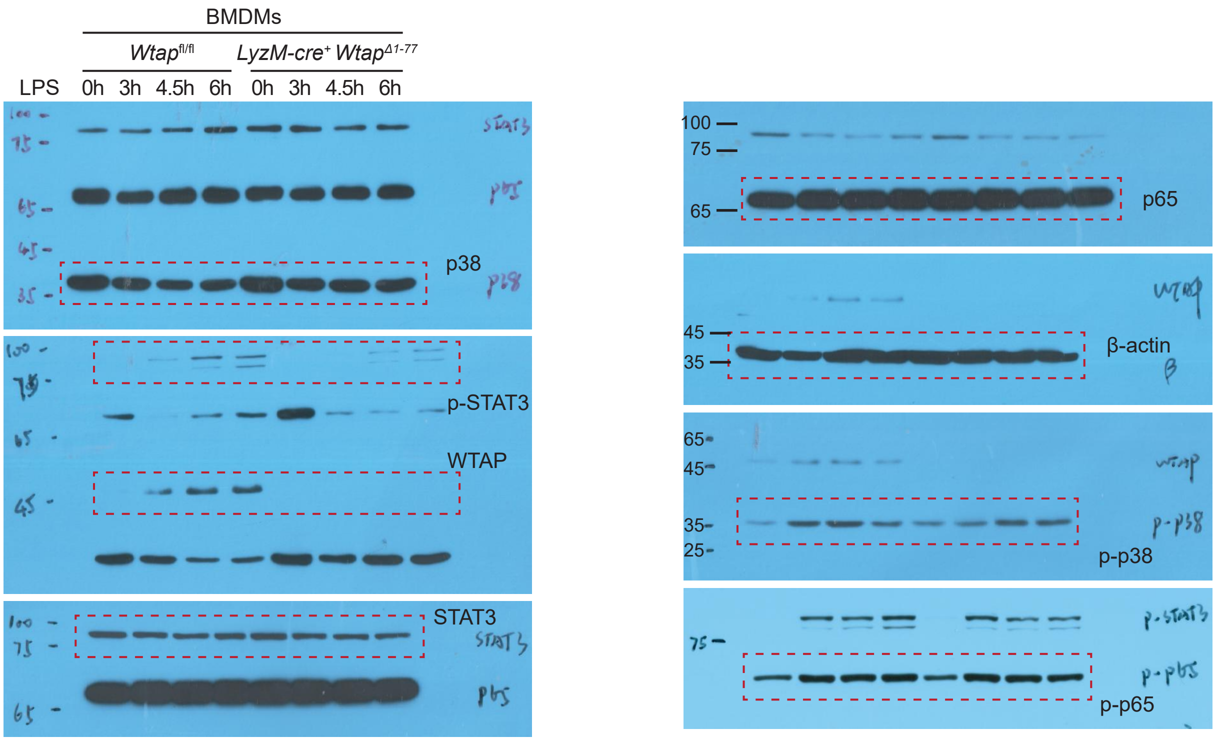

Uncropped blot for Figure 4

H

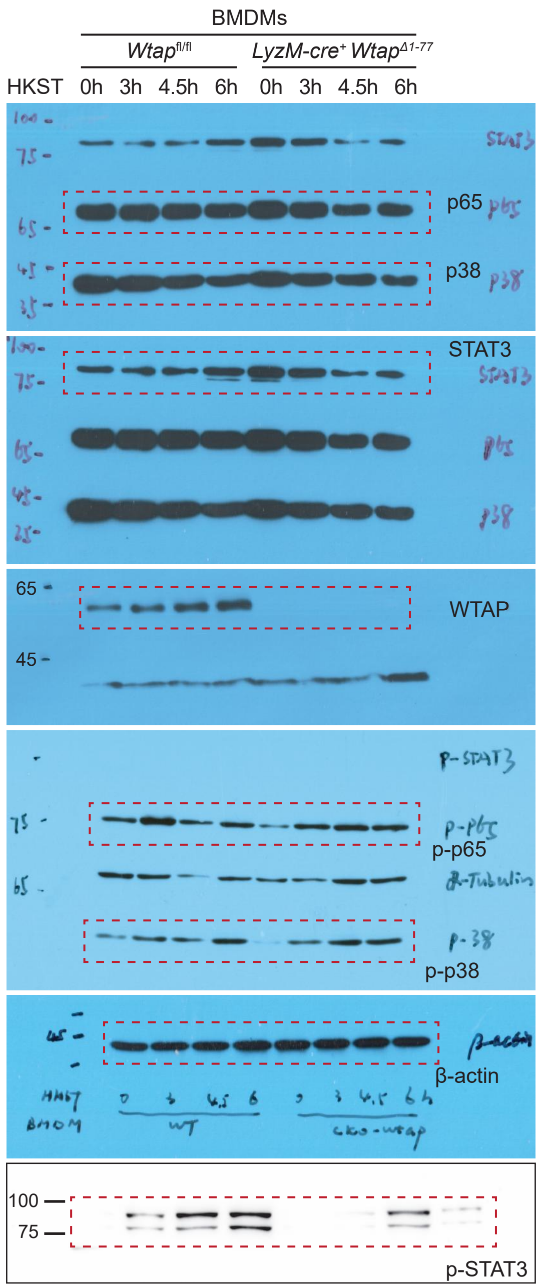

Uncropped blot for Figure 5

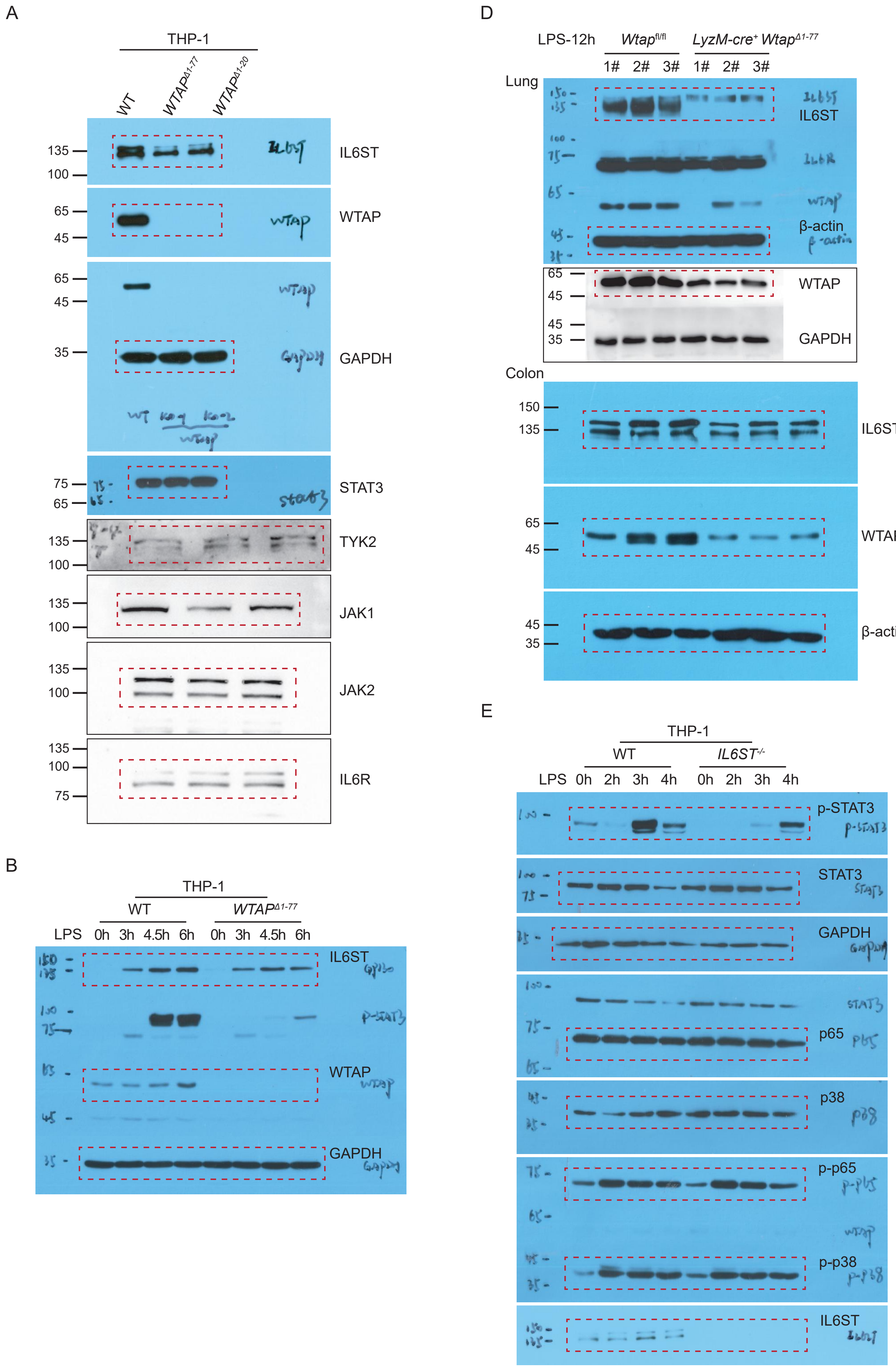

Uncropped blot for Figure 5

N

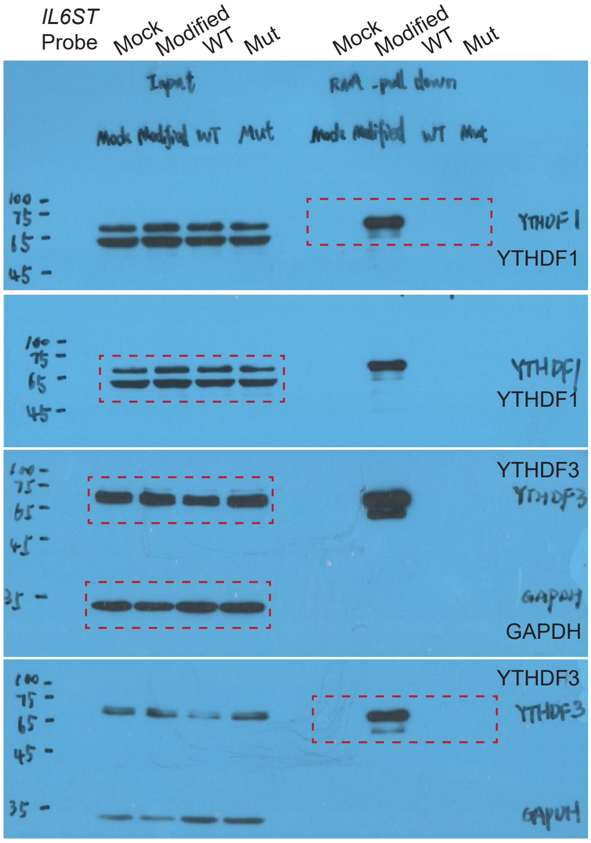

P

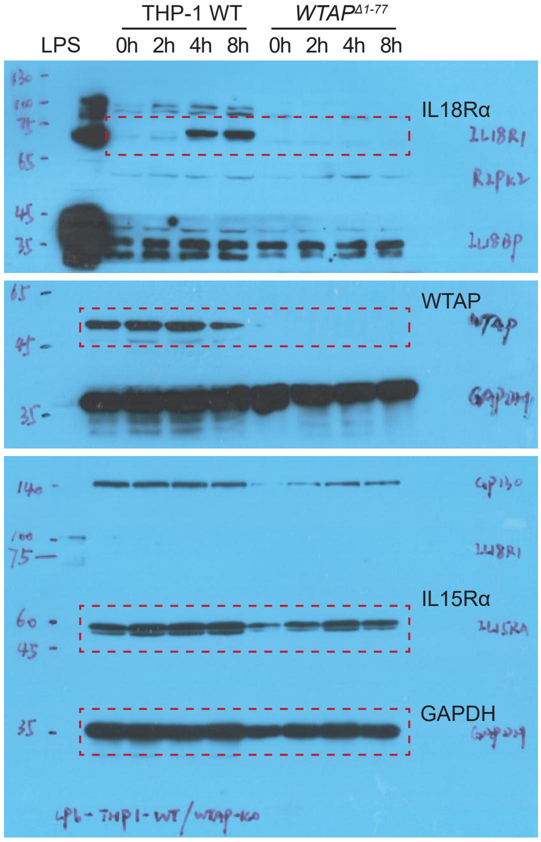

Uncropped blot for Figure 8

D

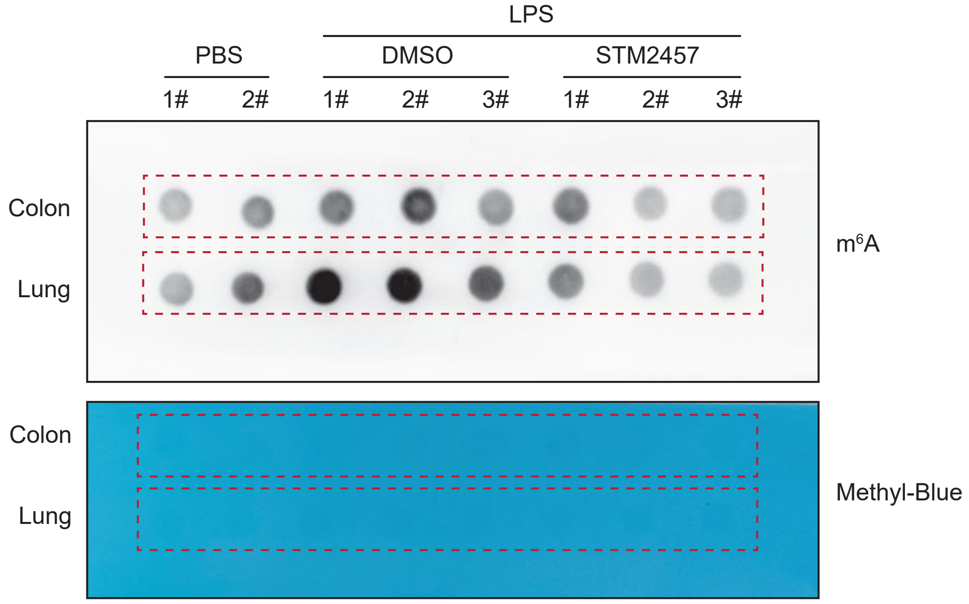

Uncropped blot for Supplemental Figure 2

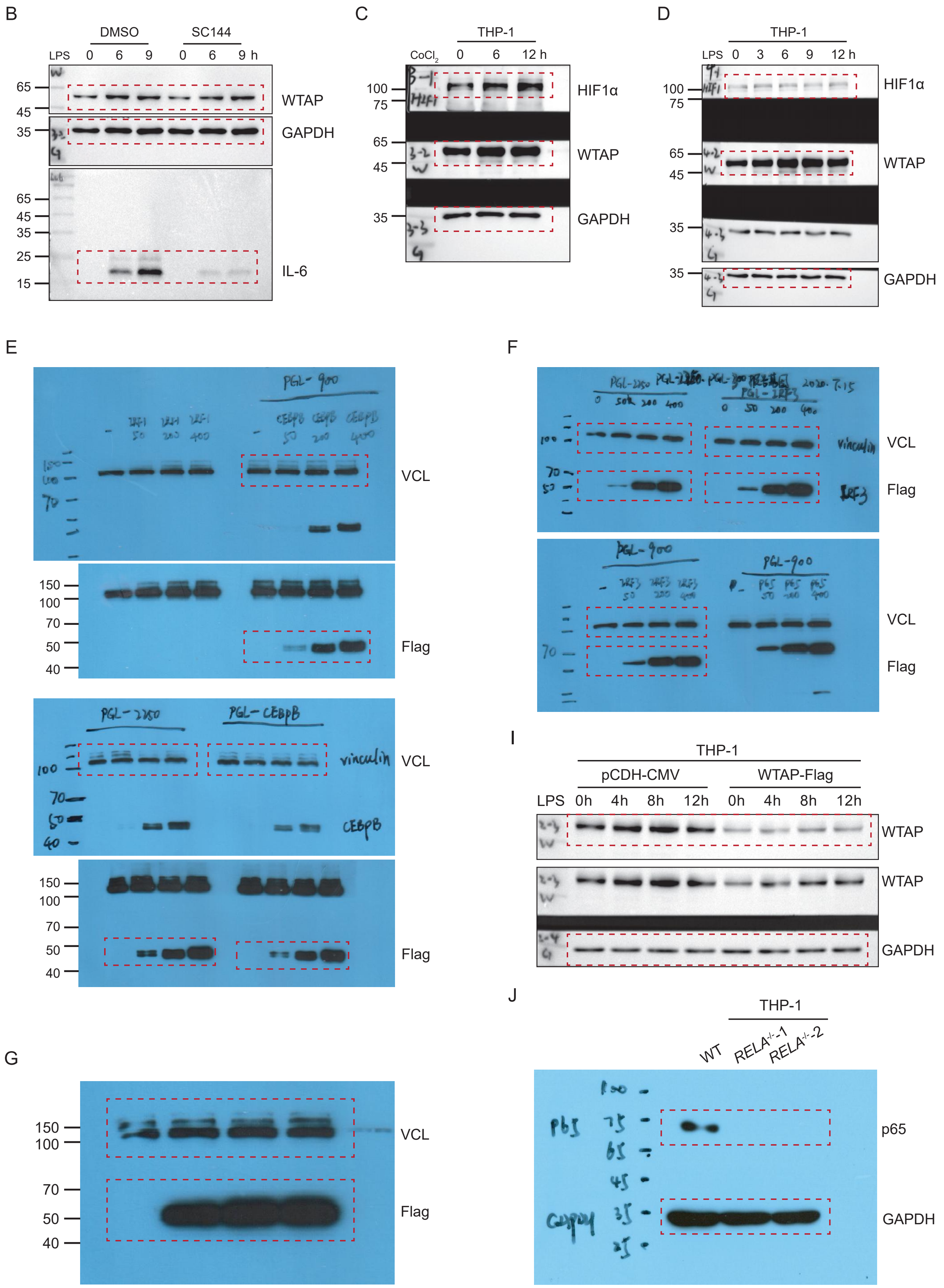

Uncropped blot for Supplemental Figure 2

L

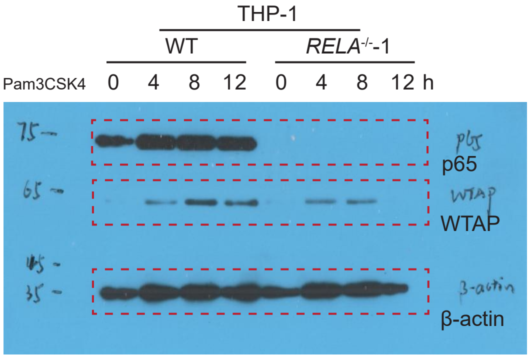

N

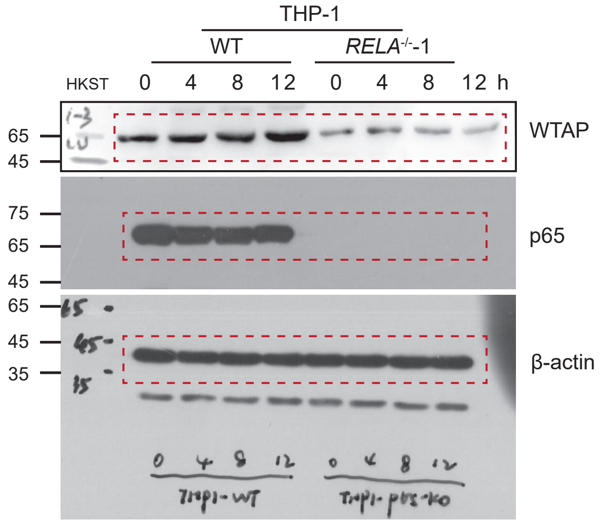

O

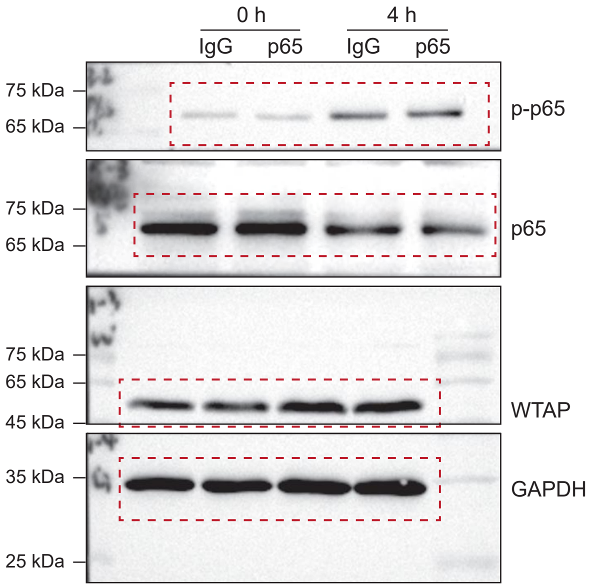

P

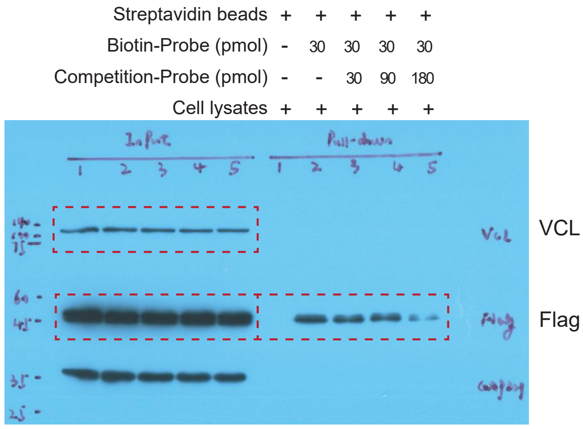

Uncropped blot for Supplemental Figure 3

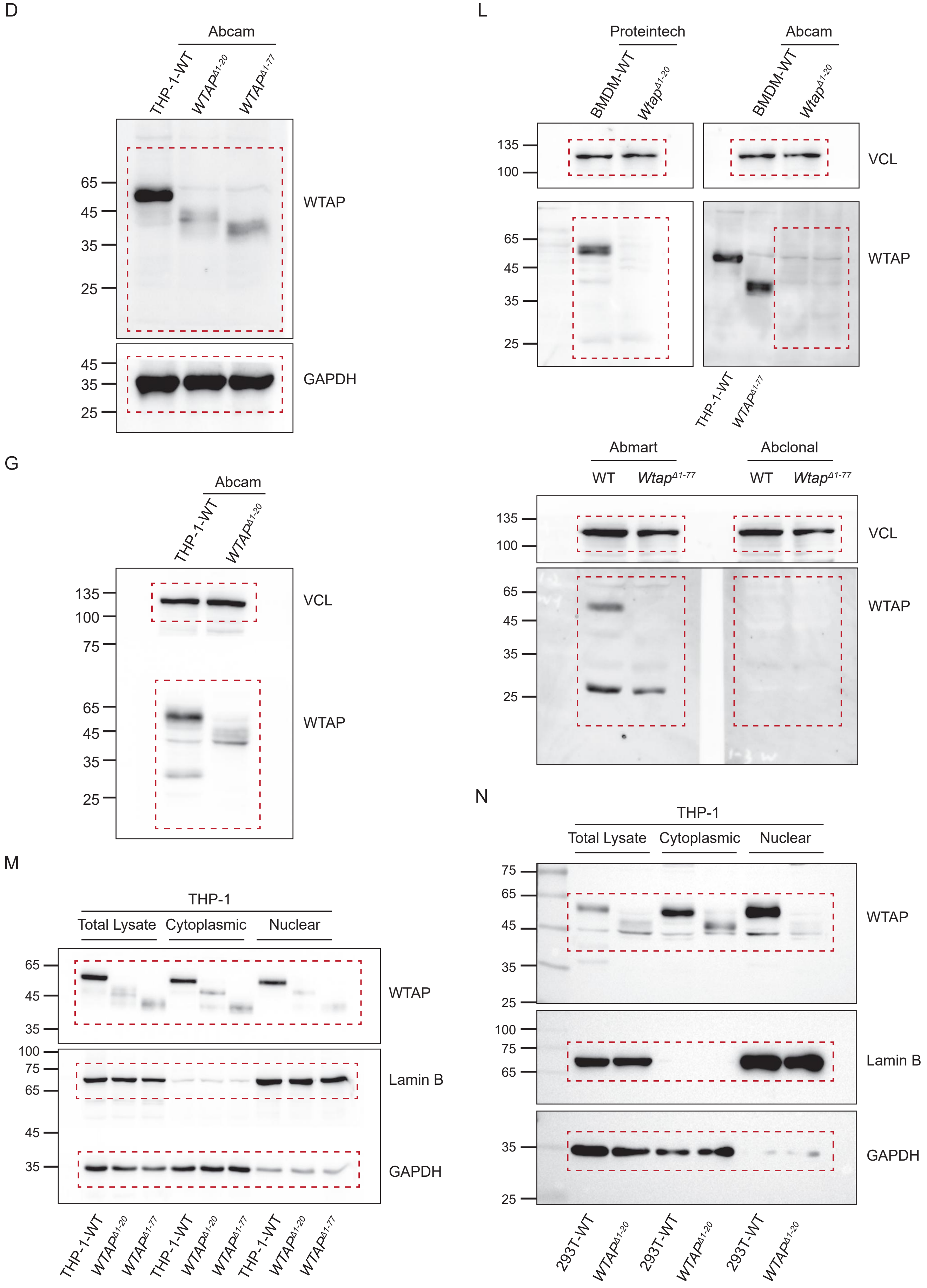

Uncropped blot for Supplemental Figure 5

C

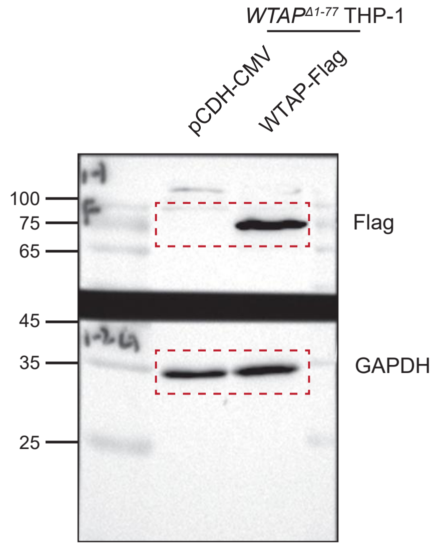

B

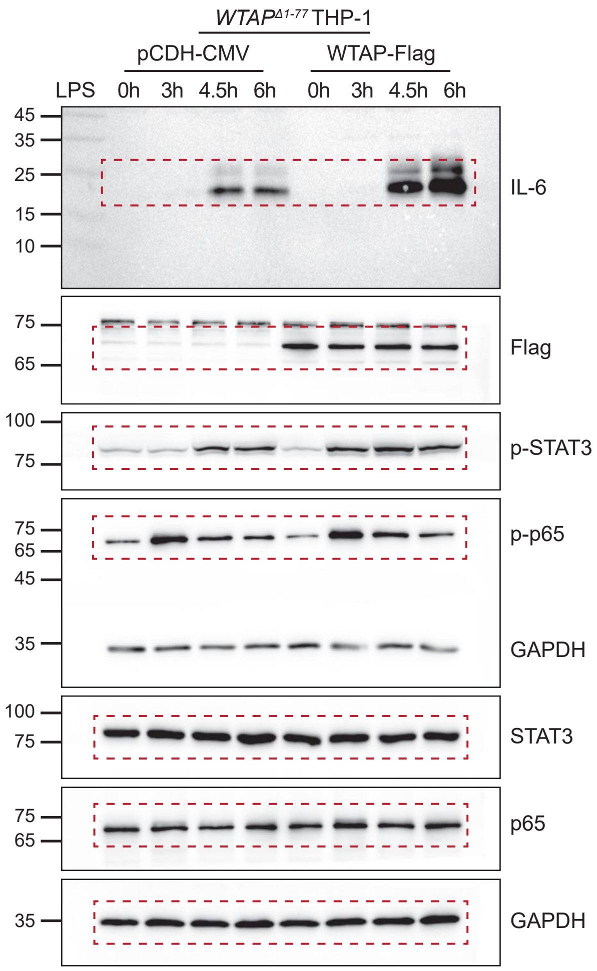

Uncropped blot for Supplemental Figure 7

A

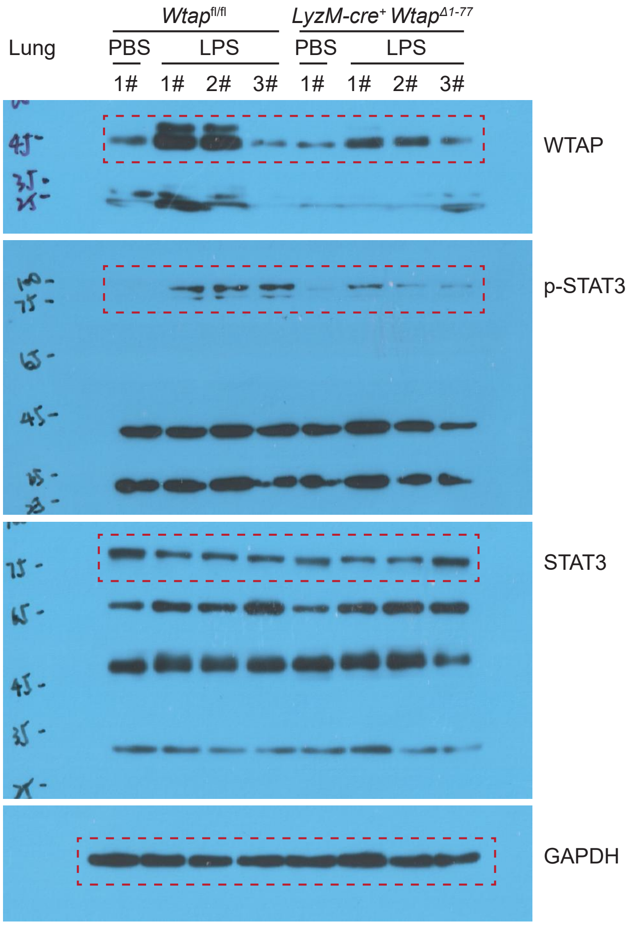

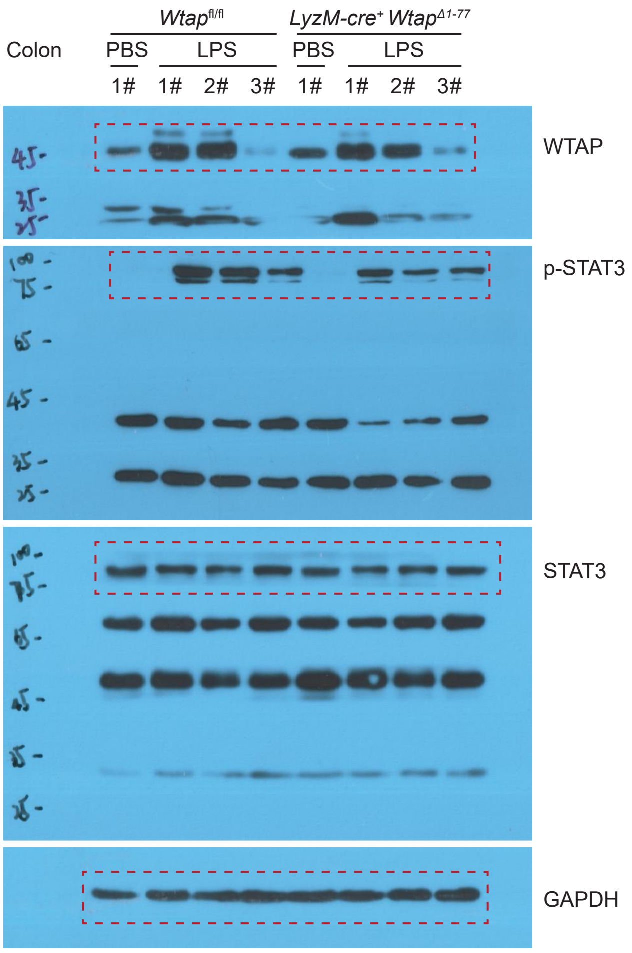

Uncropped blot for Supplemental Figure 9

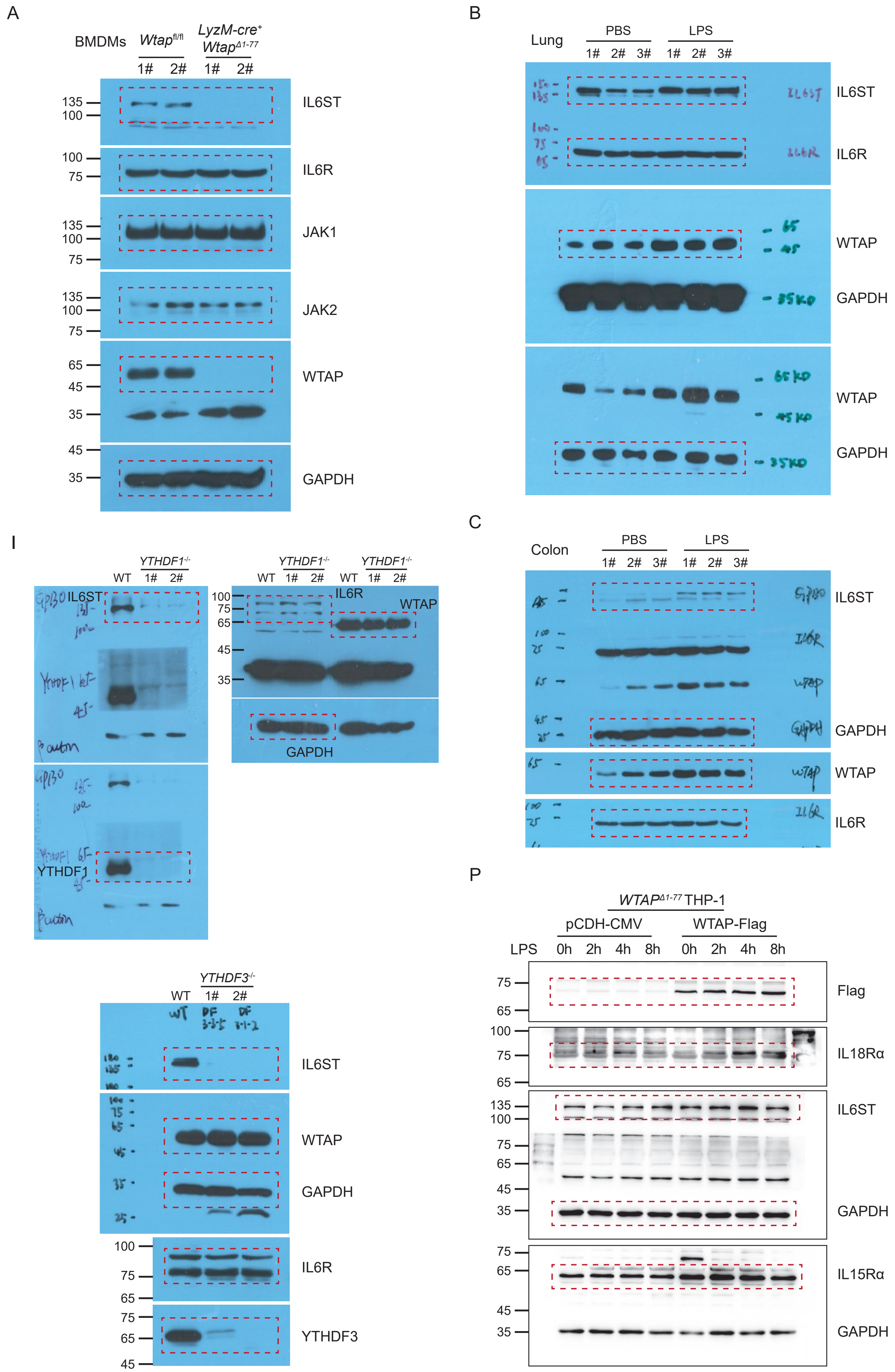

Uncropped blot for Supplemental Figure 10

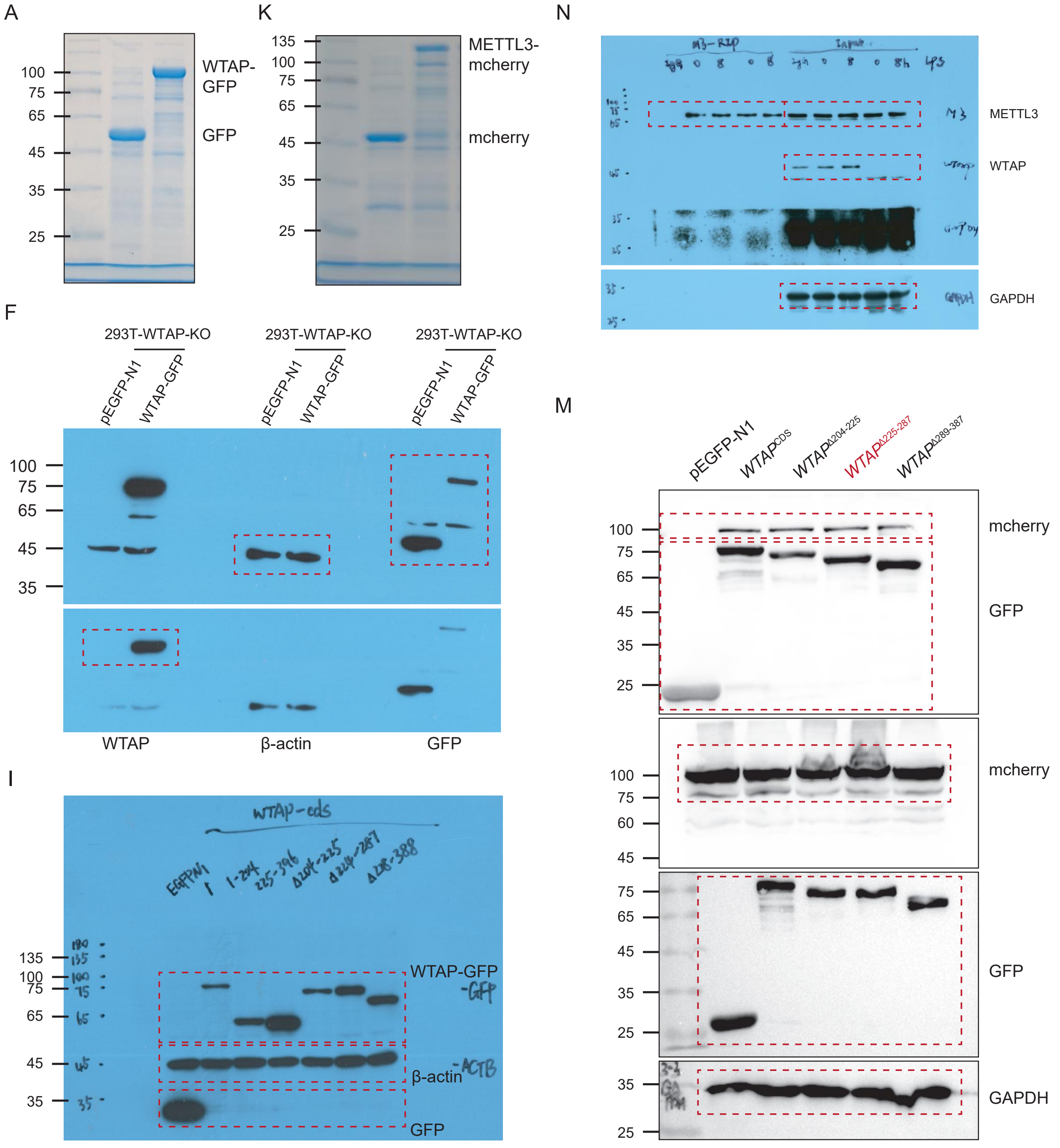

Supplement: Unedited blot and gel images [file jci-134-177932-s031.pdf]
